# Transcriptomic analysis reveals the formation mechanism of anemone-type flower in chrysanthemum

**DOI:** 10.1186/s12864-022-09078-3

**Published:** 2022-12-22

**Authors:** Jiawei Fan, Jialu Huang, Ya Pu, Yajing Niu, Mengmeng Zhang, Silan Dai, He Huang

**Affiliations:** 1 Beijing Advanced Innovation Center for Tree Breeding By Molecular Design, Beijing Key Laboratory of Ornamental Plants Germplasm Innovation & Molecular Breeding, Beijing Laboratory of Urban and Rural Ecological Environment, Key Laboratory of Genetics and Breeding in Forest Trees and Ornamental Plants of Education Ministry, School of Landscape Architecture, National Engineering Research Center for Floriculture, Beijing Forestry University, Beijing, 100083 China; 2 National Bot Garden, Beijing, 100093 China

**Keywords:** Anemone-type, Disc floret, CYC2s, MADS-box, Phytohormone, Cell division, Transcriptome analysis

## Abstract

**Background:**

The ray and disc florets on the chrysanthemum capitulum are morphologically diverse and have remarkably abundant variant types, resulting in a rich variety of flower types. An anemone shape with pigmented and elongated disk florets is an important trait in flower shape breeding of chrysanthemums. The regulatory mechanism of their anemone-type disc floret formation was not clear, thus limiting the directional breeding of chrysanthemum flower types. In this study, we used morphological observation, transcriptomic analysis, and gene expression to investigate the morphogenetic processes and regulatory mechanisms of anemone-type chrysanthemum.

**Result:**

Scanning electron microscopy (SEM) observation showed that morphological differences between non-anemone-type disc florets and anemone-type disc florets occurred mainly during the petal elongation period. The anemone-type disc florets elongated rapidly in the later stages of development. Longitudinal paraffin section analysis revealed that the anemone-type disc florets were formed by a great number of cells in the middle layer of the petals with vigorous division. We investigated the differentially expressed genes (DEGs) using ray and disc florets of two chrysanthemum cultivars, 082 and 068, for RNA-Seq and their expression patterns of non-anemone-type and anemone-type disc florets. The result suggested that the *CYCLOIDEA2* (CYC2s), MADS-box genes, and phytohormone signal-related genes appeared significantly different in both types of disc florets and might have important effects on the formation of anemone-type disc florets. In addition, it is noteworthy that the auxin and jasmonate signaling pathways might play a vital role in developing anemone-type disc florets.

**Conclusions:**

Based on our findings, we propose a regulatory network for forming non-anemone-type and anemone-type disc florets. The results of this study lead the way to further clarify the mechanism of the anemone-type chrysanthemum formation and lay the foundation for the directive breeding of chrysanthemum petal types.

**Supplementary Information:**

The online version contains supplementary material available at 10.1186/s12864-022-09078-3.

## Background

**Fig. 1:**
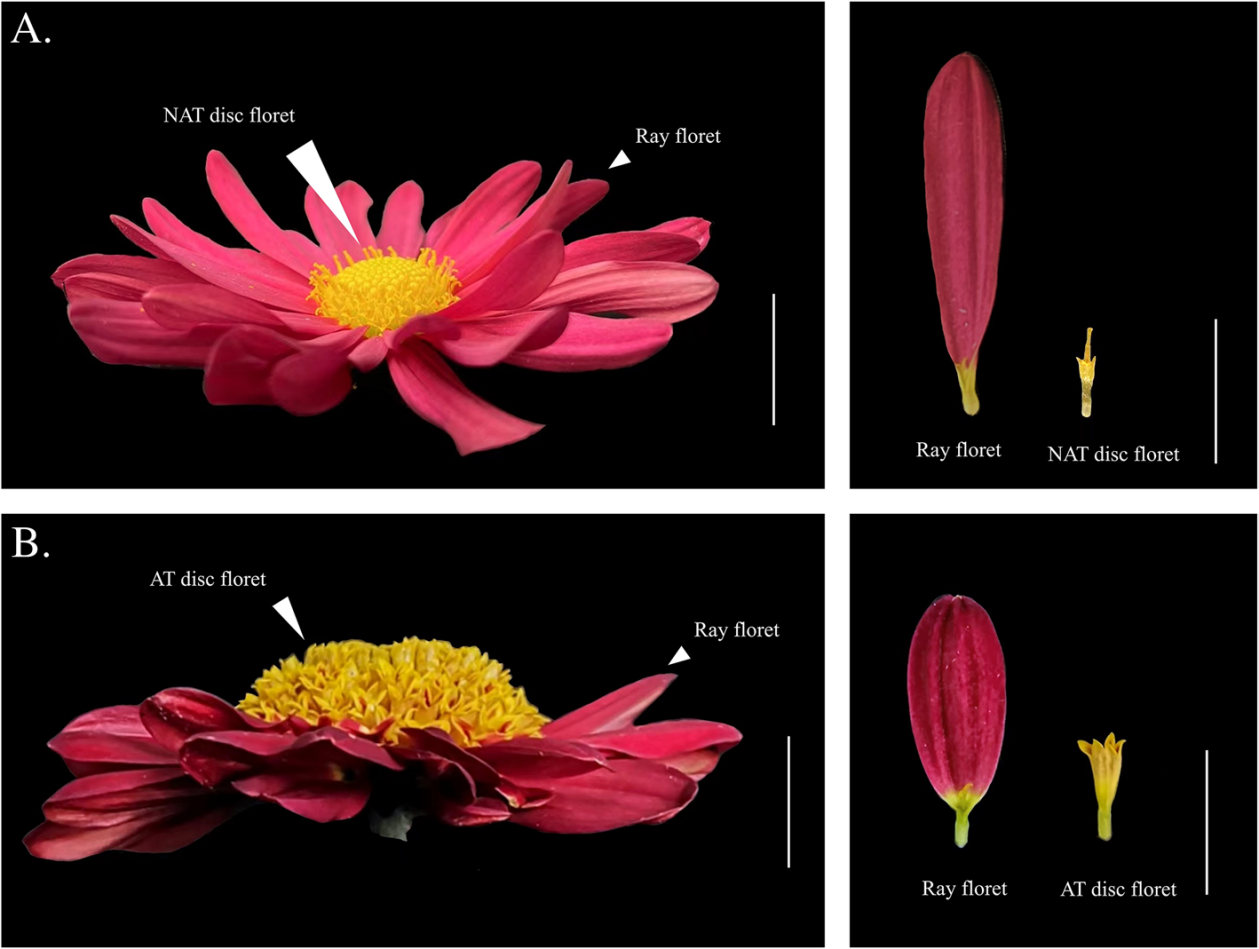
Anemone-type (068) and non-anemone-type (082) chrysanthemum at blooming stage. Scale bar = 1cm **A**. ray floret and AT disc floret of anemone-type chrysanthemum. **B**. ray floret and NAT disc floret of non-anemone-type chrysanthemum. AT: anemone-type NAT: non-anemone-type

In higher plants, variation in inflorescence structure, flower type, and flower symmetry is vitally essential to plant reproduction. Meanwhile, variable petal morphology can improve the ornamental value of plants as a breeding goal of many horticulturists [1]. Chrysanthemum is a famous ornamental plant, and its inflorescence is formed by a large number of disc florets surrounded by ray florets [2]. The flower type is a significant trait strongly correlated with the attractiveness of the chrysanthemum for ornamental purposes and commercial value. The ray and disc florets of chrysanthemums differ in their morphology, particularly in terms of the symmetry and fertility of their corollas. Ray florets are usually unisexual with a noticeable bilaterally symmetrical petal structure in the corolla tube, while the disc florets are fertile bisexual with radially symmetrical corolla [3]. Unlike other chrysanthemum flower types, the anemone-type inflorescence features elongated and colored disc florets (Fig. 1), which combine to make its center particularly conspicuous [4]. The elongated disk florets display brilliant pigmentation and have a long flowering time [5]. Current research suggested that the anemone-type chrysanthemum evolved from the basic petal-type with a high degree of genetic differentiation.The cells of the inner layer of anemone-type disc florets do not degenerate and could divide continuously, so the petals are more developed than those of ordinary disc florets, which may be the main reason for the formation of anemone type in chrysanthemum [6]. Considerable research efforts have been focused on anemone-type chrysanthemums, such as the determination of pollen germination analysis [7], genetic analysis [8], and QTL mapping [9], while the molecular regulatory mechanisms of anemone-type chrysanthemum were rarely reported.

The morphogenesis of flowers in higher plants mainly goes through the following stages (1) the initiation of the floral primordia (2) the determination of the identity of the floral organs (3) the differentiation, proliferation, and expansion of floral organ cells. The final size of the petals is determined at a later time of development [10]. Much work has focused on revealing the regulatory mechanisms underlying the morphogenesis of floral organs. It is well established that the ABC model determines the identification of the floral organs and controls flower organ shape at a later developmental stage [11, 12]. Most ABC-identity genes belong to the MADS-box TFs. In Asteraceae, the ABC model is also involved in regulating capitulum development. In addition to controlling floral organ characteristics, some ABC genes also regulate the size of floral organs. In the *Phalaenopsis* orchid, the flower size was smaller in E-class gene *PeSEP3* silenced plants than in mock-treated, whereas the epidermal cell size was decreased in both height and width in petals, to ether with increased cell density [13]. Suppression of the E-class genes *GRCD5* expression in Gerbera resulted in a substantial reduction in the length of its transition flowers [14, 15]. In chrysanthemum, most studies on the MADS-box family have been reported on the regulation of flower formation and flowering time [16–18]. Barely no systematic research concerning the mechanism of ABC genes in regulating the formation of different types of disc florets has been published yet.

Studies in Asteraceae plants such as *Gerbera hybirda*, *Helianthus annuu*s, *Senecio vulgaris* and *Chrysanthemum* × *morifolium* have revealed that *TEOSINTE BRANCHED1* / *CYCLOIDEA* / *PROLIFERATING* (TCP) family transcription factor CYCLOIDEA2-like (CYC2-like) are critical for the differentiation of ray and disc florets. *CYC2*-like gene is not only involved in regulating symmetry on petals but also in the cellular proliferation, expansion, and differentiation of the marginal and central layer of florets [19–24]. In *Antirrhinum majus*, *CYC2*-like gene promotes dorsal petals’ growth at the late stages of flower development [25]. In *Gerbera hybrida* capitulum, overexpression of *GhCYC2* caused the petals of disc florets to be significantly elongated, similar to the ray and trans flowers. In another *GhCYC2* transgenic line, the petals of disc florets were fused in a tubular shape and appeared to seem like an anemone-type flower [19]. In *Helianthus annuus,* the ray florets mutated into disc-floret morphology due to the insertion of a transposable element (TE) in the *HaCYC2c* promoter and exons [21]. Overexpression of *HmCYC2cM* increased the petal length of disc florets [26], and the shape of the flower is similar to the anemone-type flower shape. This suggests that *CYC2-*like gene figured prominently in forming different types of disc floret in Asteraceae. These studies in Asteraceae expand our understanding of the formation mechanism of the anemone-type chrysanthemum.

The overall developmental process of petals is regulated by hormones. Auxin, ethylene, gibberellin, abscisic acid, and jasmonate are known to affect flower opening and petal expansion [27]. Auxin is a direct signal for the initiation of petal primordia at a very early stage. Genes related to auxin synthesis, transport, and response mutants can affect petal morphogenesis [28, 29]. In Arabidopsis, *AUXIN RESPONSE FACTOR* (*ARF*) *ARF8* mutant lines *arf8-3* showed an inhibition effect on the elongation of petals, measurements of the petal surface showed that petals of *arf8-3* were significantly larger compared with the wild-type plants, and the *arf6 arf8* double-null mutant flowers were arrested as infertile closed buds with short petals [30–32]. In addition to auxin, other phytohormones are important mediators in floral organ morphogenesis. For example, in *Gerbera hybrida*, the WIP-type zinc finger protein GhWIP2 acts as a transcription inhibitor to regulate the levels of gibberellin, abscisic acid, and auxin, thereby inhibiting petal cell expansion and affecting the final morphology of gerbera ray florets [33]. Jasmonates (JA) also have a crucial effect on flower development, in *Arabidopsis thaliana*. JA acts as a morphogenesis signal that regulates the processes of cell expansion and petal growth and mainly participates in the regulation of late-stage floral organ development [34, 35]. A mutant type *pdm* with degenerated petals was found in Chinese cabbage, and the petal phenotype of *pdm* returned to wild type in response to exogenous JA application by spraying inflorescences and petals [36].

Chrysanthemum (*Chrysanthemum* × *morifolium*) is an economically valuable ornamental plant of Asteraceae [37, 38]. However, the molecular basis of anemone-type disc floret morphogenesis in chrysanthemum remains largely unknown. Here, we combined morphological and transcriptome analysis to investigate the developmental characteristics and the differentially expressed genes (DEGs) between anemone-type and non-anemone-type disc florets. Our research extends the knowledge into the floral development mechanism of different forms of disc florets, providing a theoretical basis for the directed breeding of chrysanthemum petal types.

## Result

### Developmental process and cytological characteristics of the non-anemone-type and anemone-type disc floret

**Fig. 2:**
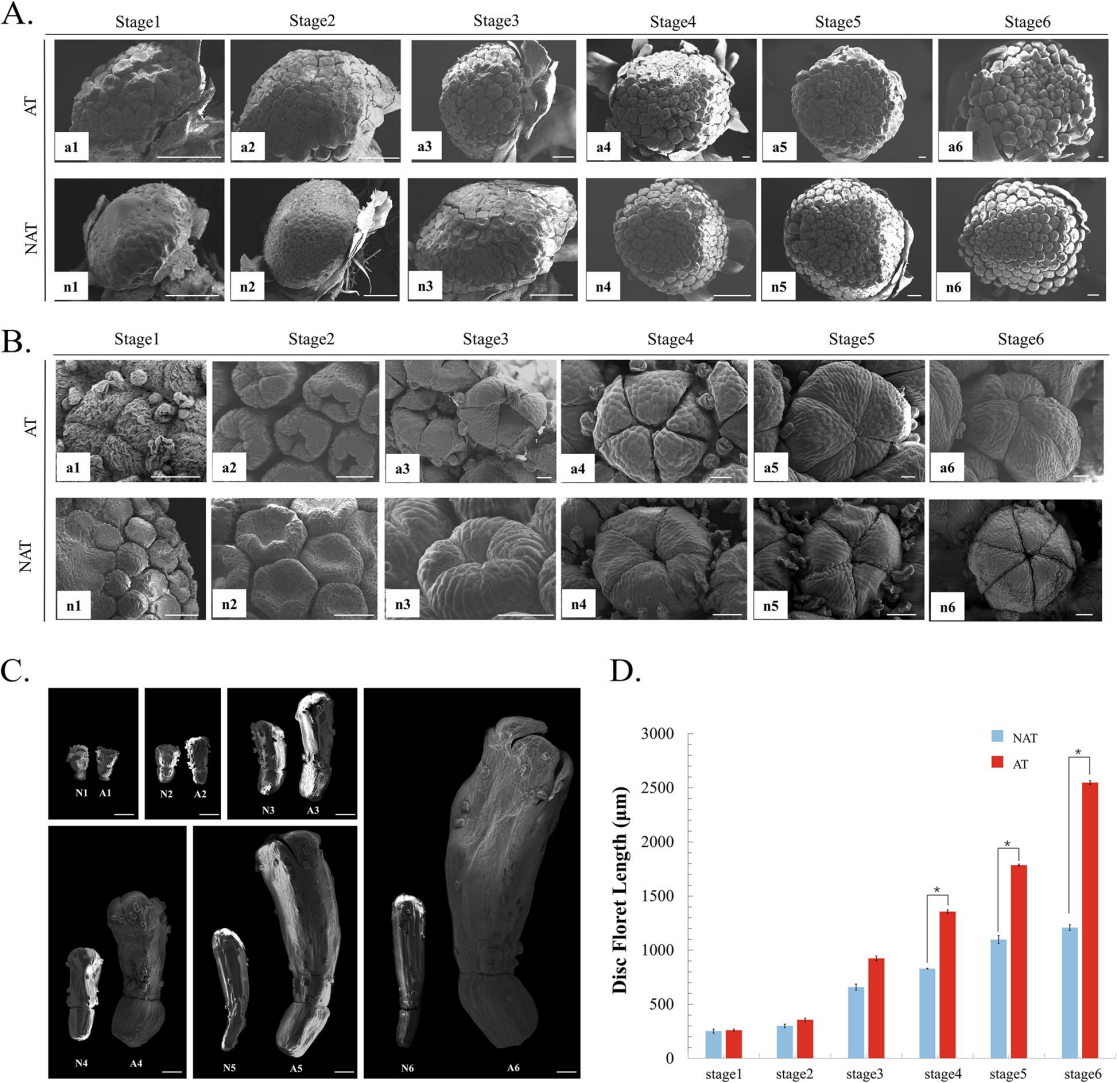
SEM of the capitulum and disc florets of non-anemone-type and anemone-type chrysanthemums. **A**: SEM images of the capitulum. Scale bar = 200 μm. **B**: SEM analysis of the corolla lobes comparing two types of disc florets. Scale bar = 50 μm. **C**. SEM observation NAT disc floret and AT disc floret. Scale bar = 500 μm. **D**. Length measurement of NAT disc floret and AT disc floret at six developmental stages. (t-test, n ≥ 10 *P* < 0.05) N (n): non-anemone-type chrysanthemum A (a): anemone-type chrysanthemum

The anemone-type (AT) flower is one of the most important chrysanthemum flower types which has a prominent flower center, composed of elongated disk florets, while the non-anemone-type (NAT) inflorescence consists of a set of hermaphroditic central disk florets, which are considered the primitive form (Fig. 1). To determine the critical stage when phenotypic differences appear between AT and NAT chrysanthemum, scanning electron microscopy (SEM) and paraffin section techniques were used to observe the AT and NAT flowers (Fig. 1) at six different developmental stages (Table 1, Additional file 1: Figure S1.). SEM observations revealed that at stage 1 (Fig. 2A, a1, n1), both AT and NAT chrysanthemum capitulum had initially formed ray and disc flower primordia, the same as in other Asteraceae [39–41]. When the capitulum developed to stage 3, the corolla lobes of the NAT disc floret and AT disc floret appeared (Fig. 2A, a2, n2, a3, n3). After stage 4, the developmental levels of the two types of disc floret were synchronized (Fig. 2A, a4, n4, a5, n5). Comparing the developmental process of the AT and NAT chrysanthemum capitulum showed no difference in the positioning of the NAT disc floret and AT disc floret on the capitulum (Fig. 2A, a6, n6).

**Table 1:** A schedule for development stages of disc florets in *Chrysanthemum* × *morifolium*

Stage no	Stage name	Landmarks of morphological
Stage 1	Formation stage of disc floret primordia	Disc floret primordia continued to generate a spiral centripetal pattern
Stage 2	Early-stage corollas lobes formation	The apical part of the floret primordia has further sagged and the corolla begins to form
Stage 3	End-stage of corollas lobes formation	The corollas lobes of disc florets began to form
Stage 4	Early-stage of disc floret elongation	The apical corolla started to expand
Stage 5	The middle stage of disc floret elongation	The apical corolla started to elongate
Stage 6	Stage of disc floret persistent elongation	Floret corolla continuously elongated and expanded

**Fig. 3:**
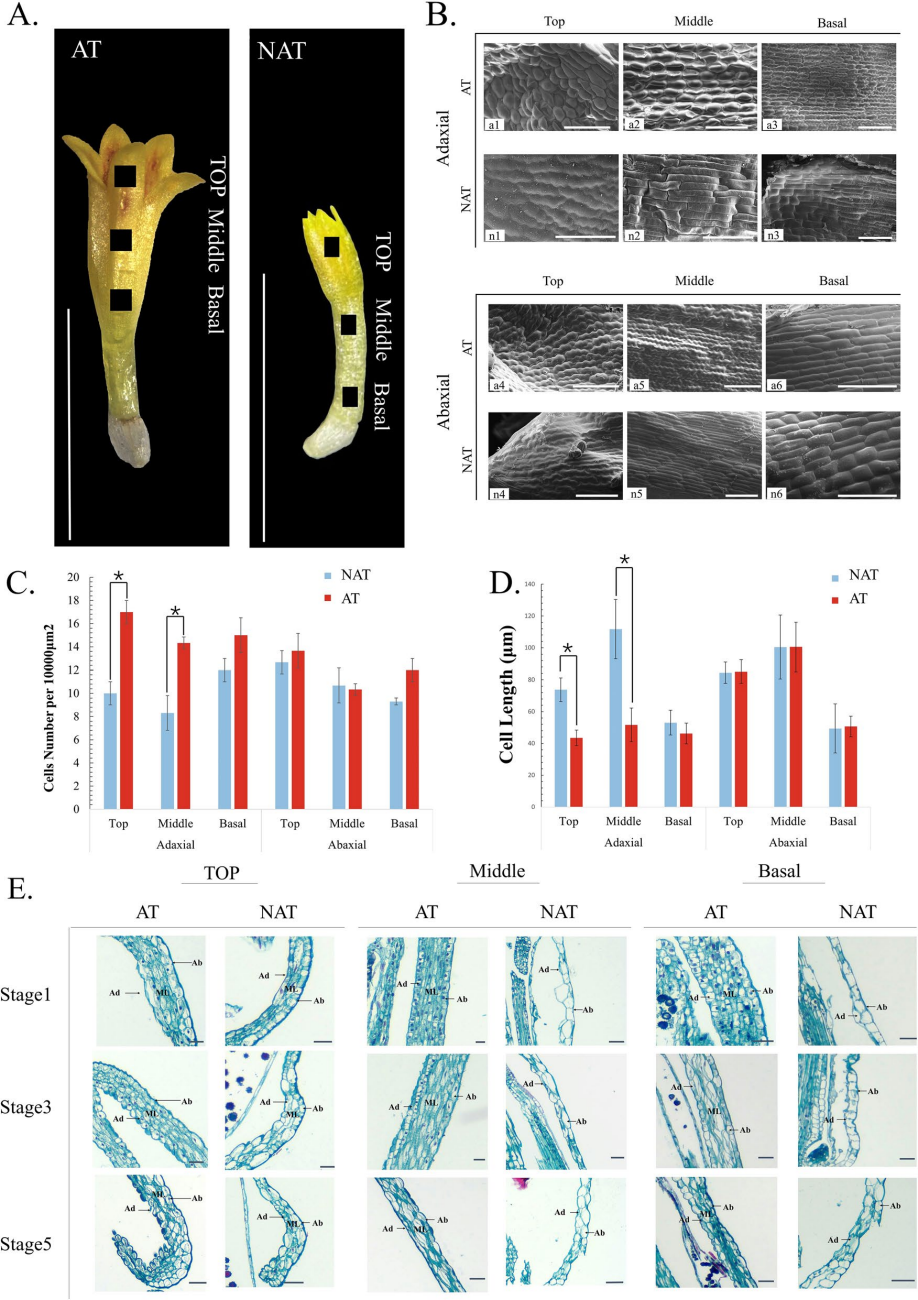
Morphological observation, cell counting and length measurement of NAT disc floret and AT disc floret. **A**. Observation areas of the top, middle, and basal region of NAT disc floret and AT disc floret at blooming stage. Scale bar = 5 mm. **B**. SEM observation of the cell morphology of the top, middle, and basal of NAT disc floret and AT disc floret. Scale Bar = 100 μm. **C**. Cell counting in different parts of the two types of florets. (t-test, n ≥ 15 *P* < 0.05) **D**. Measurement of cell lengths. Values are presented as the mean ± SE. (t-test, n ≥ 25, *P* < 0.05). **E**. Histology of petals from NAT disc floret and AT disc floret. longitudinal sections of disc floret from the top, middle and basal parts of stage 1, stage 3, and stage 5 were observed. Ad: Adaxial side Ab: Abaxial side ML: mesophyll layer cells. Scale Bar = 10 μm

**Fig. 4:**
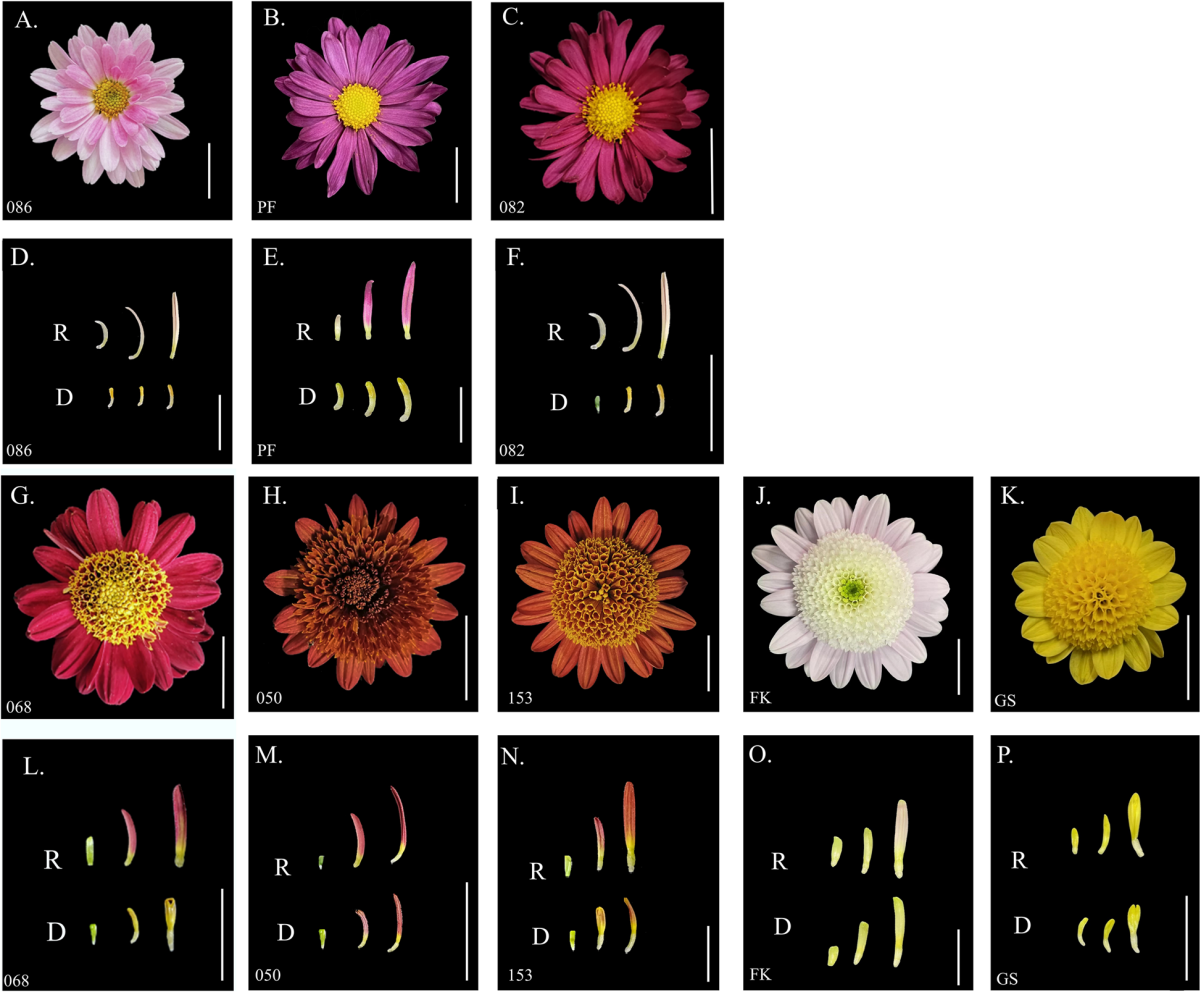
Characterization of non-anemone type and anemone type chrysanthemums. **A**-**C**: capitulum of non-anemone type chrysanthemums (082, PF, 086). **D**-**F**: ray(R) and disc florets (R) of R4, R5, R6, D4, D5, D6 open stages of non-anemone type chrysanthemums (082, PF, 086). **G**-**K**: capitulum of anemone-type chrysanthemums (068, 050, 153, FK, GS) heads. **L**-**P**: ray (R) and disc florets (D) of the R4, R5, R6, D4, D5, D6 open stages of anemone-type chrysanthemums. R: Ray florets D: Disc Florets. Scale bar = 1 cm

**Fig. 5:**
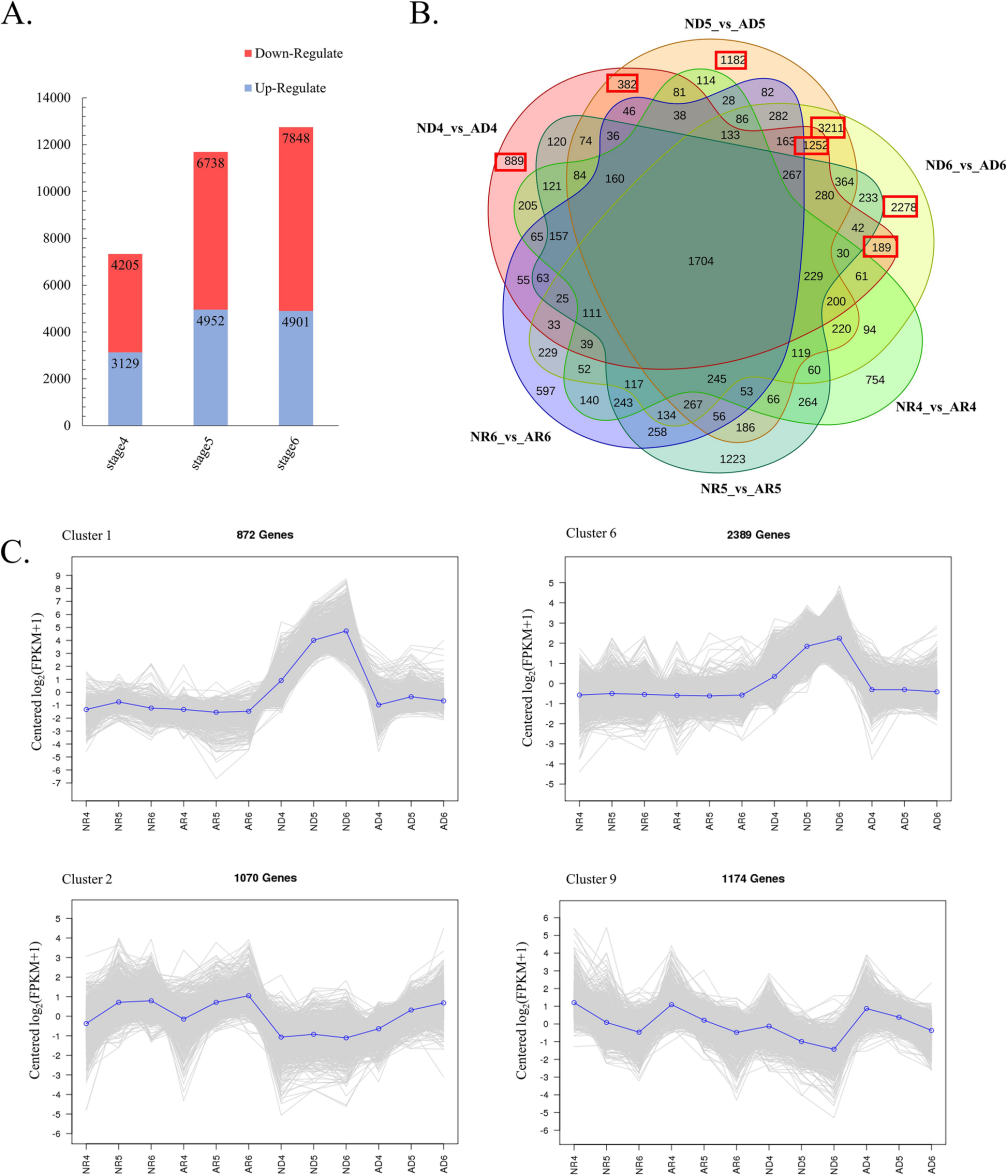
Analysis of DEGs in NAT disc floret and AT disc floret. **A**. The number of up- or down-regulated genes in stage 4, stage 5, and stage 6. **B**. Venn diagram showing the distribution of unigene expression revealed by paired comparison. Red boxes indicate genes that specifically expressed in disc floret as we expected. **C**. The four clusters of DEGs with different expression patterns by K-means. NR: non-anemone-type ray floret AR: anemone-type ray floret ND: non-anemone-type disc floret AD: anemone-type disc floret

**Fig. 6:**
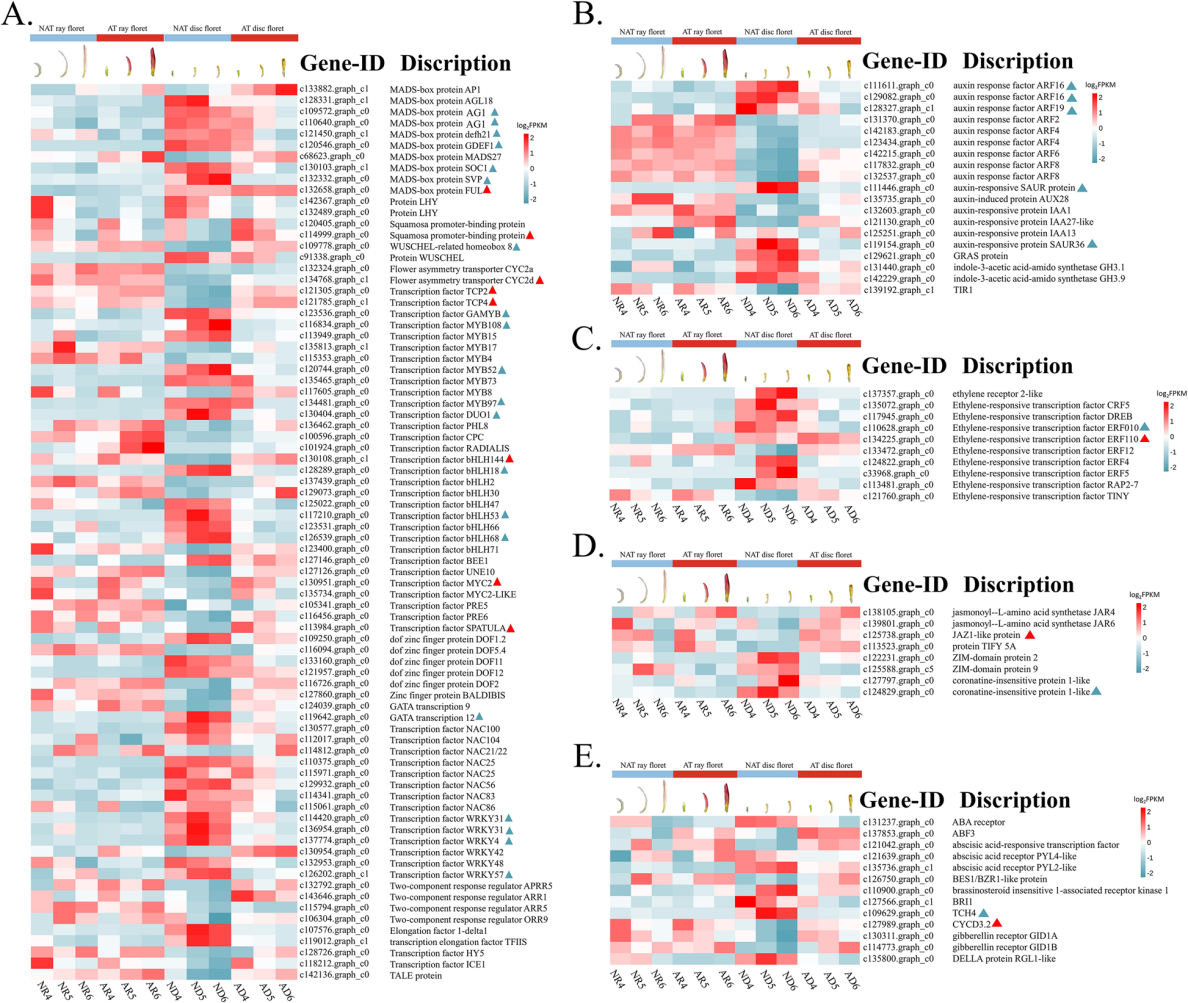
Heatmap of DEGs in petals of NAT disc floret and AT disc floret in the three floral development stages. The bar represents the scale of the expression levels of each gene (log_2_FPKM) in the three stages as indicated by red/blue rectangles. Red rectangles represent the up-regulation of genes, and blue rectangles represent down-regulation. All genes in this list have a differential expression *P*-value < 10^−5^. Red triangles represent key up-regulated genes and green triangles represent key down-regulated genes. **A**. Transcriptions factor involved in two types of disc florets. **B**. DEGs of auxin signal transduction pathway. **C**. DEGs of ethylene signal transduction pathway. **D**. DEGs of jasmonate signal transduction pathway. **E**. DEGs of abscisic acid, brassinolide, and gibberellin signal transduction pathway

The morphogenesis of AT and NAT disc florets was further observed (Fig. 2B). The lengths of NAT disc floret and AT disc floret were almost the same during stage 1-stage 3 (Fig. 2 C, D). At stage 1, the NAT disc floret was still primordia while the lobe had already emerged at the apical of the AT disc floret. At stage 2, the center primordia of the NAT disc floret sagged inward. The corollas lobes of the AT disc floret were further elongated (Fig. 2B, a2, n2), at stage 3, the corollas lobes of the AT disc floret and NAT disc floret were grown completely, and their length was the same (Fig. 2B, a3, n3). At stage 4, the AT disc floret had grown much longer than the NAT disc floret (Fig. 2C). After stage 4, the AT disc floret petal grew rapidly and eventually became about twice the length of NAT disc floret (Fig. 2C). From stage 4 to stage 6, The NAT disc floret and AT disc floret only differed in petal length (Fig. 2D).

To investigate the cytological differences between AT and NAT disc floret, the adaxial and abaxial cells of disc florets were observed by scanning electron microscopy (SEM) (Fig. 3). The petal of AT and NAT disc floret at blooming stage (Fig. 1) was divided into the top, middle and basal parts (Fig. 3A). TheAT and NAT disc floret have differed more on the adaxial side (Fig. 3B). On the adaxial side, the top cells of the NAT disc floret showed elongated serrate cells (Fig. 3B, n1), of which AT disc floret was the small circular cell (Fig. 3B, a1). The middle cells of the NAT disc floret showed elongated striped cells (Fig. 3B, n2), which in AT disc floret showed subcircular (Fig. 3B, a2). The basal medial cells of NAT disc floret were rectangular (Fig. 3B, n3), while AT disc floret had long serrated cells (Fig. 3B, a3). The difference between the abaxial cells of NAT and AT disc floret was mainly in the middle region, where the NAT disc floret cells were elongated striped cells (Fig. 3B, n5), and the AT disc floret cells were long serrated (Fig. 3B, a5). In cell counting and a cell length measurement of three parts on two sides, the difference was in the top and middle of the adaxial side (Fig. 3C, D). The reduced cell size was accompanied by increased cell number on the same surface area, suggesting that the increased cell number and decreased cell length of the AT disc floret may contribute to the longer petal length than that of the NAT disc floret.

Furthermore, from the cross-paraffin section of the NAT disc floret, we found that the structure only consisted of adaxial and abaxial cells with thick cell walls and thin cytoplasm, and the cells were not vigorous (Fig. 3E). The structure of the AT disc floret consists of multilayered cells, including adaxial and abaxial cells and loose-leaf mesophyll cells which have dense cytoplasm and varying morphology with vigorous division activity. As the flowering gradually progressed, the multilayered cells in the top and middle parts of the AT disc floret gradually became thinner, while there was no obvious change in the NAT disc floret.The cells always remained only one or two layers. It indicated that the formation of AT disc floret is closely related to cell division.

### Transcriptome sequencing and functional annotation of non-anemone-type and anemone-type chrysanthemum

To deeply investigate the molecular mechanism of NAT disc floret and AT disc floret formation and identify genes with a differential expression that only in NAT disc floret and AT disc floret, not in ray floret, RNA-Seq of the ray and disc floret of two types of chrysanthemums was carried out. The ray and disc floret petals of 082 and 068 cultivars at stage 4, stage 5, and stage 6 (Fig. 4C, G) provided the template for RNA-Seq analysis. The statistics of 36 samples sequencing data evaluations are shown in Additional file 2: Table S1 and Additional file 3: Figure S2.

36 samples were sequenced and filtered. A total of 252.16 Gb Clean Data was generated with a Q30 (percentage of sequences with sequencing error rates lower than 0.1%) of 92.63%-97.29%. 100,230 high-quality unigene were obtained after filtration. De novo assembly and splicing of the mean size was 769 bp. The value of N50 was 1,369. 24,722 unigenes were above 1 kb in length. Transcript abundance (estimated from fragments per Kilobase of exon model per Million mapped fragments, FPKM) was highly correlated between replicate samples were highly correlated with each other. A total of 53,900 unigenes were annotated based on BLASTx (E-value < 1 × 10^−5^) and HMMER (E-value < 1 × 10^−10^) searches against public databases, including COG, GO, KEGG, KOG, Pfam, Swiss-Port, eggNOG, and Nr (Additional file 4: Figure S3.). 26,345 (48.88%) were annotated in KOG, 31,109 (57.72%) were annotated in Pfam, 30,774 (57.09%) were annotated in Swissprot, 38,710 (57.72%) were annotated in eggNOG, and 51,700 (95.92%) were annotated in Nr database. The functions of the predicted unigenes were classified using GO, COG, and KEGG assignments. 42,588 unigene (79.01%) were annotated by GO into three major categories (cellular components, molecular functions, and biological process) and 52 subcategories. 11,644 unigene (21.60%) were annotated in COG and classified into 25 functional groups. A total of 31,319 (58.43%) unigene were annotated to 49 KEGG pathways, among which the Plant-pathogen interaction pathway was the most significant, followed by the plant hormone signal transduction pathway.

### Identification of DEGs in the developmental process of NAT disc floret and AT disc floret

To further identify the key genes affecting the morphogenesis of the anemone-type chrysanthemum, we used the pairwise comparison method to screen genes that differed only in NAT disc floret and AT disc floret. FPKM values were used to measure the expression level of genes. The DEGs showing an FDR ≤ 0.001 and a |log2(ratio)|≥ 2 were selected for different comparisons. DEGs were identified by comparing transcriptomes between NAT disc floret and AT disc floret at the same development stage. In comparison of stage 4, stage 5, and stage 6 of disc florets (Fig. 5A), 7,334 DEGs were found in stage 4, of which 3,129 genes were up-regulated and 4,205 genes were down-regulated. 11,609 DEGs were found in stage 5, of which 4,952 genes were up-regulated and 6,738 genes were down-regulated. There were 12,749 DEGs in stage 6, of which 4,901 genes were up-regulated and 7,848 genes were down-regulated. The number of DEGs in the three stages gradually increased, suggesting that the formation of AT disc floret is probably due to differential gene expression at a later stage. By a pairwise comparison method, we identified genes that expressed specifically differently in disc florets but not in ray florets (Fig. 5B). A total of 9,383 DEGs were detected with the above comparisons. These DEGs might contain important factors affecting the shape of AT disc florets.

To explore the molecular differentiation during flower development, these DEGs were further functionally classified through Gene Ontology (GO) analysis. They were classified into 17 GO classes that fell into the category ‘Biological processes’, 15 into the category ‘Cellular components’, and 12 into the ‘Molecular function’. The unigene KEGG annotation was aimed at DEGs from the above comparisons. In ND4_VS_AD4, 2567 genes were annotated in the KEGG database involving 131 pathways (Additional file 5: Figure S4., Additional file 6: Table S2). The major pathways identified were ‘Plant hormone signal transduction’, ‘Starch and sucrose metabolism’, ‘MAPK signaling pathway’, and ‘Phenylpropanoid biosynthesis’. In ND5_VS_AD5, 5701 DEGs were mapped on 132 pathways. In ND6_VS_AD6, 133 pathways involved 6,399 DEGs. In the comparison of these two groups, the primary pathways were identified as plant hormone signal transduction, starch and sucrose metabolism, and phenylpropanoid biosynthesis. All three comparative results indicated that hormones have a pivotal role in petal development.

### Cluster analysis of DEGs

The overall expression pattern of DEGs was shown on the clustering map with K-means cluster analysis, 9,383 DEGs were classified into 9 clusters (Additional file 7: Figure S5.), of which 4 Clusters matched the expression trends we were expecting (Fig. 5C). The expression trends of Cluster 1 (872 DEGs), and Cluster 6 (2,389 DEGs) were similar, showing a consistent expression in ray florets, while high expression in NAT disc floret and low expression in AT disc floret. Cluster 2 (1070 DEGs) and Cluster 9 (1,174 DEGs) showed low expression in NAT disc floret and high expression in AT disc floret. In the flower development process, transcription factors (TFs) play an important role in regulating flower organ morphogenesis and 130 differential expression TFs were finally detected by K-means clustering analysis.

### DEGs related to the morphogenesis of AT disc floret

To identify the genes involved in regulating the morphology of the NAT and AT disc floret, differentially expressed TFs were identified using pairwise comparison and K-means cluster analysis (Fig. 6A), which included MADS-box, TCP, ARF, ERF, MYB, bHLH, NAC and WRKY genes family, etc. (All FPKM and annotation are shown in Additional file 8: Table S3 and Additional file 9: Table S4).

In the many families of floral development transcription factors, MADS-box TFs are essential for determining floral organ development and petal elongation. In AT disc floret, c132658.graph_c0 (*FUL*), c133882.graph_c1 (*AP1*), and c68623.graph_c0 (*MASD27*) were up-regulated, whereas c109572.graph_c0 (*AG1*), c121450.graph_c1 (*defh21*), c120546.graph_c0 (*GDEF1*), c130103.graph_c1 (*SOC1*), and c132332.graph_c0 (*SVP*) were down-regulated. The FPKM differential of MADS-box transcription factors was relatively large in NAT disc floret and AT disc floret, we hypothesized that the MADS-box TFs are likely to play a negative regulatory role (Fig. 6A).

TCP TFs also vital in regulating the development of NAT disc floret and AT disc floret. The present results revealed that c121305.graph_c0 (*TCP2*), c121785.graph_c1 (*TCP4*), and c134768.graph_c1 (*CYC2d*) were up-regulated in the AT disc floret while c132324.graph_c0 (*CYC2a*) was down-regulated. c134768.graph_c1 (*CYC2d*) showed the most significant differences in FPKM in NAT disc floret and AT disc floret. Among all DEGs, bHLH TFs accounted for the majority, of which 10 were up-regulated and 6 were down-regulated in the AT disc floret. A nearly 30-fold change was recorded in FPKM of c130951.graph_c0 (*MYC2*) and c117210.graph_c0 (*bHLH53*). The DEGs mentioned above are likely to be the candidates with the greatest effect on AT disc floret morphogenesis (Fig. 6A).

Petal growth depends on cell proliferation and division during early development stages but is controlled by cell expansion during late flower development, and this process is mainly influenced by plant hormones [42]. Phytohormone receptors or phytohormone responders are induced by phytohormone signaling pathways such as auxin (IAA), gibberellin (GA), ethylene (ET), and jasmonic acid (JA). In chrysanthemum, genes related to phytohormone pathways were found to be differentially regulated during petal elongation in NAT disc floret and AT disc floret based on FPKM. The results showed the most DEGs between NAT disc floret and AT disc floret were genes involved in auxin signal transduction (Fig. 6B). c131370.graph_c0 (*ARF2*), c142183.graph_c0 (*ARF4*), c142215.graph_c0 (*ARF6*)*,* and c117832.graph_c0 (*ARF8*) were up-regulated in AT disc floret while c129082.graph_c0 (*ARF16*) and c128327.graph_c1 (*ARF19*) were down-regulated. c129082.graph_c0 (*ARF16*) had remarkable differences in FPKM and may be a negative regulator of AT disc floret formation. The ERFs mainly involved the ethylene-responsive transcription factor AP2/ERF (Fig. 6C). 7 of the 14 transcription factors were upregulated in the AT disc floret. The FPKM of c124822.graph_c0 (*ERF4*) and c33968.graph_c0 (*ERF5*) was the most different. The JA pathway mainly included the *JAR4*, *JAZ1*-like, which was up-regulated in AT disc floret, and c124829.graph_c0 (*COI1*), c122231.graph_c0 (*ZIM-domain protein 2*), and c125588.graph_c5 (*ZIM-domain protein 9*) were down-regulated in NAT disc floret (Fig. 6D). Genes in GA, ABA, and BR pathway also show a difference between NAT disc floret and AT disc floret. Moreover, it is worth noting that genes are related to cell division and elongation in BR pathway c109629.graph_c0 (*TCH4*) and c127989.graph_c0 (*CYCD3.2*) was founded to express differently in AT and NAT disc florets (Fig. 6E), suggesting cell division and elongation might have a vital effect in the formation of AT flower.

The zinc finger protein family has been reported to regulate petal development in *A. thaliana* [43, 44]. In the present study, eight zinc finger proteins were found. Three were up-regulated and five were down-regulated in the AT disc floret, which has six DOF transcription factors. c119642.graph_c0 (*GATA12*) had the biggest difference in FPKM (Fig. 6A). The results showed that these genes might help AT disc floret petals grow.

### Validation of key DEGs in multiple cultivars

**Fig. 7:**
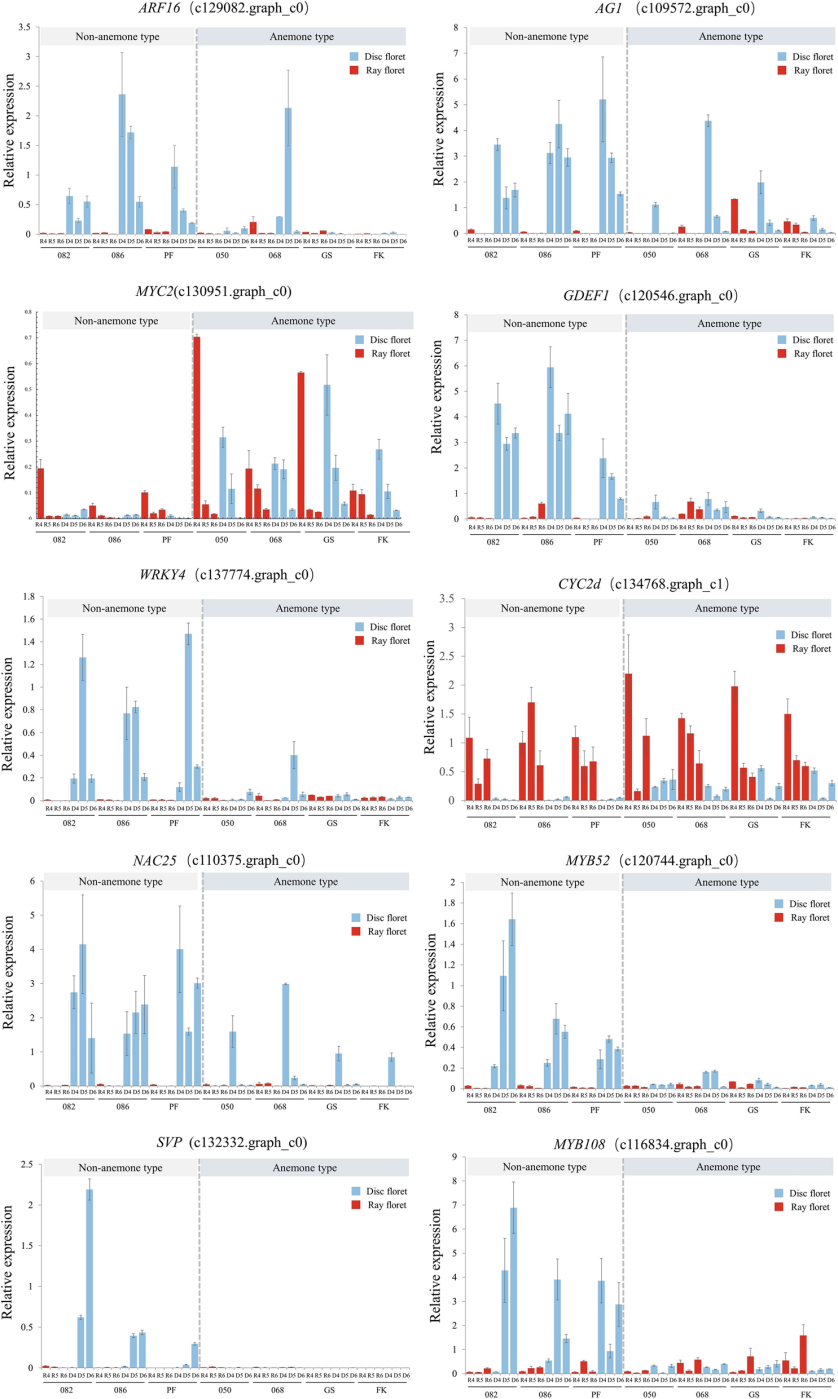
Expression patterns of transcription factors in three non-anemone and four anemone-type chrysanthemums. R: Ray floret D: Disc floret R4-R6: ray floret at different opening stages. D4-D6: disc floret at different opening stages

To validate the RNA-seq data and investigate the expression patterns of key DEGs, we first used RT-PCR analysis in two NAT (082, 086) and three AT (050, 068, 153) chrysanthemum cultivars. RT-PCR analysis obtained 23 genes that appeared to be differentially expressed in NAT disc floret and AT disc floret (Full-length blots/gels are presented in Additional file 10: Figure S6, Additional file 11: Figure S7). These genes were selected for further validation by qRT-PCR in three NAT chrysanthemums (082, 086, PF) and four AT chrysanthemums (050, 068, GS, FK). From the qRT-PCR results, twelve genes were found not to be differentially expressed in other cultivars (Additional file 12: Figure S8.), and it was presumed that these genes might be related to other factors such as flower color and resistance which were not universal. Finally, we obtained 10 genes that appeared to be differentially expressed in NAT disc floret and AT disc floret (Fig. 7). The FPKM of 10 DEGs are shown in Additional file 13: Figure S9.

Among these differentially expressed genes in disc floret, the expression level of *CYC2d* (c134768.graph_c1) and *MYC2* (c130951.graph_c0) were significantly higher in AT disc floret, but lower in NAT disc floret. *ARF16* (c129082. graph_c0), *GDEF1* (c120546.graph_c0), *MYB52* (c120744.graph_c0), *MYB108* (c116834.graph_c0), and *WRKY4* (c134774.graph_c0) were significantly low expressed in each development stage of the AT disc floret than in NAT disc floret at the same period. The expression pattern of *AG1* (c109572.graph_c0) and *NAC25* (c110375.graph_c0) in NAT disc floret and AT disc floret showed that the expression was the same in stage 4 and lower in stage 5 and stage 6 in AT disc floret than in NAT disc floret. It suggested that it plays a negative regulatory role in the elongation of petals in the later stages. From their expression patterns, we also found that the expression pattern of the disc floret of 068 was different from other anemone-type chrysanthemums cultivars because its AT disc floret petals were shorter, and the petal structure was not as obvious as several other cultivars. *SVP* (c132332.graph_c1) showed extremely low expression in ray floret and AT disc floret, while higher expression in NAT disc floret may presumably determine the formation of petal structure. The above results further suggest that these genes are likely to be involved in the morphogenesis of NAT and AT disc floret.

## Discussion

### Anemone-type traits are mainly formed at the later stages of flower development

The disc florets of NAT and AT chrysanthemums developed from disc floret primordia positioned within the capitulum. Their early ontogenetic processes were the same, the morphological differences appeared mainly in the disc floret corolla elongation stage (Fig. 3). The cell counting revealed the difference in the morphology of the disc floret was caused by an increase in cell number and length. Anatomical observation of the two types of florets revealed that the adaxial cells of NAT disc floret were degraded, and only the adaxial cells were still somewhat vigorous, in contrast, the inner mid-layer cells of AT disc floret did not appear to be degraded, and the petal cells were multilayered with small rotundate cells and vigorous division activity (Fig. 4). In *Helianthus annuus’* mutant line, the ray florets were mutated to tubular-like ray florets. In cross sections, cell constrictions and smaller vascular bundles were detected in tubular-like ray florets rather than wild-type ray florets [45], suggesting it requires more cells and prominent vascular bundle tissue to form a distinct petal structure. It is speculated that the number of cells is increasing between the NAT disc floret and AT disc floret, and the cells are also different in functional differentiation. Two mutant lines ‘Meteor 1’ and ‘Meteor 2’ were found in another species of *Helianthus* × *multiflorus* cv. ‘Meteor’*.* The disc florets of ‘Meteor 2’ were markedly more elongated than those of ‘Meteor 1’, with significant corolla length and width differences. Longitudinal sections of both disc florets revealed that ‘Meteor 1’ consisted of only two layers of cells, while the disc florets of ‘Meteor 2’ consisted of multiple layers of cells [26]. The results we observed in AT disc floret were consistent with those studied in the sunflower. In our study, observing the sections of stage 1, stage 3, and stage 5 of the AT disc floret, it can be found that the number of middle cell layers decreases as the florets develop. It is speculated that the cells proliferated rapidly at the early stage of petal development, while the cells began to elongate at the later stage, thus promoting the elongated growth of petals. As a result, the formation of AT disc floret relies on cell proliferation and expansion at the later stage of petal development. The continuous development and differentiation of the mid-layer cells of disc floret petals ensure the formation of the anemone flower type. The more cells in the middle layer and the stronger the division ability, the more typical the anemone type of chrysanthemum shows.

### MADS-box genes are involved in the morphogenesis of AT disc floret

MADS-box gene family is one of the important members of the regulatory network of genes that determine the identity of floral organs. In Arabidopsis, MADS-box transcription factors SVP and AGL24 determine the identity of floral organs after the emergence of floral primordia [46]. *SVP* genes from multiple species can maintain vegetative shoot identity, delaying blooming and affecting flower development [47–49]. Overexpression of chrysanthemum’s *CmSVP* gene regulated inflorescence architecture in transgenic Arabidopsis [50]. The SVP transcription factor obtained from this study was barely expressed in AT disc floret. Overexpression of *MtSVP1* in *Medicago* results in longer pedicels and smaller and abnormal flowers [49]. In *Chrysanthemum* × *morifolium*, the structure of ray floret and AT disc floret is similar, we hypothesized that *SVP* is likely to play a role in determining the petal formation, thus affecting the phenotype of AT disc floret. Moreover, SVP can bind to transcription factors such as AP1 to regulate the expression of B- and C- class genes, thereby affecting flower organ development [51]. We also screened for B-class *GDEF1* and C-class *AG*, which are lowly expressed in AT disc floret. SVP might inhibit their expression, thus affecting petal development.

In the ABC model, the B-class MADS-box gene controls floral organ identity in whorls 2 and 3. *GDEF1* expresses in disc florets rather than ray florets and function of *GDEF1* is partially redundant with the other gerbera B-class genes in Gerbera, [52]. In AT chrysanthemum, we found *GDEF1* expressed in AT disc floret lower than NAT disc floret. While AT is disc flower identity but has a similar phenotype to ray florets, we speculated that low expression of *GDEF1* in AT disc florets and high expression in NAT disc florets might form the AT phenotype. Additionally, in another Gerbera transgenic line, the trans flower length was significantly shortened by silencing the expression of *GDEF3*. As the function of *GDEF1* is partially redundant with the other gerbera B-class genes [52], the *GDEF1* in AT flower may have a different function against *GDEF1* in gerbera.

C-class MADS-box genes control whorl 3 and 4 of floral organogenesis. *AG*1 belongs to C-class genes in the MADS-box family which were also expressed in petals. In the present study, *AG1* was highly expressed in NAT disc floret and lowly expressed in AT disc floret and ray floret. In Gerbera, ectopic expression of *GAGA1* (*AG1* homologous gene) leads to the absence of petal structure, and suppressing the expression of *GAGA1* appears a petal structure [53]. It is a remarkable fact that *AG1* was highly expressed at stage 4 and lowly expressed at stage 5 and stage 6 of AT disc floret, suggesting that low expression of *AG1* at later stages might affect cell number or size, thus elongating the petal. One example is in *Phalaenopsis*, the C-class *AGAMOUS-*like gene *PeMADS1* may also regulate floral organ size primarily by affecting cell size, the adaxial epidermal cells in the petal in *PeMADS1*-silenced flowers were smaller, respectively, than those of mock-treated flowers [54]. In addition, the stamen in AT disc florets is partially fertile as in NAT disc florets are fertile (Additional file 14: Fig. S10). In chrysanthemum, transgenic downregulation of the *AG1* gene led to the conversion of both stamen and pistil into corolla-like tissue, suggesting a critical effect of *AG1* in petal formation [55], suggesting *AG1* is close to stamen identity. We thought that *AG1* might affect cell size to regulate the AT disc florets, the function of *AG1* in AT flower still needs to be further discovered.

### *CYC2d* promotes the development of AT disc floret

During flower development, *CYC2*-like genes have an early function in flower type designation and play an important role in regulating petal homogeneity and elongation [19–24]. In the present study, *CYC2d* was highly expressed in ray floret and AT disc floret and lowly expressed in NAT disc floret, with the highest expression in ray floret. Investigation of the transcript levels of *CmCYC2* genes in different tissues of chrysanthemum found that they were mainly expressed in ray florets, almost none in the disc florets, indicating that *CYC2* genes are closely associated with the formation of petal structure [24]. The AT disc floret possesses obvious petal structures compared with NAT disc floret, so we speculated that *CYC2d* promotes the formation of petal structures. In *C. lavandulifolium*, *ClCYC2d* is a repressor of ray flower development, overexpression of *ClCYC2d* decreases ray flower length and capitulum diameter [56]. In *S. vulgaris*, *SvRAY1* is mainly expressed in ray florets. After overexpression of *SvRAY1* (*CYC2d* homolog) in the capitulum, the ray floret petal of *SvRAY1* transgenic lines were shorter and significantly wider than wild-type [20]. In AT chrysanthemum, we found that AT disc floret indicated a significantly higher number of cells than NAT disc floret. The surface cells of AT disc floret were multilayered with obvious cell division activities, speculating that *CYC2d* may have formed AT disc floret by promoting cell division and cell differentiation. The above results suggested that the function of *CYC2* genes in controlling the proliferation and expansion of petal cells varies from gene to gene and from species to species. Previous studies in *Antirrhinum* spp. suggested that the maintenance of *CYC2* genes expression during late petal development depends on the B-class MADS-box gene *DEFICIENS* [57]. The *GDEF1* gene we found was expressed at a low level in AT disc floret, whereas *CYC2d* was expressed at a high level in AT disc floret, and it is speculated that the low expression of B-class genes may cause the elongation of AT disc floret at a later stage. Current studies in Asteraceae have shown that *CYC2* genes and MADS-box genes co-regulate petal elongation. In *G. hybrida*, E-class gene *GhGRCD5* can activate the expression of *GhCYC3,* and the petal length of *GhCYC3* (*CYC2d* homolog) overexpression lines had significantly increased in both trans and disc flowers with morphology similar to that of ray flowers [22]. Thus, we hypothesized that there might be a regulatory relationship between *CYC2d, GDEF1,* and *AG1* in AT chrysanthemum to jointly regulate the formation of AT disc floret.

### Phytohormone signaling is involved in the formation of AT disc floret

Plant hormones are indispensable in the process of plant growth and development [58]. As a signal for the initiation of flower development, auxin control petal shape and size by influencing cell division in the early stage and cell expansion in the later stage [59]. RNA-Seq suggests that AT disc floret development involves numerous auxin pathway-related genes at the transcriptional level, and we identified members of five classes: ARF, AUX/IAA, SAUR, GRAS, and GH3 (Fig. 6B), suggesting that the auxin pathway is essential for the formation of AT disc floret. In chrysanthemum, we found *ARF16,* which had very low expression in AT disc floret. ARF transcription factors are located downstream of the auxin signaling pathway, genetic analyses have shown that the *ARF16* regulates leaf morphology [60]. In cotton, *GhARF16-1* modulates leaf cell morphology and leaf size by transcriptionally regulating the *GhKNOX2-1* gene. Moreover, in Arabidopsis, ARF8 is a negative regulator of petal growth by restricting cell division at early development stages and limiting cell expansion through interaction with BPEp at late development stages [32]. This work shows a distinct difference between NAT disc floret and AT disc floret in cell number and morphology. We postulated that *ARF16* might affect petal phenotype by regulating petal cell number and morphology.

Jasmonates (JAs) are essential for plant developmental process regulation. Genes related to JA biosynthesis and signaling have been extensively investigated in Arabidopsis (*Arabidopsis thaliana*), tomato (*Lycopersicon esculentum* L.), rice (*Oryza sativa* L.), maize (*Zea mays* L.), and other plants and the bHLH transcription factor MYC2 is a major regulator of the jasmonic acid signaling pathway [61, 62]. *MYC2* was expressed higher in AT disc floret and ray florets and lower in NAT disc floret. *MYC2* mainly regulates the interaction between jasmonic acid and other hormones such as abscisic acid (ABA), gibberellin (GA), and auxin (IAA) signals, and it is also involved in the JA signaling pathway to regulate plant development and the flowering process [63]. *OsMYC2*, a major regulator of JA signaling, activates *OsMADS1* expression and regulates rice spikelet development by binding to the promoter region [64]. Both MYC2 and MADS-box transcription factors were obtained in this study. It is hypothesized that MYC2 may form the AT phenotype by regulating the expression of MADS-box transcription factors. The current study found that the jasmonic acid signaling pathway and the auxin signaling pathway have striking mechanistic similarities, and ultimately both are involved in the same biological process [65]. Auxin response factors can regulate the expression of some genes on the jasmonic acid pathway. For instance, in Arabidopsis, *ARF6* and *ARF8* were found to activate the expression of *JAZ* on the jasmonic acid pathway to regulate the flowering process [30]. In chrysanthemum, genes involved in the auxin pathway and the jasmonic acid pathway may be linked to each other to jointly regulate AT disc floret formation.

## Conclusion

**Fig. 8:**
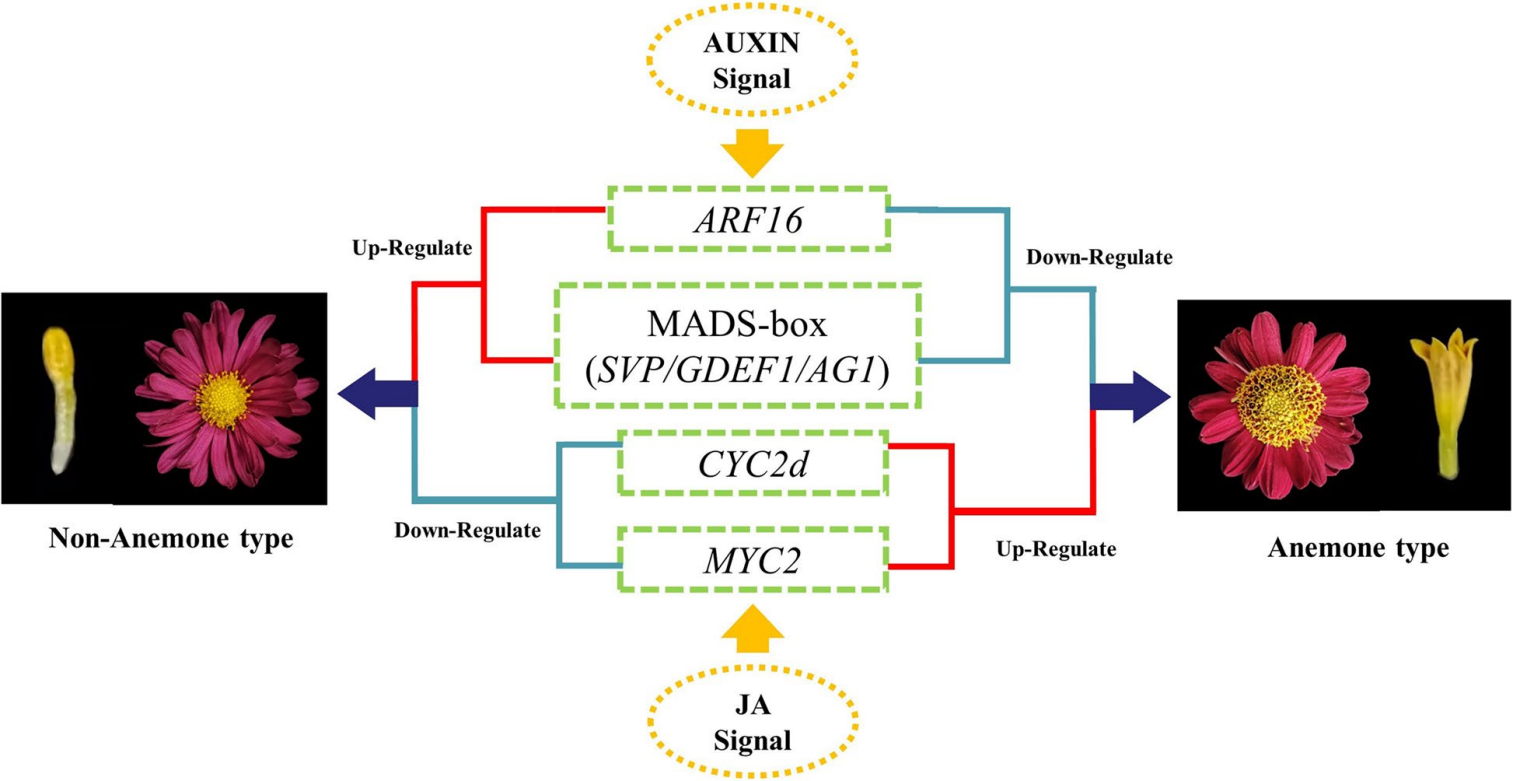
Summary of MADS-box, *CYC2d*, and plant hormone-related gene regulation of AT disc floret

This work presents the combined phenotype and transcript profiling analysis on NAT and AT disc florets development in *Chrysanthemum* × *morifolium*. A possible mechanism of AT disc floret growth has been provided. By observing the developmental process of NAT and AT disc floret, it was determined that morphological differences appear after the corolla formation stage of disc floret. The great amount and vigorous division of cells in the middle layer of the disc florets lead to the formation of AT chrysanthemum. Using transcriptomic analysis combined with gene expression analysis, it was found that *CYC2d* and *MYC2* were expressed more highly in AT disc floret than in NAT disc floret. Auxin signal-related gene *ARF16*, MADS-box genes *SVP*, *GDEF1*, and *AG1* were lowly expressed in AT disc floret. Based on these findings and combined with previous studies, a regulatory model of NAT disc floret and AT disc floret was proposed (Fig. 8). Auxin and jasmonic acid signaling influence the expression of *ARF16* and *MYC2*, with *MYC2* and *CYC2d* up-regulating, *ARF16* and MADS-box genes down-regulating, to form the AT disc floret phenotype. Capitulum development involves numerous genes, which is different from the regulatory pattern in single flowers, and future work will focus on the regulatory relationship among these genes. In conclusion, this study raises the prospects of the formation mechanism of AT disc floret in chrysanthemum, which lays a foundation for further study of the genetic regulatory mechanism and reveals a new strategy for the understanding of different morphological disc floret formation in chrysanthemum.

## Methods

### Plant materials and growth conditions

ALL chrysanthemums used in this study were cultivars preserved in long-term conservation at the chrysanthemum germplasm nursery of the Beijing Forestry University. All experiments adhered to the Regulations of the People’s Republic of China for Wild Plants Protection.

In this study, eight chrysanthemum cultivars were used as experimental materials, including three non-anemone-type (082, 086, PF) and five anemone-type (050, 068, 153, GS, FK) chrysanthemum cultivars (Fig. 2). The capitulum development of these cultivars were divided into six stages (Table. 1) based on our previous study [40]. The ray and disc florets of all six developmental stages were collected for SEM observation and paraffin section analysis (Additional file 1: Figure S1.).

### SEM observation

The apical buds of 082 and 068 at six stages (Table. 1) were fixed in 2.5% v/v glutaraldehyde for at least 12 h after removing the bracts. All materials were dehydrated by an ethanol series (30, 50, 70, 90, 95, 100%), followed by an ethanol-tert-butanol series up to 100% tert-butanol. The samples were freeze-dried overnight using a lyophilizer (ES-2030; Tokyo; HITACHI; Japan), dissected, and attached to carbon conductive tabs. The materials were coated with an ion sputtering apparatus (E-1010; Tokyo; HITACHI; Japan) and observed by SEM (S-3400 N II; Tokyo; HITACHI; Japan). To observe the cell morphology from upper to lower, the upper, middle, and lower parts of the disc florets were taken and examined under a magnified field of view of 800 × . The cell numbers were counted using Image J.

### Histological analysis

Disc florets of six development stages (Table. 1) of 082 and 068 were collected and fixed for 24 h in FAA solution [5% (v/v) acetic acid, 50% (v/v) ethanol, 10% (v/v) formaldehyde, and 35% distilled water]. Put the dehydration box into the dehydrator to dehydrate with gradient alcohol. 75% alcohol 4 h,85% alcohol 2 h,90% alcohol 2 h,95% alcohol 1 h, anhydrous ethanol I 30 min, anhydrous ethanol II 30 min, alcohol benzene 5 ~ 10 min, xylene II 5 ~ 10 min,65℃ melting paraffin I 1 h,65 ℃ melting paraffin II 1 h,65℃ melting paraffin III 1 h for dehydration and wax leaching. The wax-soaked tissue is embedded in the embedding machine. Place the trimmed wax block cool at -20 °C freezing table, and slice the modified tissue chip wax block on the paraffin slicer for after observation. The specific method used has been described by Wen et al., 2019 [39].

### RNA sequencing, functional annotation, and data processing

RNA-Seq has been widely used in flower development studies. In our research, to identify the genes specifically expressed in anemone-type disc floret, ray florets and disc florets at stage 4-stage 6 of 082 and 068 were all selected for RNA-Seq analysis (Each stage of sample sequencing is repeated three times). In brief, total RNA was extracted from ray and disc florets of stage 4–6 and cDNA libraries were synthesized by Biomarker Technologies Corporation (Beijing, China). The obtained libraries were sequenced on the Illumina HiSeq 2500 platform (Illumina, USA). After adaptor sequences and low-quality sequences were removed, the clean reads were assembled into unigenes using SOAPdenovo software with the parameters -K29, − M2, − L50. The obtained unigene sequences were aligned using BLAST (E-value < 10^−5^) to the NR, Swiss-Prot, GO, COG, KOG, eggNOG, and KEGG databases and then annotated. The transcript abundances of all unigenes were estimated via the FPKM method using RSEM [66]. Analysis of all DEGs using Venn and cluster analyses.

### RT-PCR analysis

Semi-quantitative reverse transcription-polymerase chain reaction (RT-PCR) was performed to analyze the expression profiles of candidate genes from the RNA-Seq database. The total RNAs of ray and disc floret petal at stage 1–3 of two non-anemone (082, 086) and three anemone-type (050, 068, 153) chrysanthemum cultivars were extracted using a Plant RNA Rapid Extraction Kit (HUAYUEYANG Biotechnology, Beijing, China) and were used to synthesize cDNA for RT-PCR with the transcription kit. The procedure for semi-quantitative RT -PCR followed the method of a previous study by Lu et al., 2019 [67], which used *26S* as a reference gene and analyzed the genes closely related to petal morphogenesis. The primer sequences were shown in Additional file 15: Table S5.

### qRT-PCR validation

To verify the accuracy of the RT-PCR result, qRT-PCR reactions were performed using a CFX Connect Real-time System (Bio-Rad, USA) based on SYBR Premix Ex Taq (TaKaRa, Japan), according to the procedure described by Huang et al. [68]. The experimental materials used for qRT-PCR included ray florets and disc florets from three open stages of 3 non-anemone-type (Fig. 2. D-F) and 4 anemone-type (Fig. 2 G, H, J, K) chrysanthemum cultivars. The primer information is listed in Additional file 16: Table S6. Each qRT-PCR data point was derived from three biological and three technical replicates. The relative gene expression was normalized by comparison to the expression of *SAND* in *Chrysanthemum* × *morifolium* [69], and the analysis was performed using the 2^−ΔΔCT^ method [70]. The data are presented as the mean ± SD.

## Supplementary Information

**Additional file 1:** **Figure S1. **Six different development stages of non-anemone-type and anemone-type chrysanthemums based on the Table 1.

**Additional file 2:** **Table S1.** Summary statistics of clean reads in the transcriptomes of Chrysanthemum × morifolium.

**Additional file 3:** **Figure S2.** PCA analysis of the 36 samples.

**Additional file 4:** **Figure S3.** All genes annotation in public databases.

**Additional file 5:** **Figure S4.** GO terms (A-C) and KEGG pathways (D-F) significantly enriched in DEGs in comparisons of ND4_VS_AD4, ND5_VS_AD5, ND6_VS_AD6.

**Additional file 6:** **Table S2.** Pathyways involved in KEGG analysis

**Additional file 7:** **Figure S5.** Five of nine K-means cluster of DEGs.

**Additional file 8:** **Table S3.** Differential expression information and annotation of DETFs.

**Additional file 9:** **Table S4.** The DEGs encoding transcription factors related to plant hormone pathway.

**Additional file 10:** **Figure S6.** The original and full-length gel images of DEGs between NAT and AT disc floret.

**Additional file 11:** **Figure S7.** RT-PCR analysis of DEGs in two non-anemone-type (082 and 086) and three anemone-type (050, 068, and 153) chrysanthemums using RT-PCR.

**Additional file 12:** **Figure S8.** qRT-PCR analysis of 15 DEGs in three non-anemone-type (082, 086, and PF) and four anemone-type (050, 068, GS, FK) chrysanthemums.

**Additional file 13:** **Figure S9.** FPKM of 10 final DEGs in non-anemone-type (082) and anemone-type (068) chrysanthemums.

**Additional file 14:** **Figure S10.** The stamen of AT and NAT disc floret.

**Additional file 15:** **Table S5.** Primer sequences used in RT-PCR experiments.

**Additional file 16:** **Table S6.** Primer sequences used in qRT-PCR experiments.

## Data Availability

The datasets supporting the results presented in this study are included in this article (and its additional files). The raw data for the 36 sequenced libraries are available in the NCBI SRA database with the accession number PRJNA870232.

## Abbreviations

ABA: Abscisic acid
AG: Agamous
AP1: Apetala1
AP2/ERF: Apetala2/ethylene responsive factor
ARF: Auxin response factor
AUX/IAA: Auxin/indole3-acetic acid
AT: Anemone-type
bHLH: Basic Helix-Loop-Helix
BPEp: BIGPETALp
BR: Brassinolide
CDM: Chrysanthemum Dendrathema mads
COI: Coronatine insensitive
CYC: Cycloidea
DEGs: Differentially expressed genes
DETFs: Differentially expressed transcription factors
DOF: DNA-binding with One Finger
ET: Ethylene
FC: Fold change
FDR: False discovery rate
FM: Floral meristem
FPKM: Fragments per kilobase of transcript per million mapped reads
FUL: Fruitfull
GA: Gibberellin
GATA: GATA-motif binding factors
GAGA: Gerbera-agamous
GDEF: Gerbera deficiens-like
GH3: Gretchen hagen3
GRAS: GAI-RGA-and-SCR
GRCD: Gerbera regulator of capitulum development
IAA: Indoleacetic acid
JA: Jasmonic acid
JAs: Jasmonates
JAR: Jasmonate-resistant
JAZ: Jasmonate zim-domain
KNOX: KNOTTED1-like homeobox
MADS: MCM1, AG, DEFA and SRF
MAPK: Mitogen-activated protein kinase
ML: Mesophyll layer cells
MYB: MYB family transcription factor
MYC: Myelocytomatosis
NAC: NAM/ATAF/CUC
NAT: Non-anemone-type
qRT-PCR: Real-time quantitative polymerase chainreaction
QTL: Quantitative trait locus
RNA-Seq: RNA Squencing
RT-PCR: Semi-quantitative reverse transcriptase-polymerase chain reaction
SAND: Sp100, AIRE-1, NucP41/75 and DEAF-1
SAUR: Small auxin upregulated RNA
SEM: Scanning electron microscopy
SOC: Suppressor of overexpression of constans
SVP: Short vegetative phase
TCP: Teosinte Branched/Cycloidea/PCF
TF: Transcription factor
TE: Transposable element
UE: Upper epidermal cells
WIP: Wound-induced protein
WRKY: WRKY domain-containing protein
WOX: WUSCHEL-like homeobox
