# Supplementary material for: Transcriptomic analysis reveals the formation mechanism of anemone-type flower in chrysanthemum
Source: BMC Genomics. 2022 Dec 22;23:846. doi: 10.1186/s12864-022-09078-3 (PMC9773529; doi:10.1186/s12864-022-09078-3)
Supplement: Supplementary file 1 — Additional file 1: Figure S1. Six different development stages of non-anemone-type and anemone-type chrysanthemums based on the Table 1. [file 12864_2022_9078_MOESM1_ESM.doc]

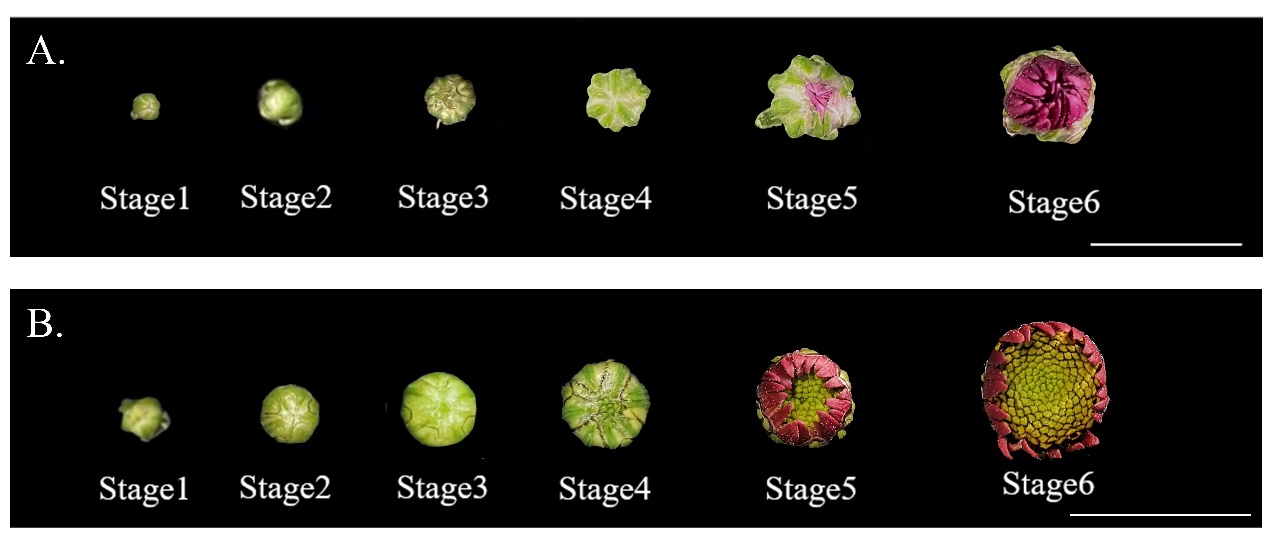


**Additional file 1: Figure S1.** Six different development stage of non-anemone-type and anemone-type chrysanthemums based on the Table 1. A. six stage of non-anemone-type chrysanthemums. B. six stage of anemone-type chrysanthemums. Scale bar=1cm.
