# Supplementary material for: Transcriptomic analysis reveals the formation mechanism of anemone-type flower in chrysanthemum
Source: BMC Genomics. 2022 Dec 22;23:846. doi: 10.1186/s12864-022-09078-3 (PMC9773529; doi:10.1186/s12864-022-09078-3)
Supplement: Supplementary file 3 — Additional file 3: Figure S2. PCA analysis of the 36 samples. [file 12864_2022_9078_MOESM3_ESM.doc]

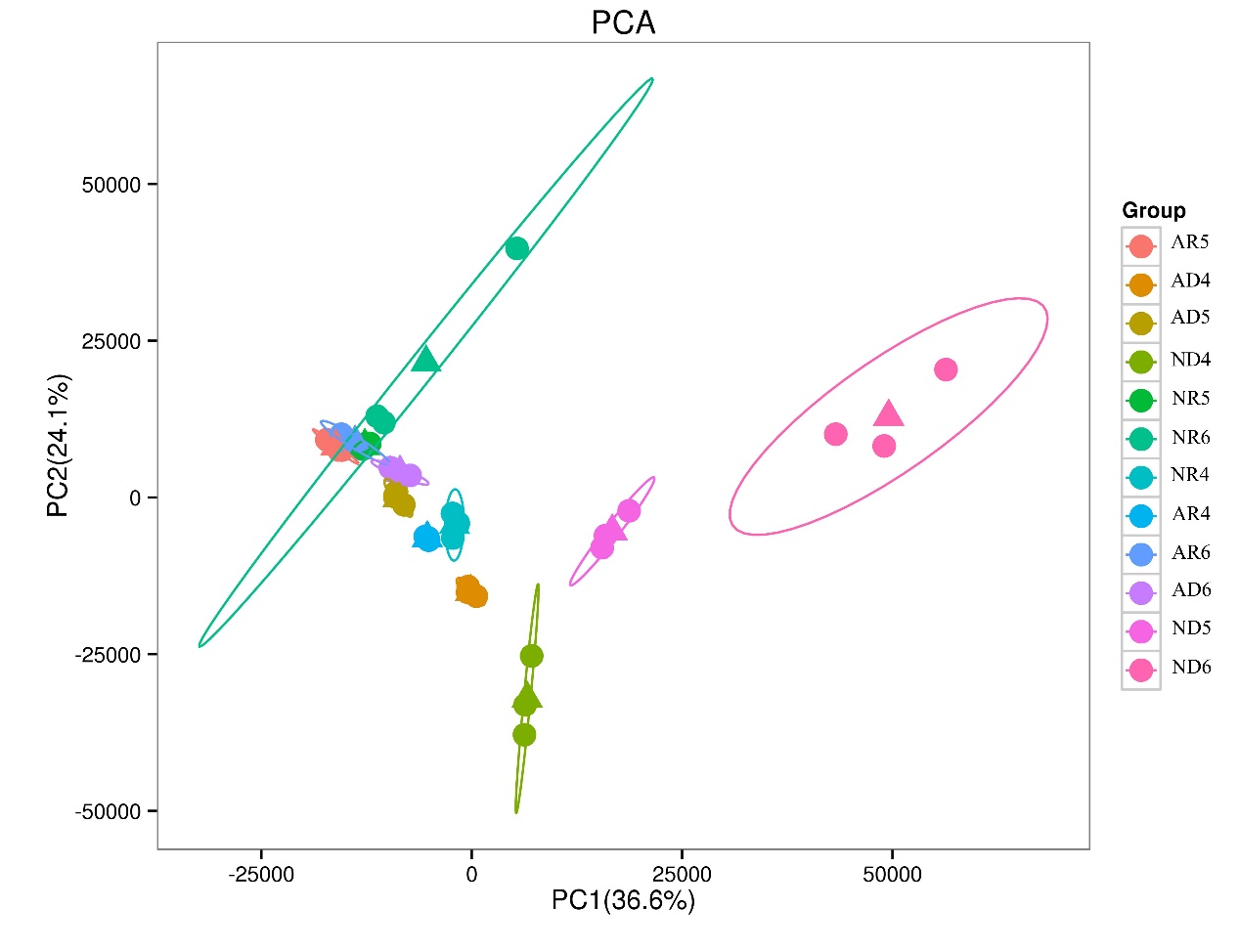


**Additional file 3: Figure S2.** PCA analysis of the 36 samples (NR4-1, NR4-2, NR4-3, AR4-1, AR4-2, AR4-3, NR5-1, NR5-2, NR5-3, AR5-1, AR5-2, AR5-3, NR6-1, NR6-2, NR6-3, AR6-1, AR6-2, AR6-3, ND4-1, ND4-2, ND4-3, AD4-1, AD4-2, AD4-3, ND5-1, ND5-2, ND5-3, AD5-1, AD5-2, AD5-3 ND6-1, ND6-2, ND6-3, AD6-1, AD6-2, AD6-3). Each group contains three biological replications.
