# Supplementary figures and images for: Transcriptomic analysis reveals the formation mechanism of anemone-type flower in chrysanthemum
Source: BMC Genomics. 2022 Dec 22;23:846. doi: 10.1186/s12864-022-09078-3 (PMC9773529; doi:10.1186/s12864-022-09078-3)

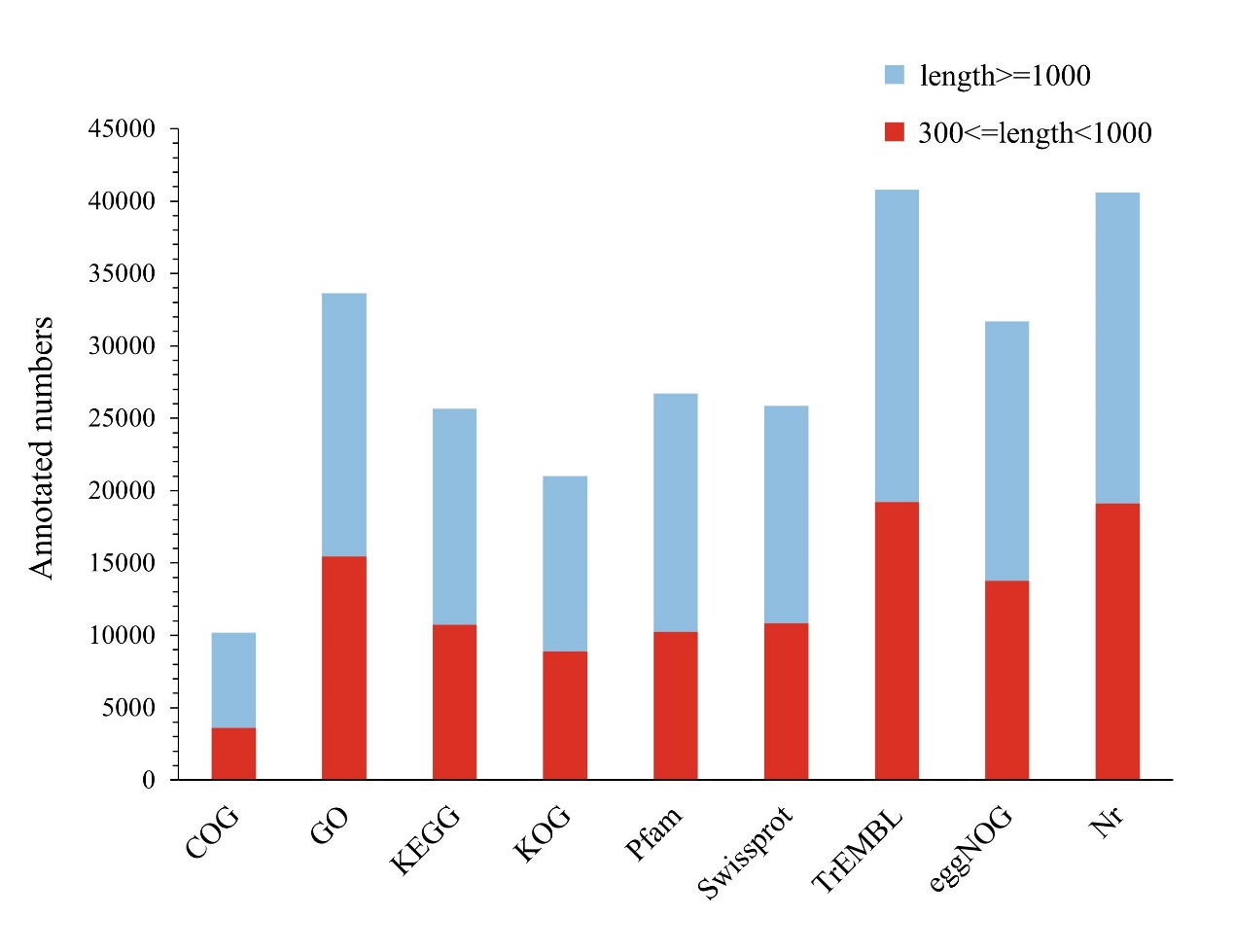


**Additional file 4: Figure S3.** All genes annotation in public databases.

Supplement: Supplementary file 4 — Additional file 4: Figure S3. All genes annotation in public databases. [file 12864_2022_9078_MOESM4_ESM.doc]

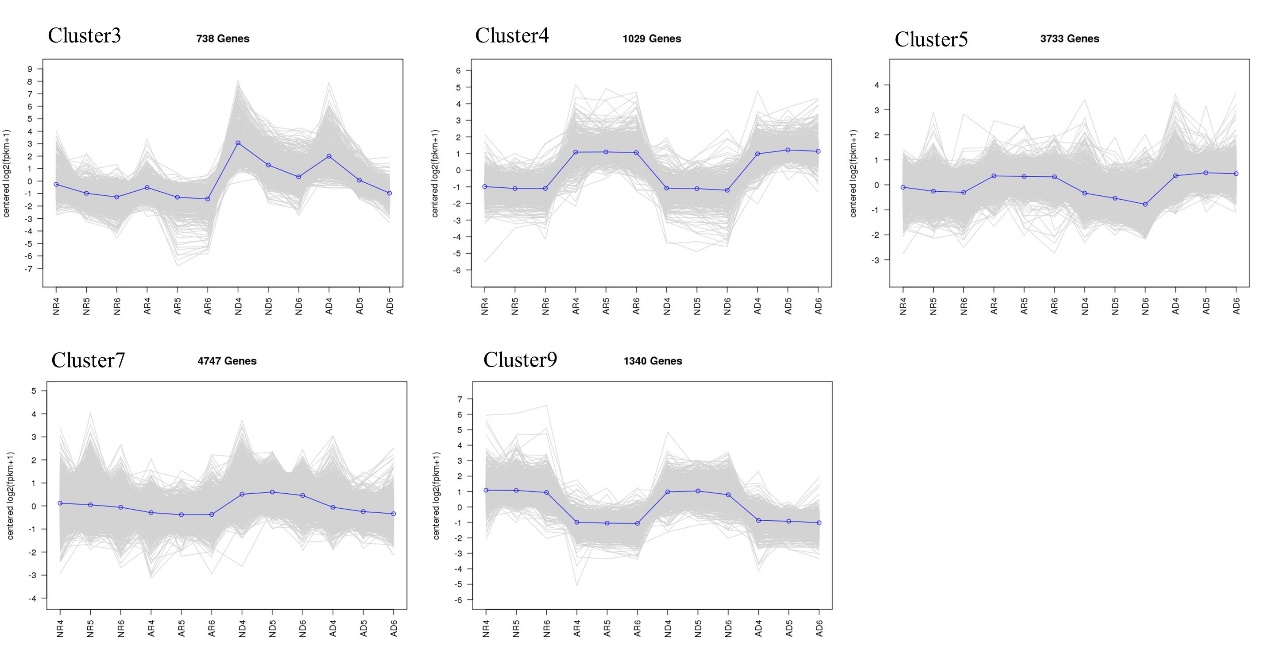


**Additional file 7: Figure S5.** Five of nine K-means cluster of DEGs.

Supplement: Supplementary file 7 — Additional file 7: Figure S5. Five of nine K-means cluster of DEGs. [file 12864_2022_9078_MOESM7_ESM.doc]
