# Supplementary material for: Transcriptomic analysis reveals the formation mechanism of anemone-type flower in chrysanthemum
Source: BMC Genomics. 2022 Dec 22;23:846. doi: 10.1186/s12864-022-09078-3 (PMC9773529; doi:10.1186/s12864-022-09078-3)
Supplement: Supplementary file 5 — Additional file 5: Figure S4. GO terms (A-C) and KEGG pathways (D-F) significantly enriched in DEGs in comparisons of ND4_VS_AD4, ND5_VS_AD5, ND6_VS_AD6. [file 12864_2022_9078_MOESM5_ESM.doc]

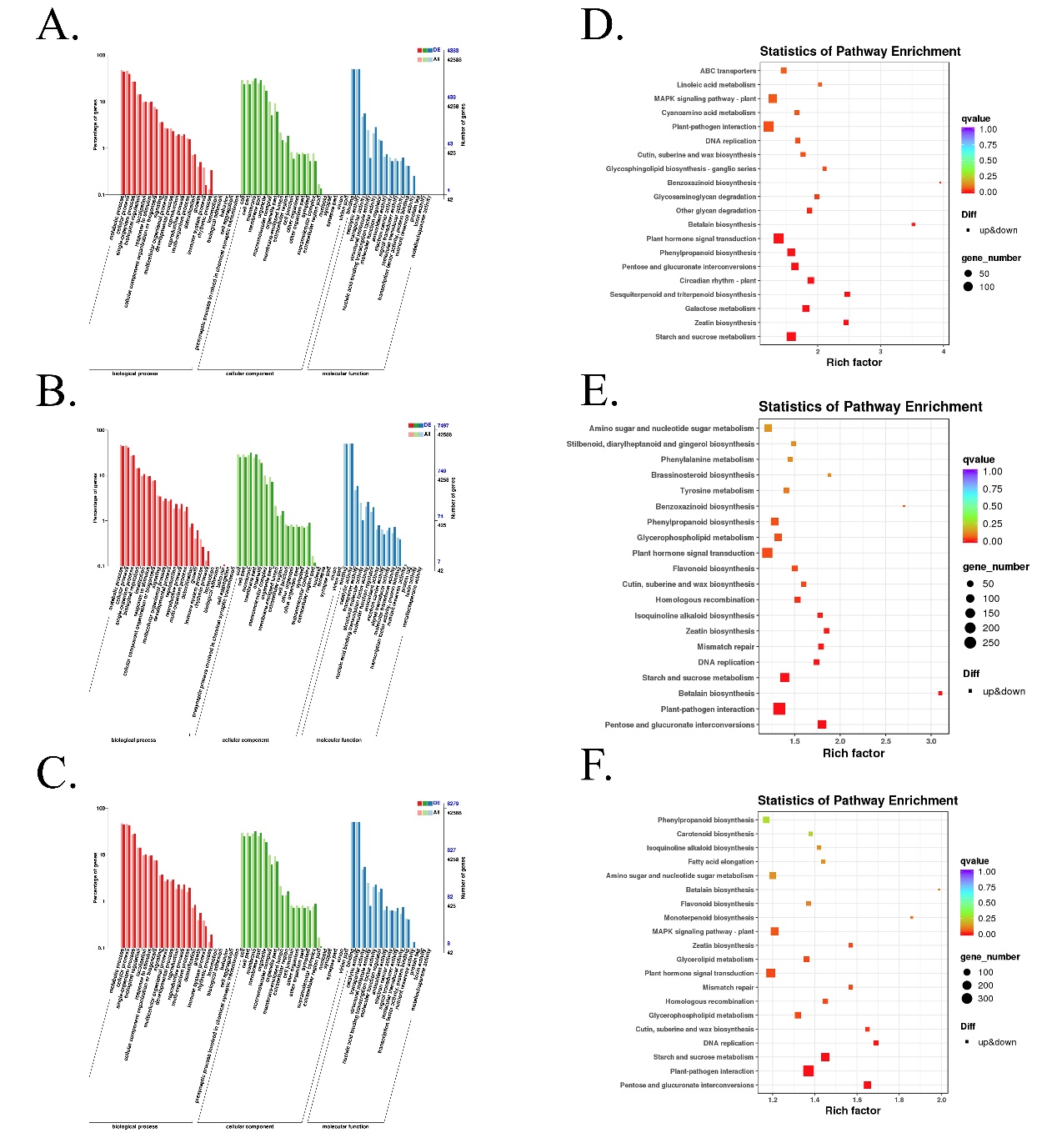


**Additional file 5: Figure S4.** GO terms (A-C) and KEGG pathways (D-F) significantly enriched in DEGs in comparisons of ND4_VS_AD4, ND5_VS_AD5, ND6_VS_AD6.
