# Supplementary material for: Transcriptomic analysis reveals the formation mechanism of anemone-type flower in chrysanthemum
Source: BMC Genomics. 2022 Dec 22;23:846. doi: 10.1186/s12864-022-09078-3 (PMC9773529; doi:10.1186/s12864-022-09078-3)
Supplement: Supplementary file 10 — Additional file 10: Figure S6. The original and full-length gel images of DEGs between NAT and AT disc floret. [file 12864_2022_9078_MOESM10_ESM.doc]

**Additional file 10: Figure S6.** The original and full-length gel images of DEGs between NAT and AT disc floret. R4-R6 indicated the three opening stages of ray floret, D4-D6 indicated the three opening stages of disc floret. NAT: non-anemone-type AT: anemone-type


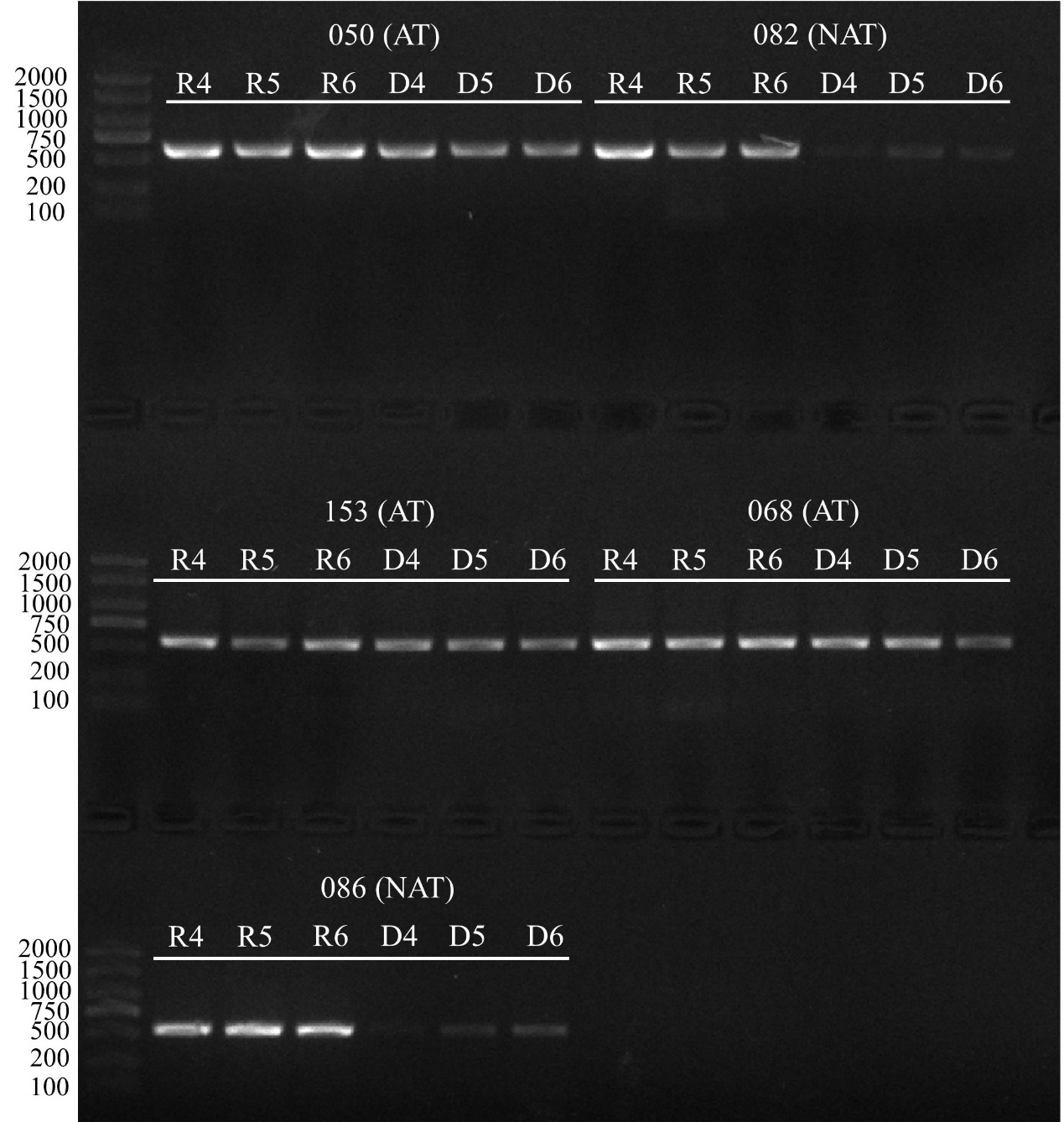


Figure S7-1. Expression analysis of *CYC2d*.


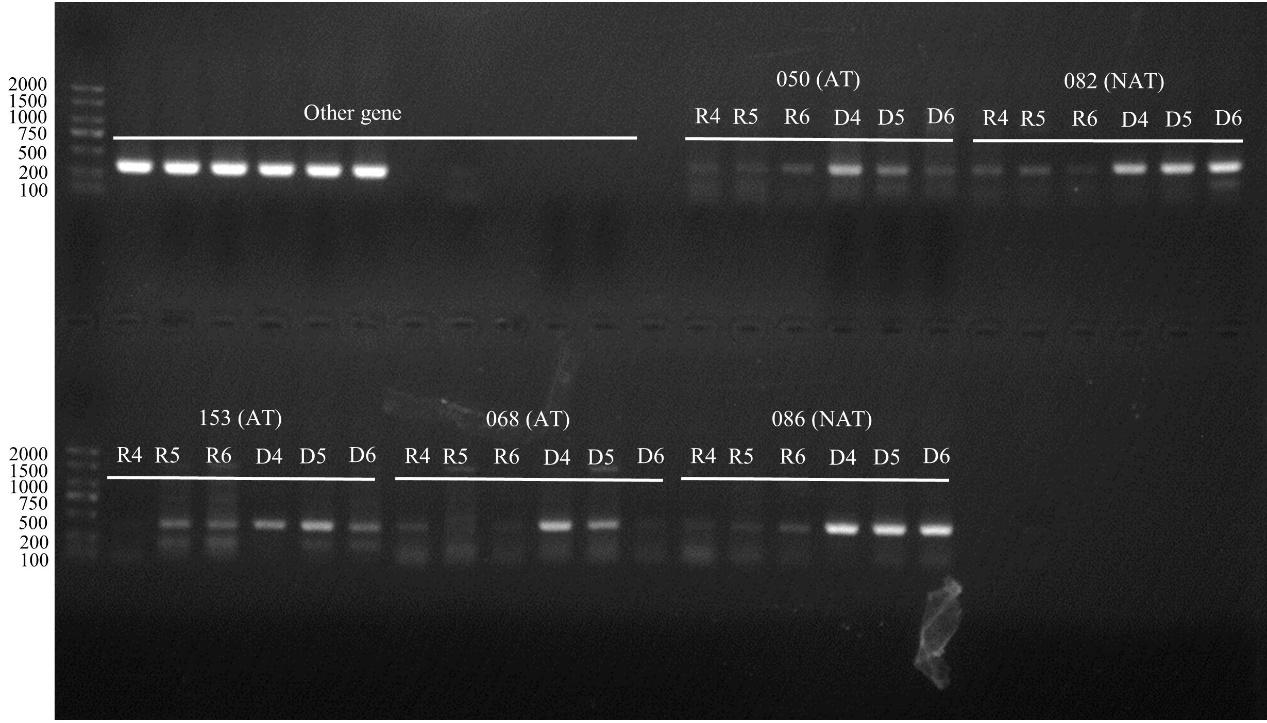


Figure S7-2. Expression analysis of *GDEF1.* Other genes refer to the materials unrelated to this study.


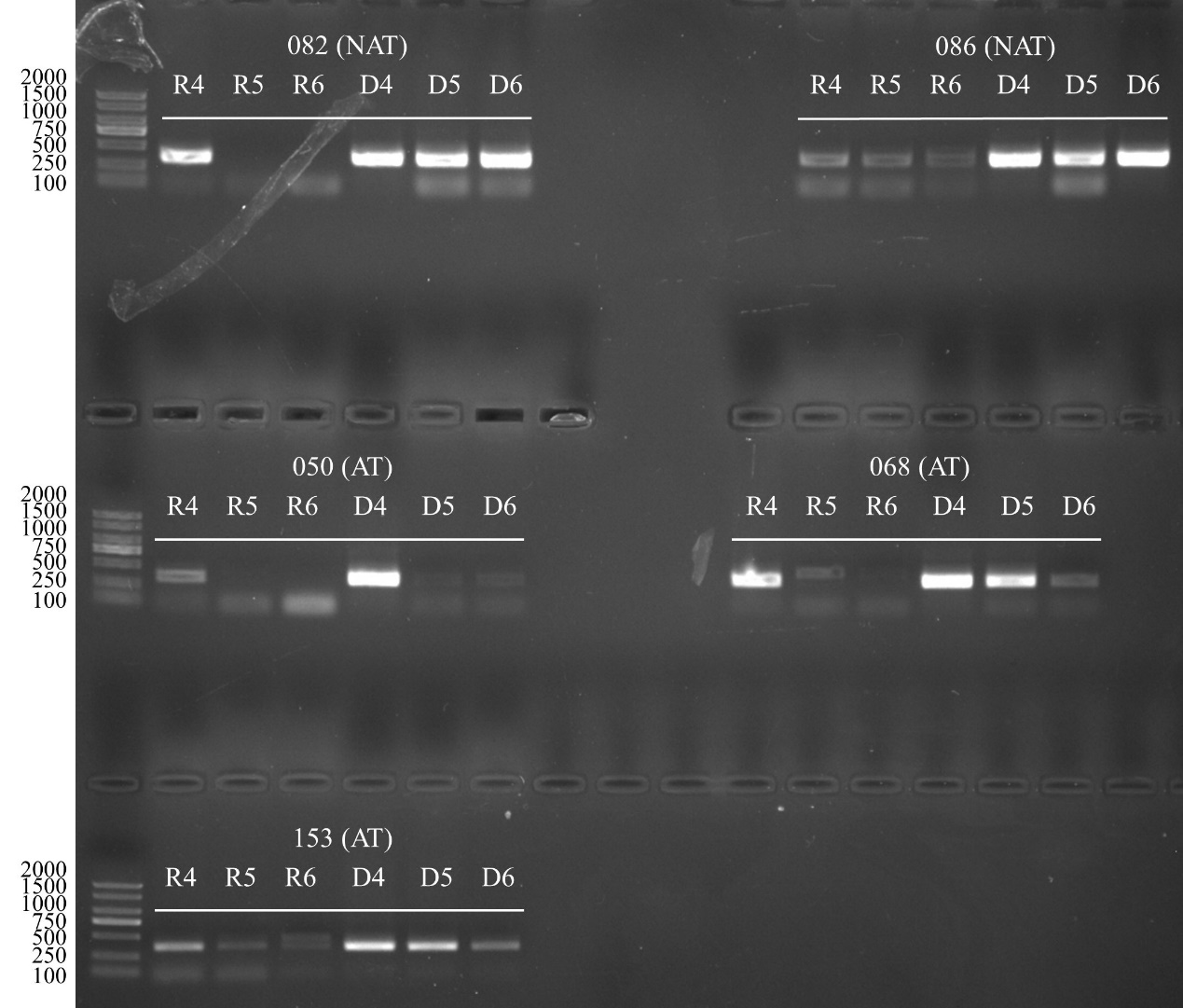


Figure S7-3. Expression analysis of *CAG1.*


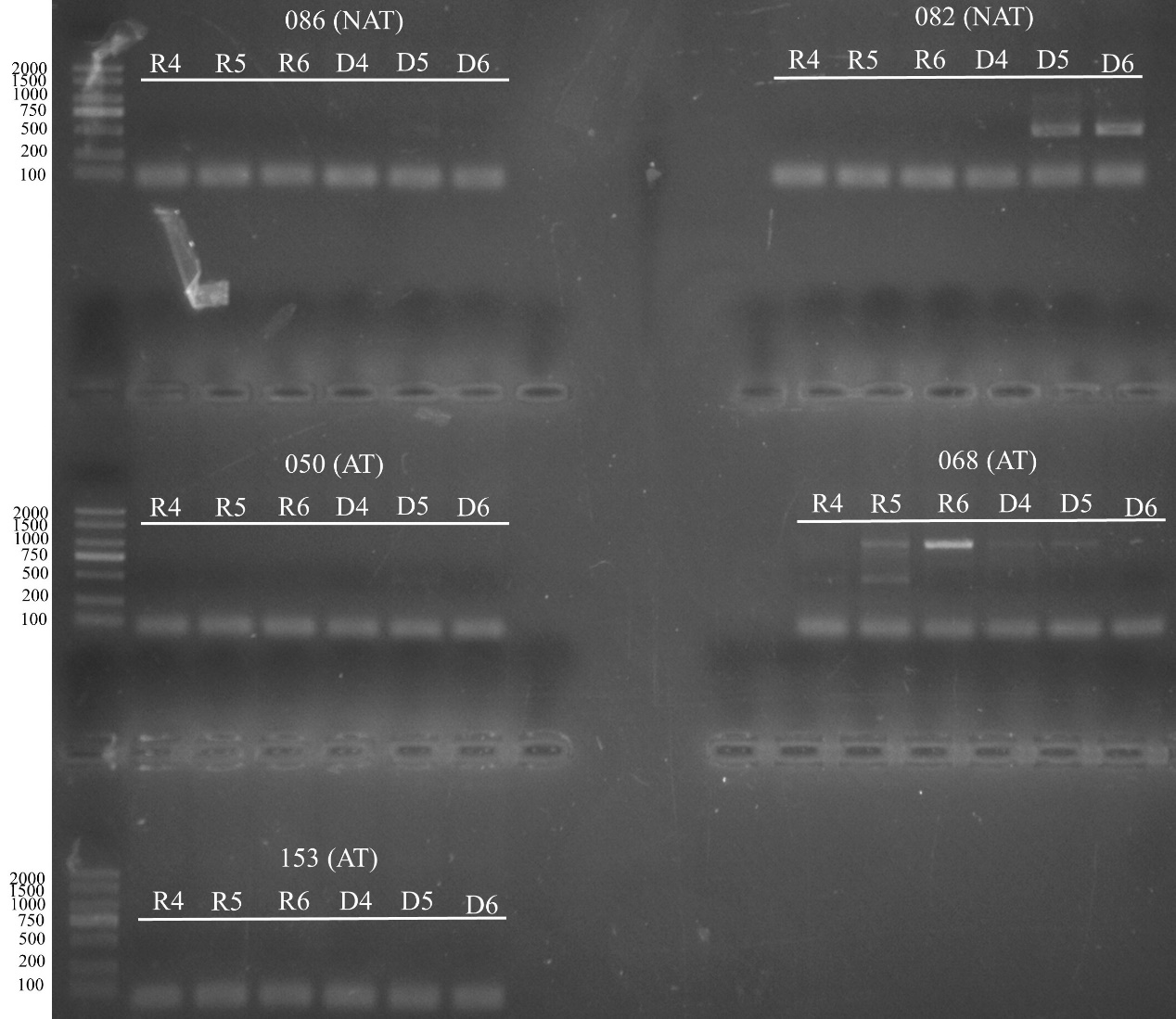


Figure S7-3. Expression analysis of *AGL104*

*
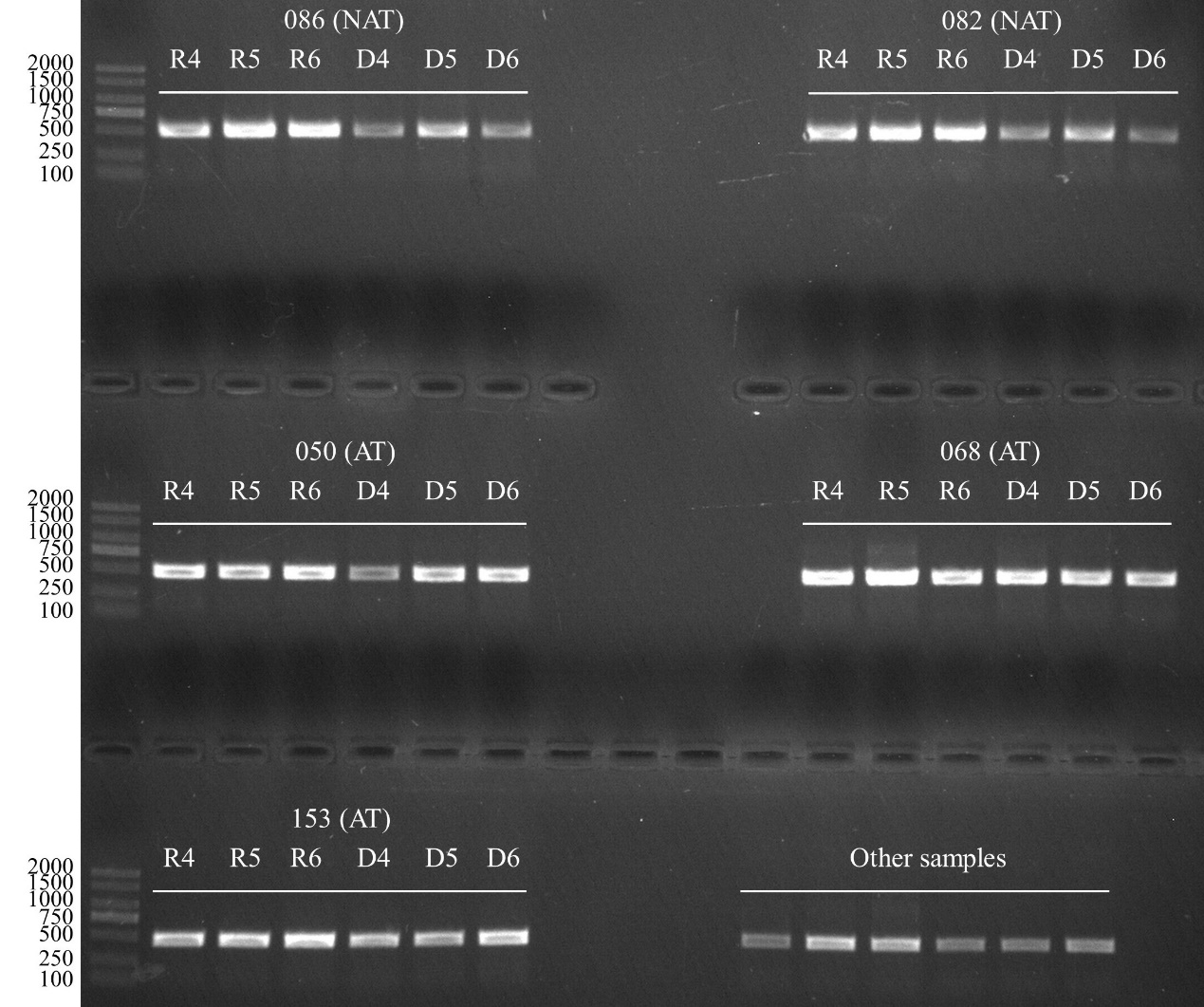
.* Figure S7-4. Expression analysis of *DOF2.*

*
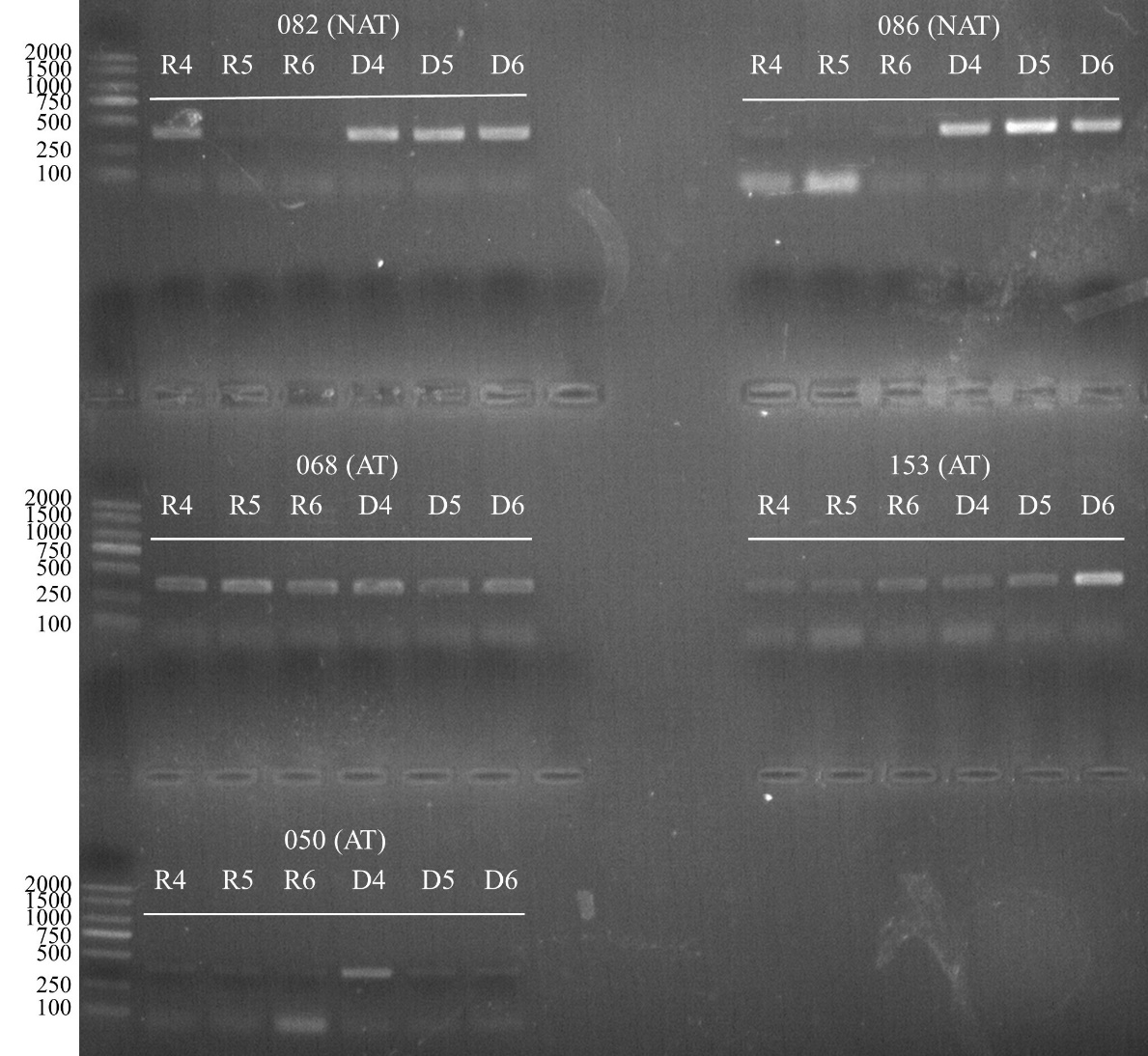
*

Figure S7-5. Expression analysis of *DOF12.*

*
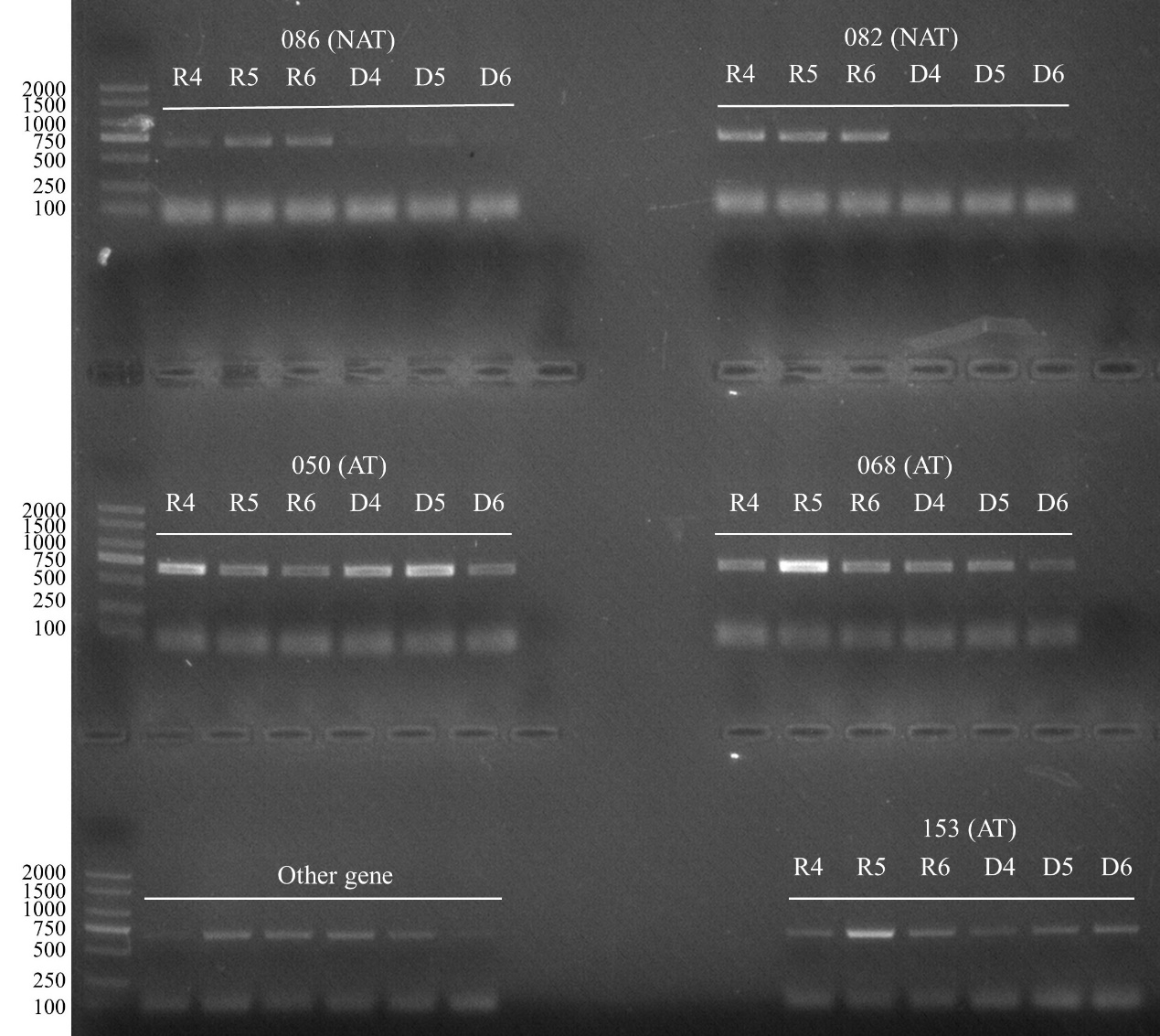
*

Figure S7-6. Expression analysis of *WOX8.*


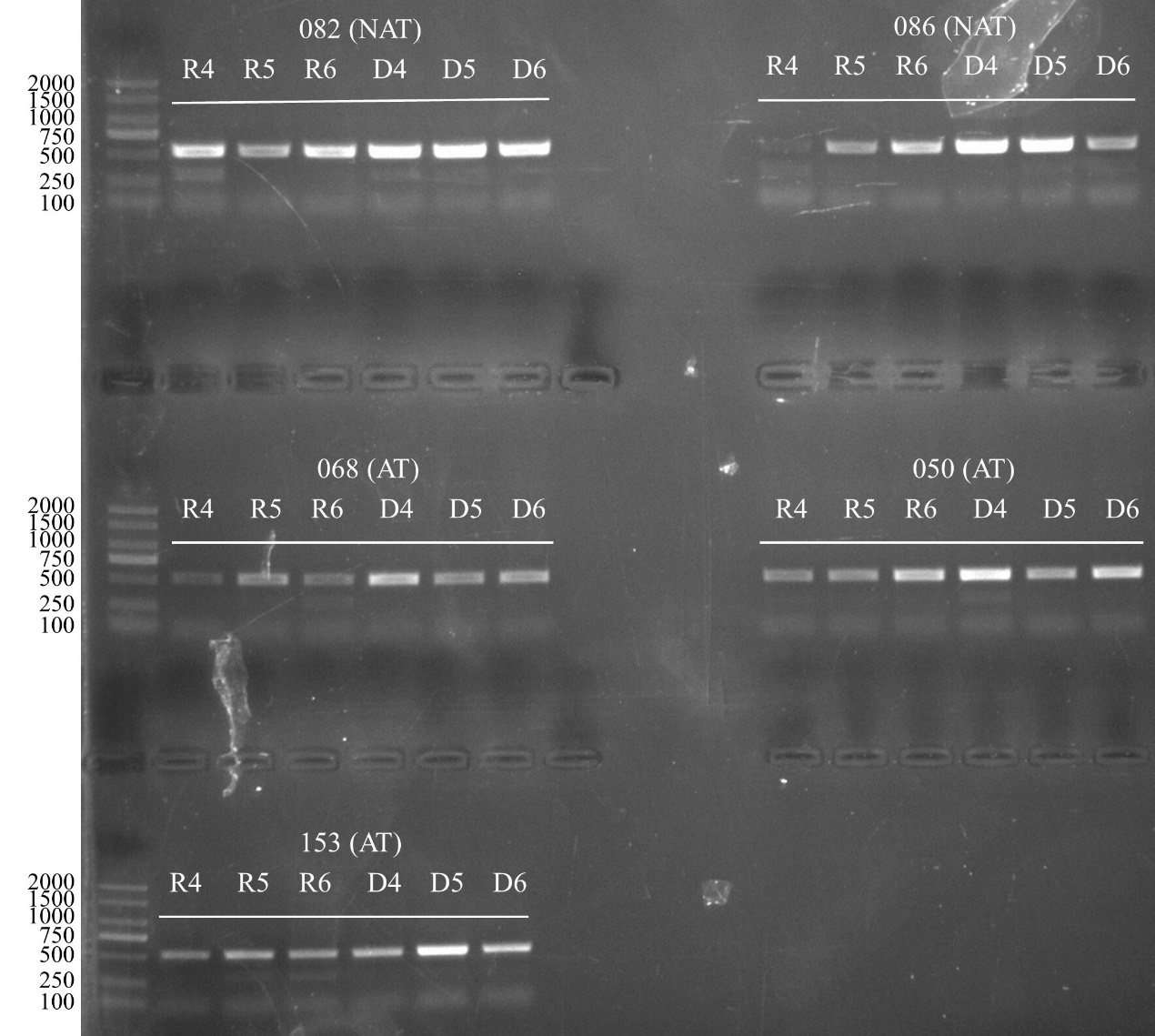


Figure S7-7. Expression analysis of *GAMYB.*

*
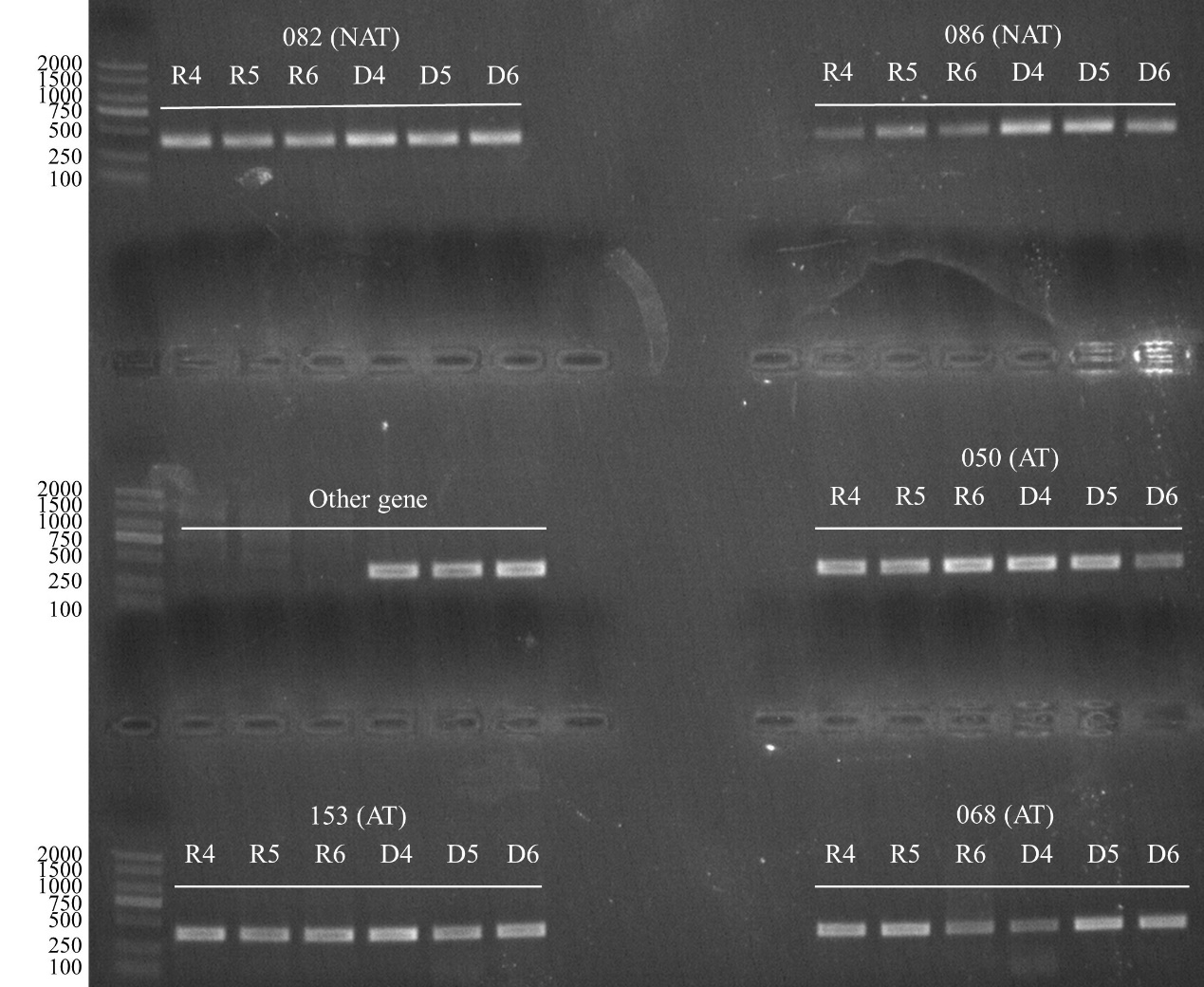
*

Figure S7-8. Expression analysis of *NAC83.*


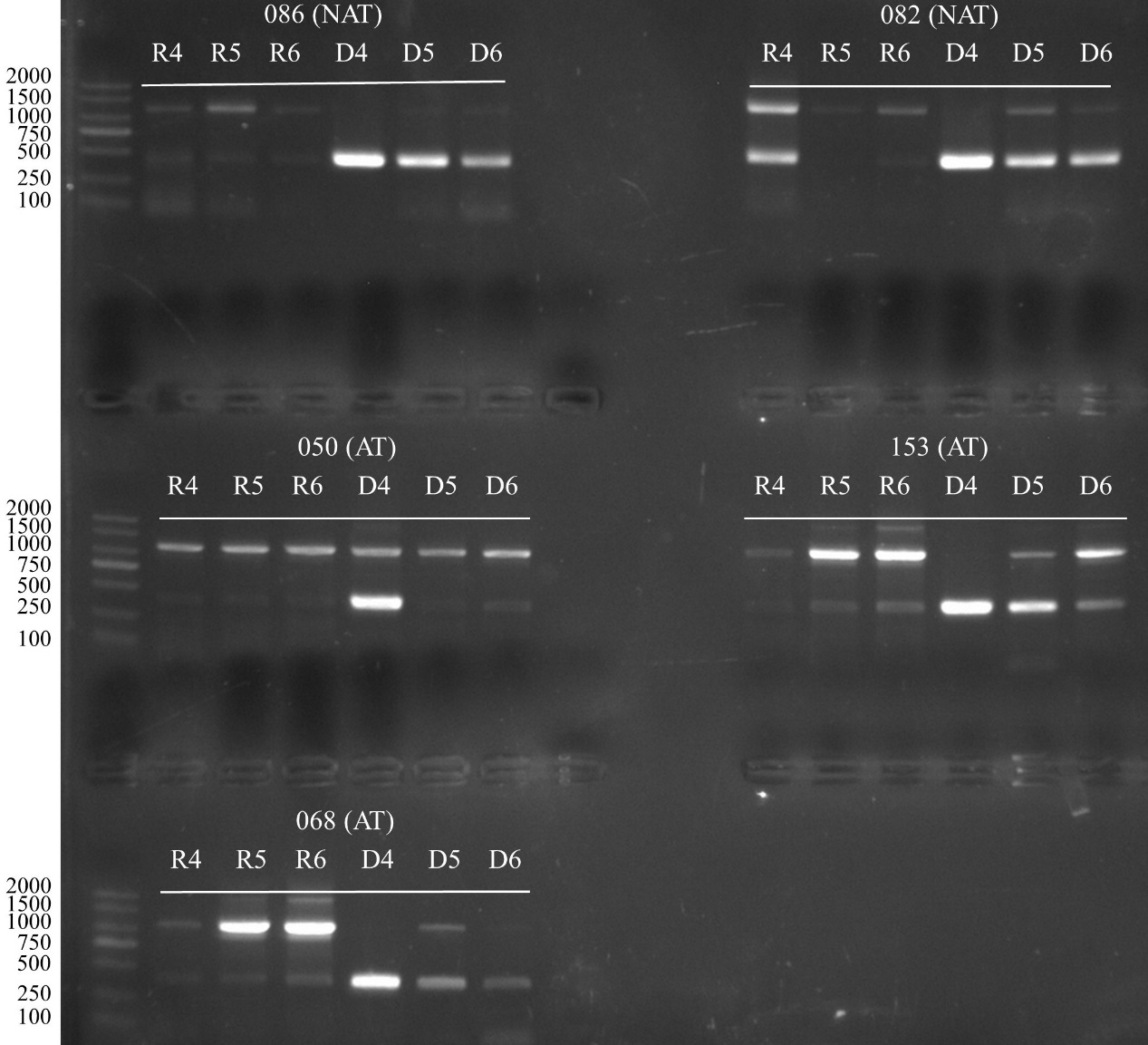


Figure S7-9. Expression analysis of *NAC56*

*
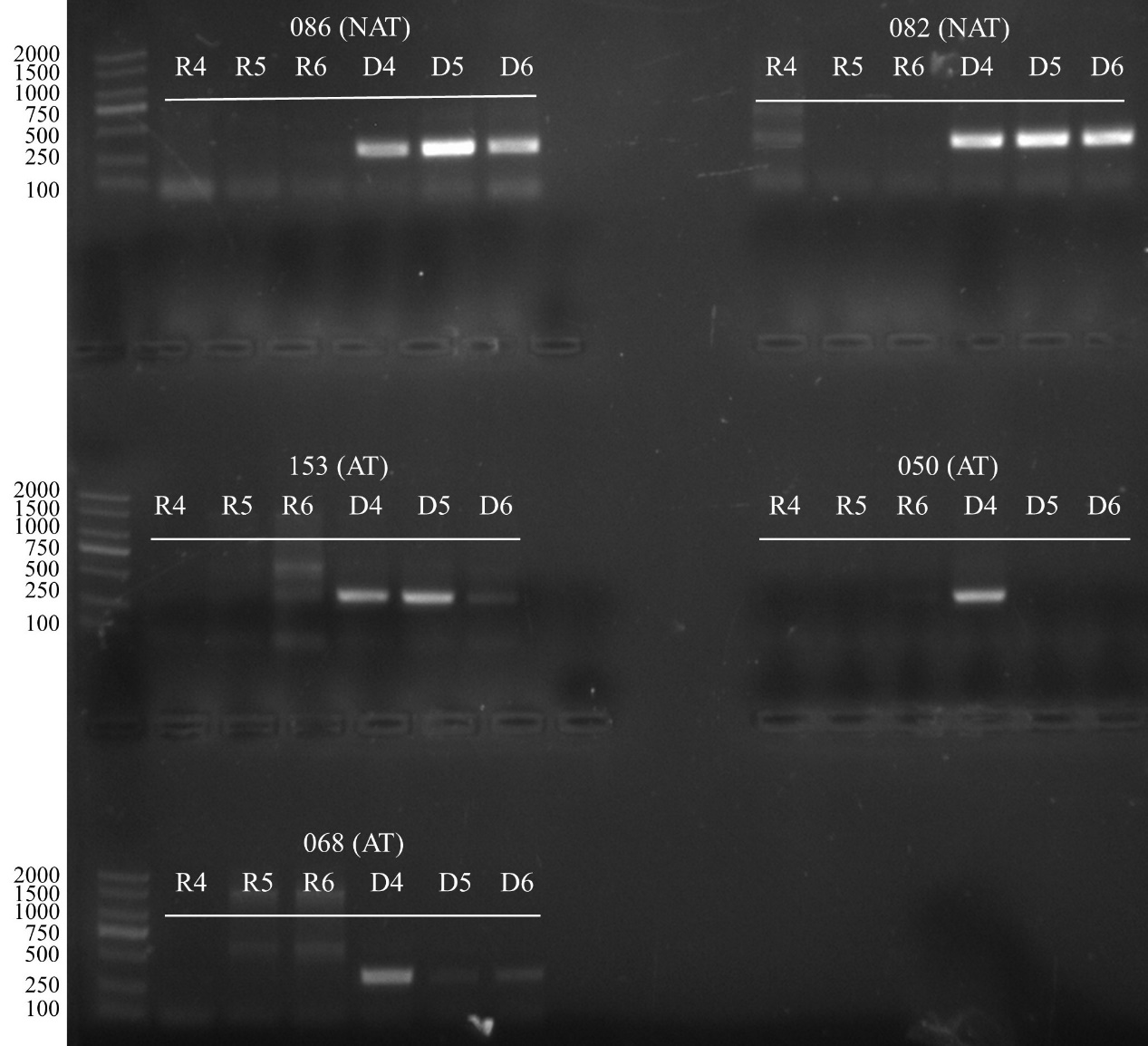
.* Figure S7-10. Expression analysis of *NAC25.*


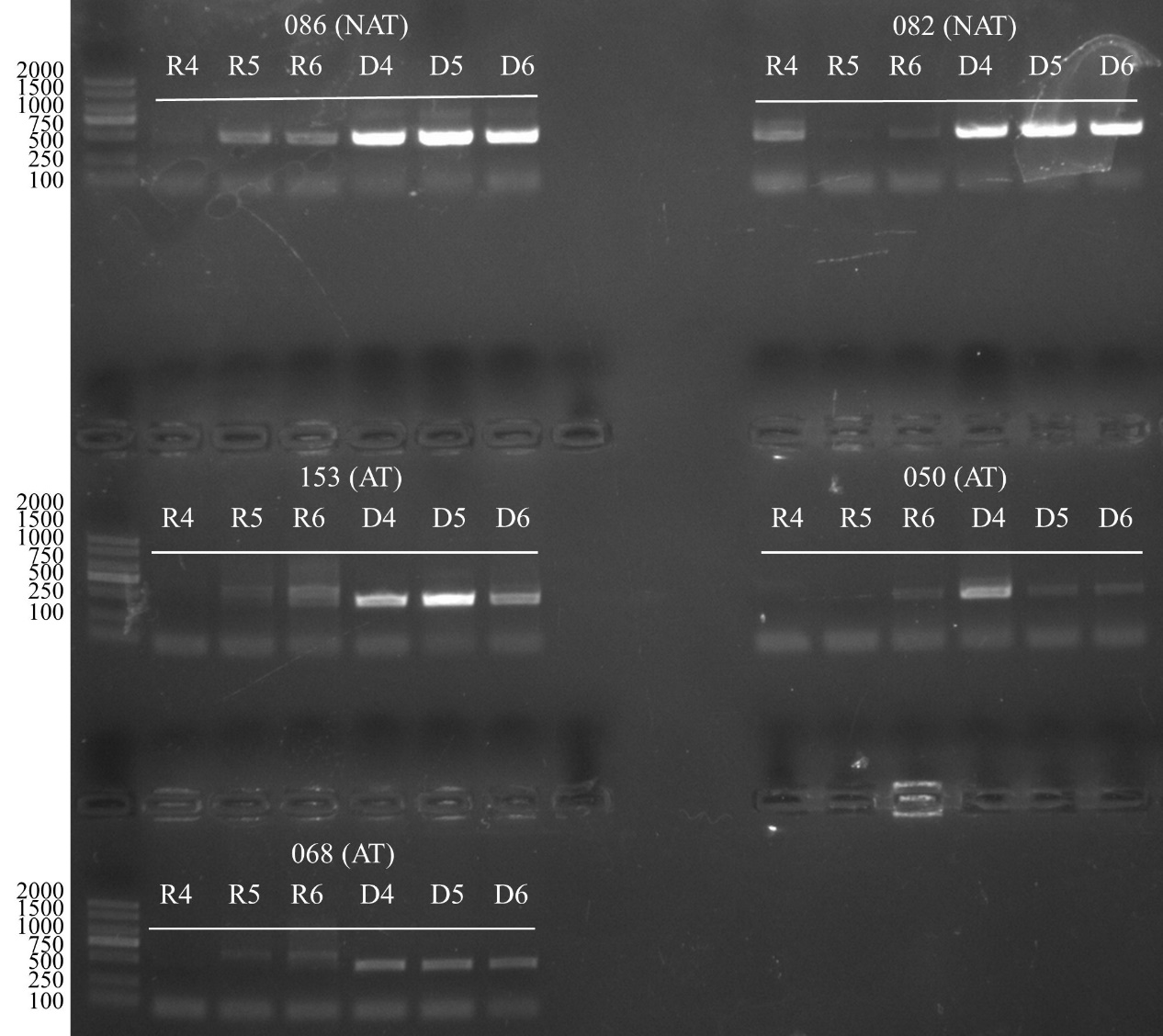


Figure S7-11. Expression analysis of *NAC100.*


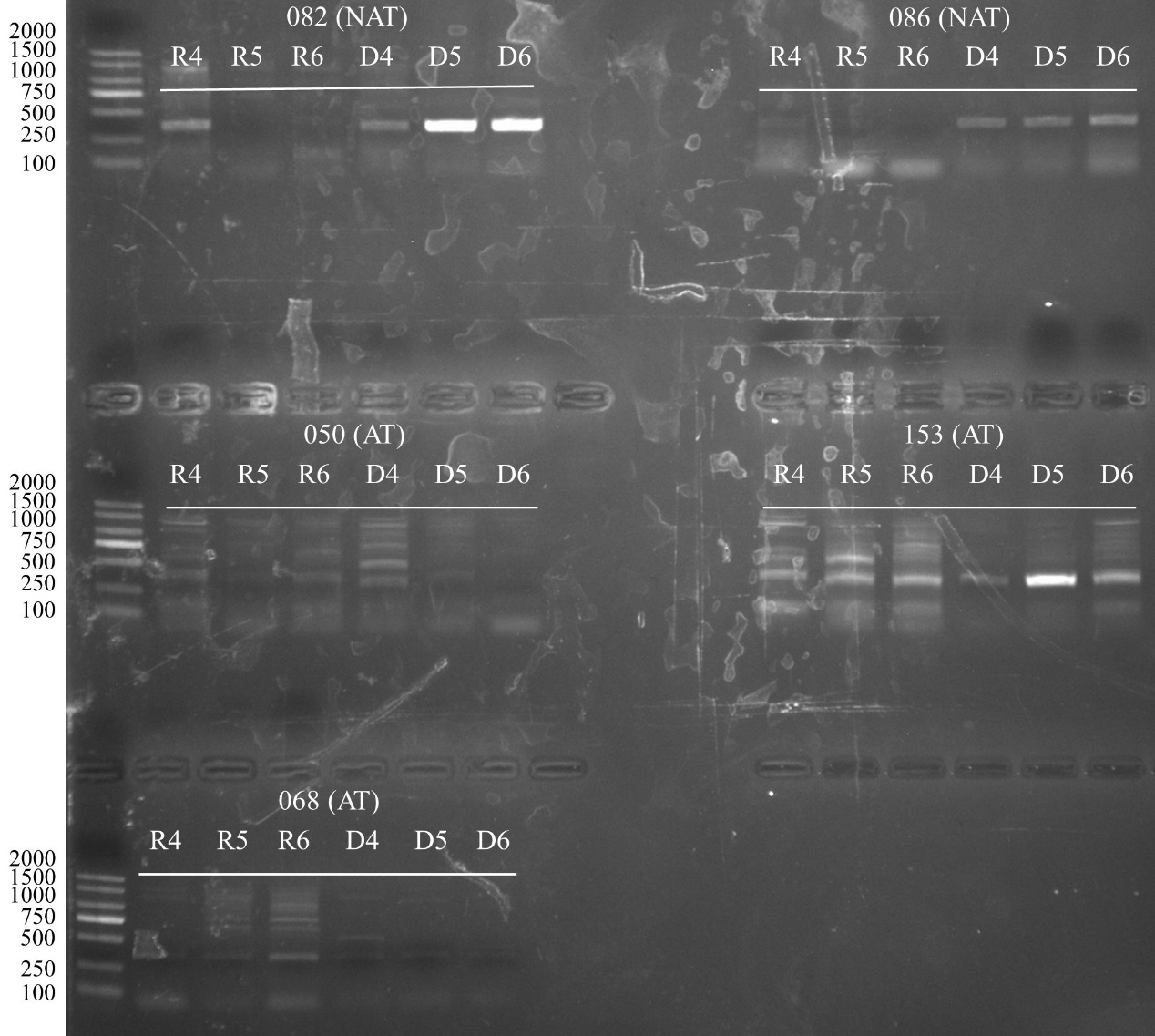


Figure S7-12. Expression analysis of *GATA12.*

*
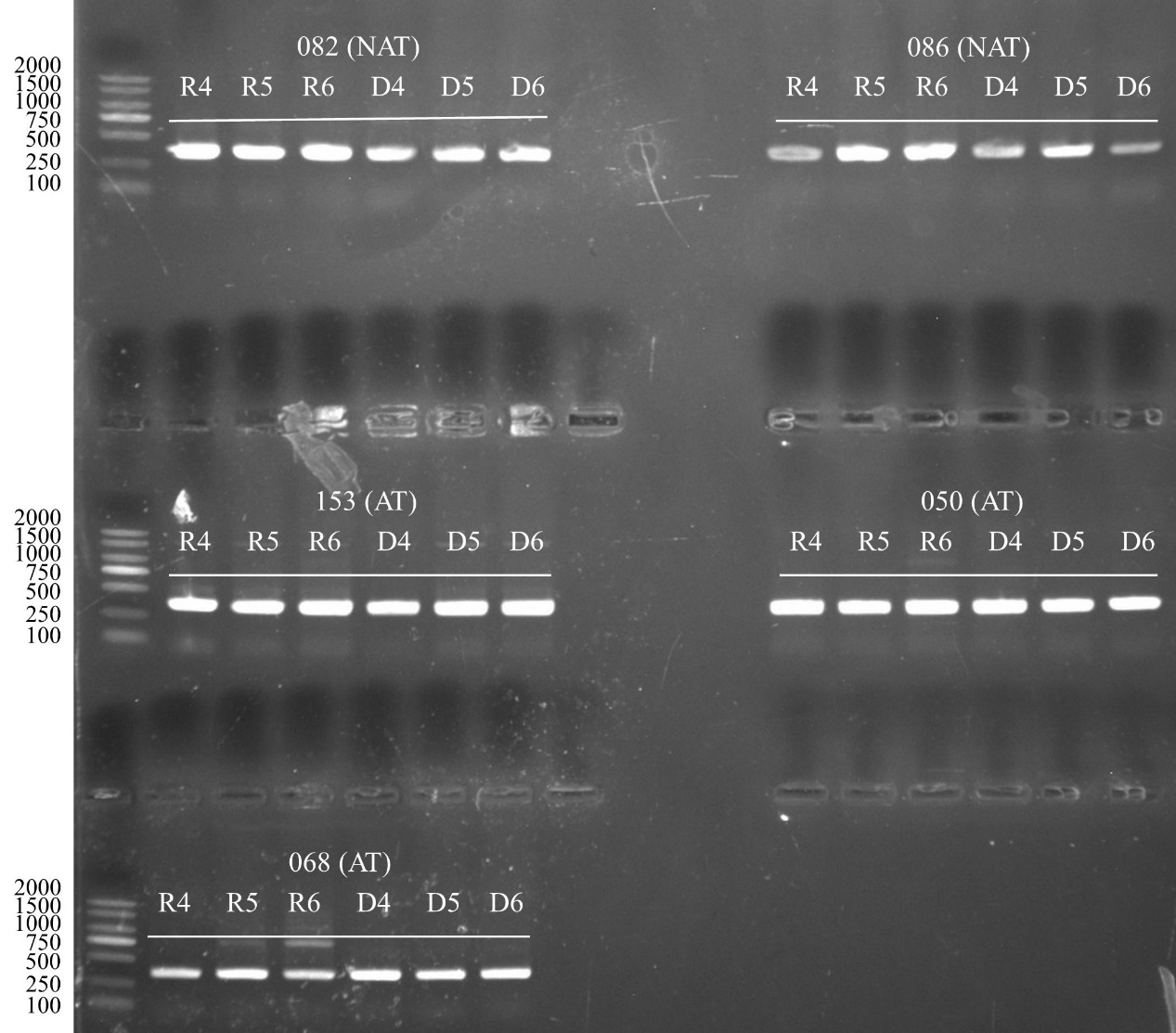
*

Figure S7-13. Expression analysis of *TCP4.*

*
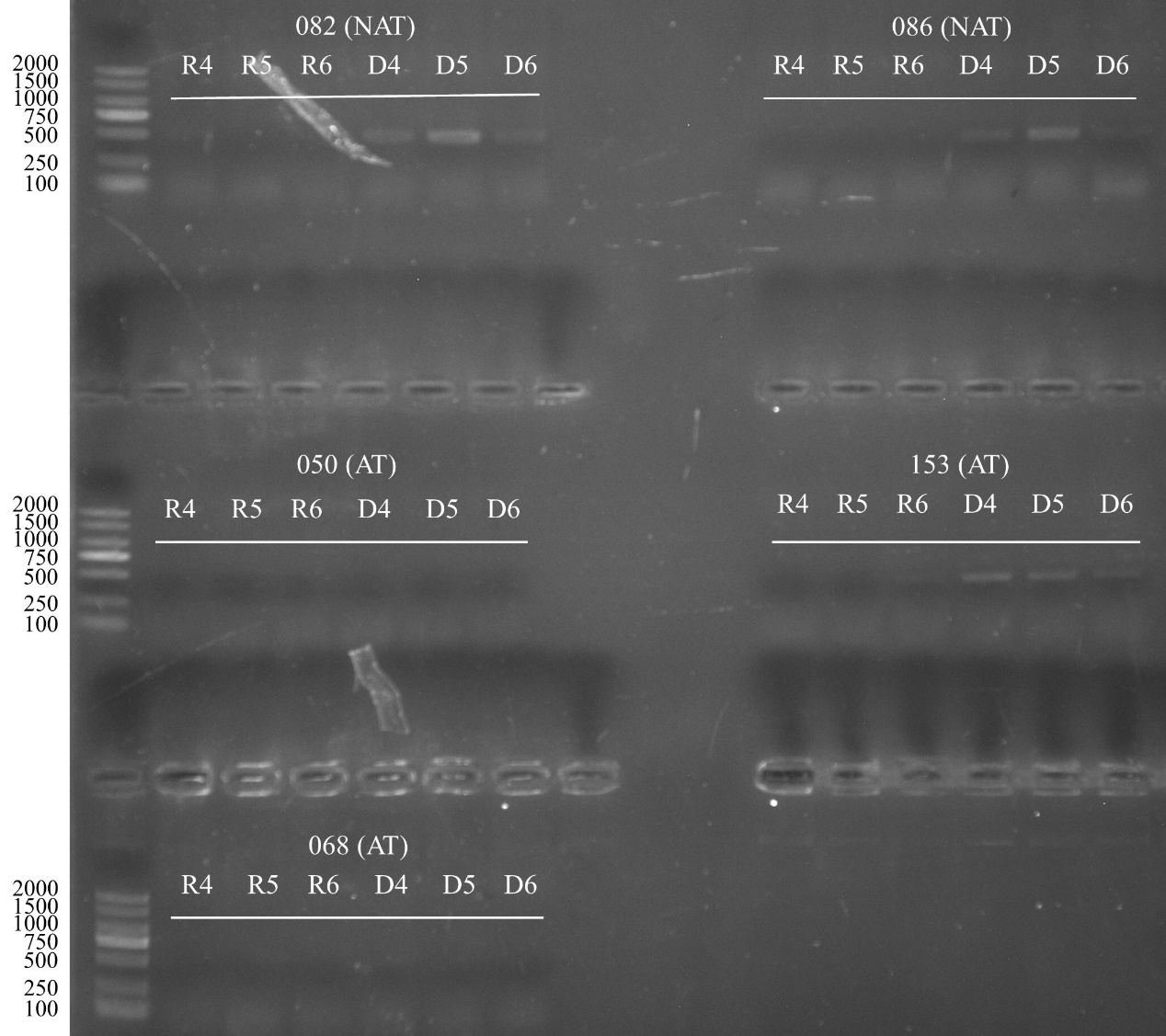
*

Figure S7-14. Expression analysis of *WRKY4.*

*
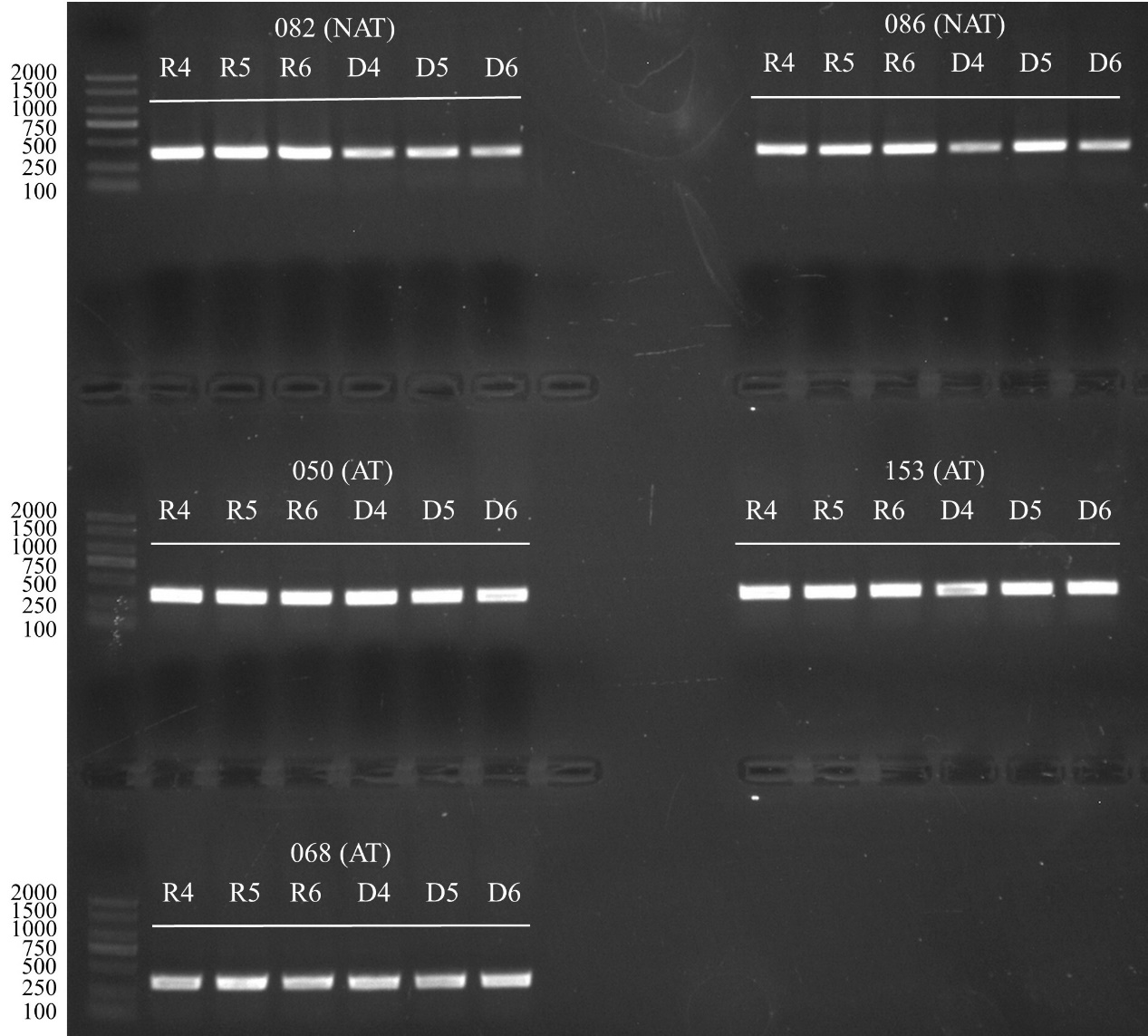
*

Figure S7-15. Expression analysis of *ARF8.*

*
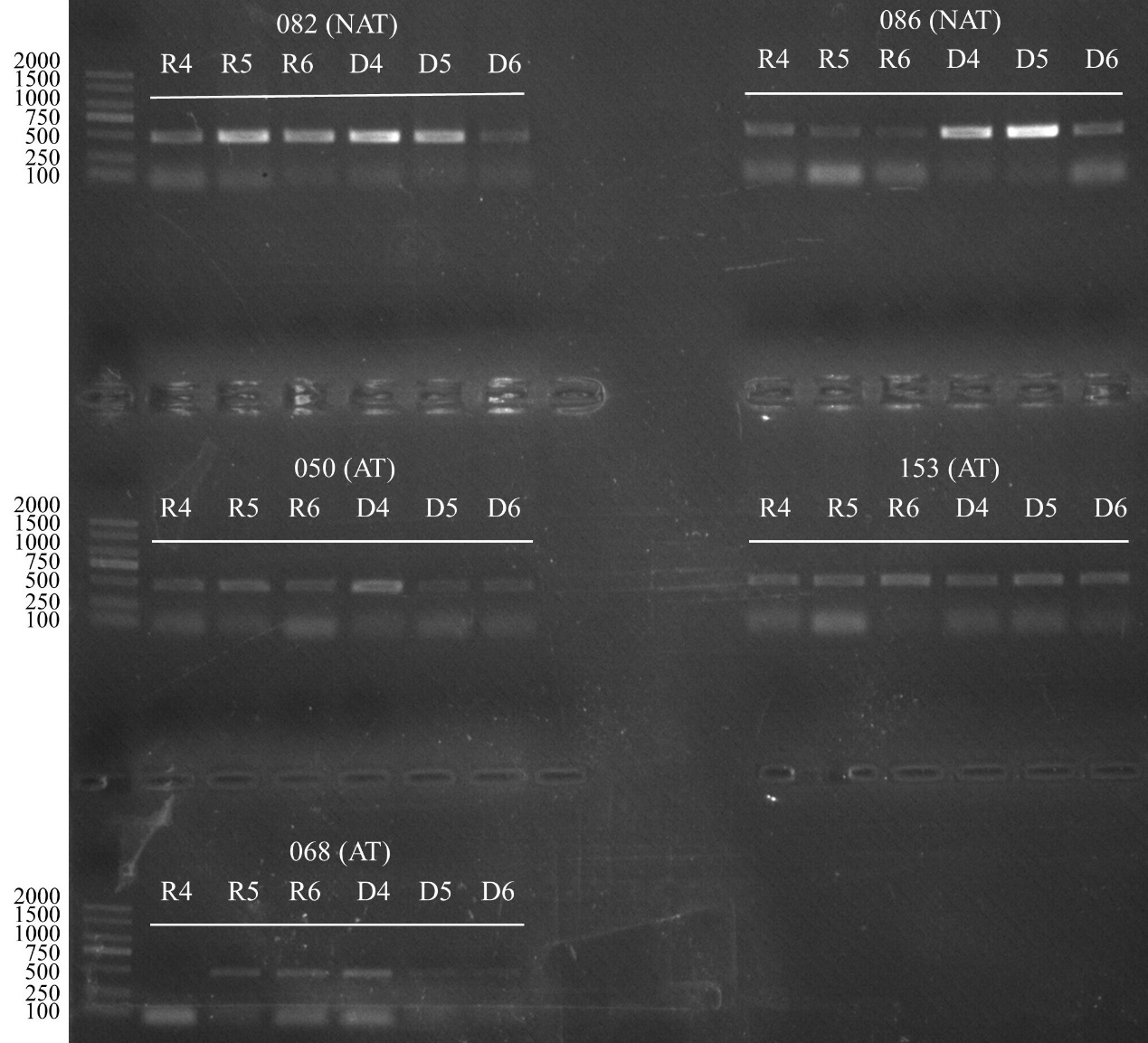
*

Figure S7-16. Expression analysis of *ARF19.*

*
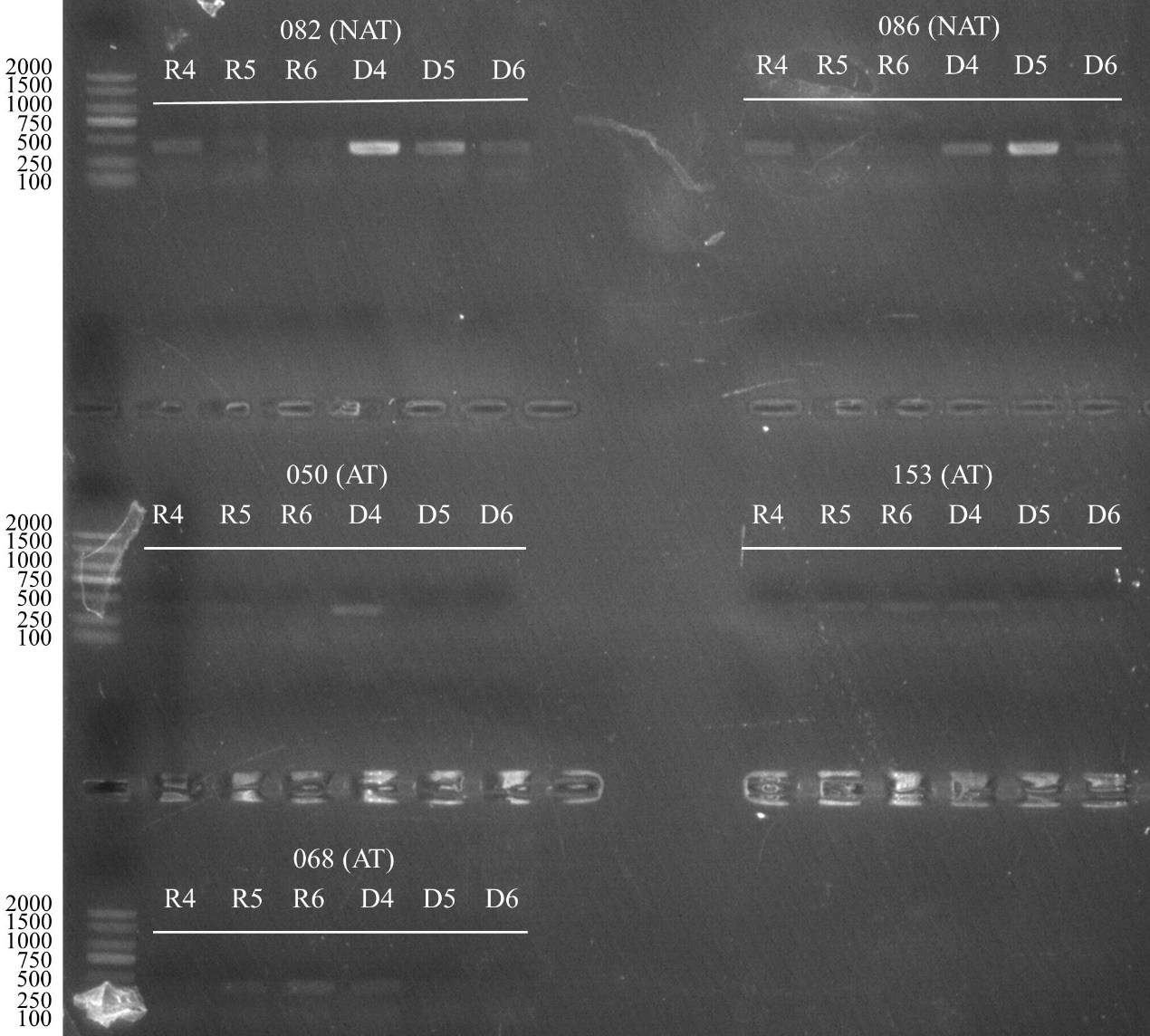
*

Figure S7-17. Expression analysis of *ARF16.*

*
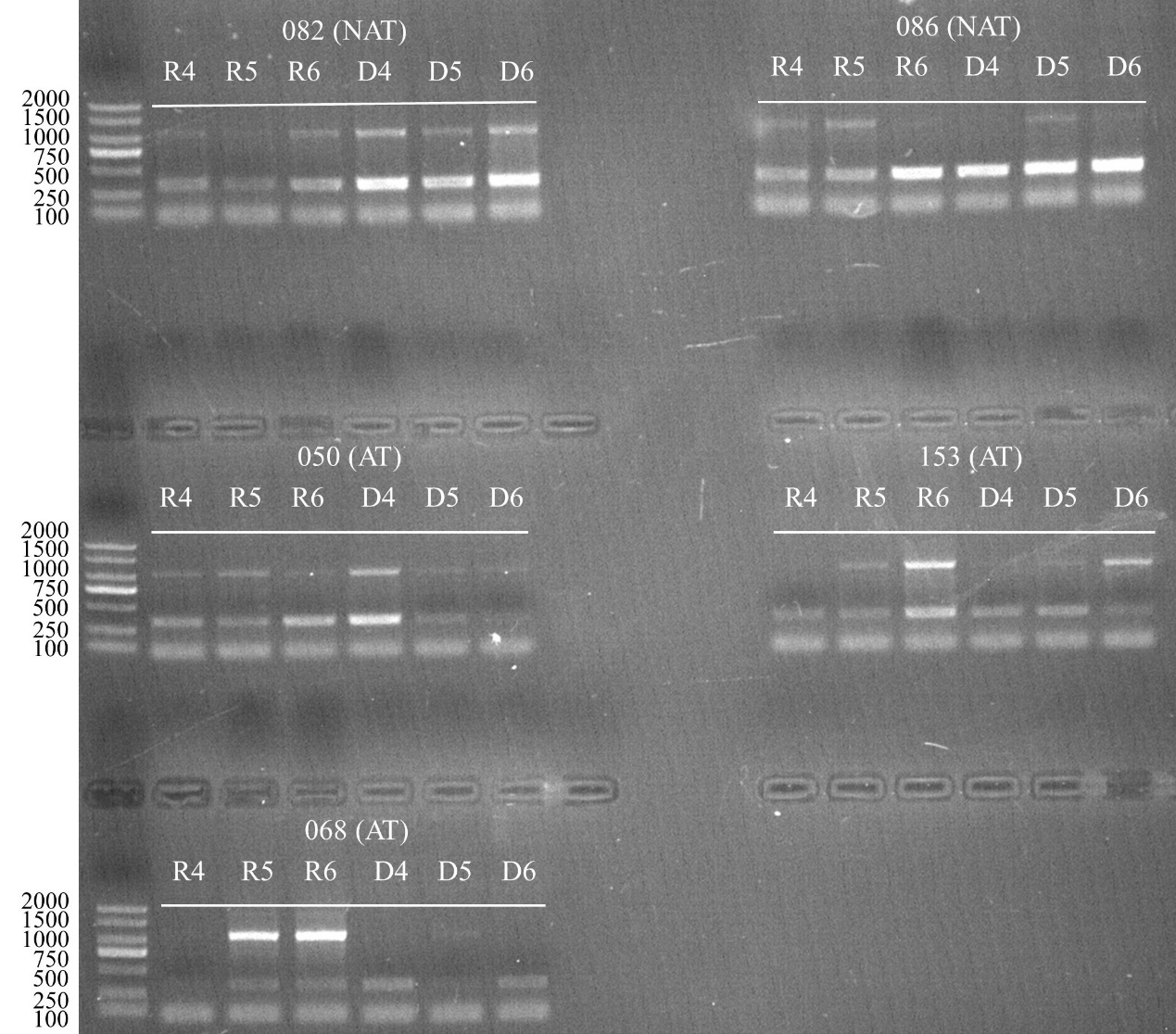
*

Figure S7-18. Expression analysis of *ERF010.*

*
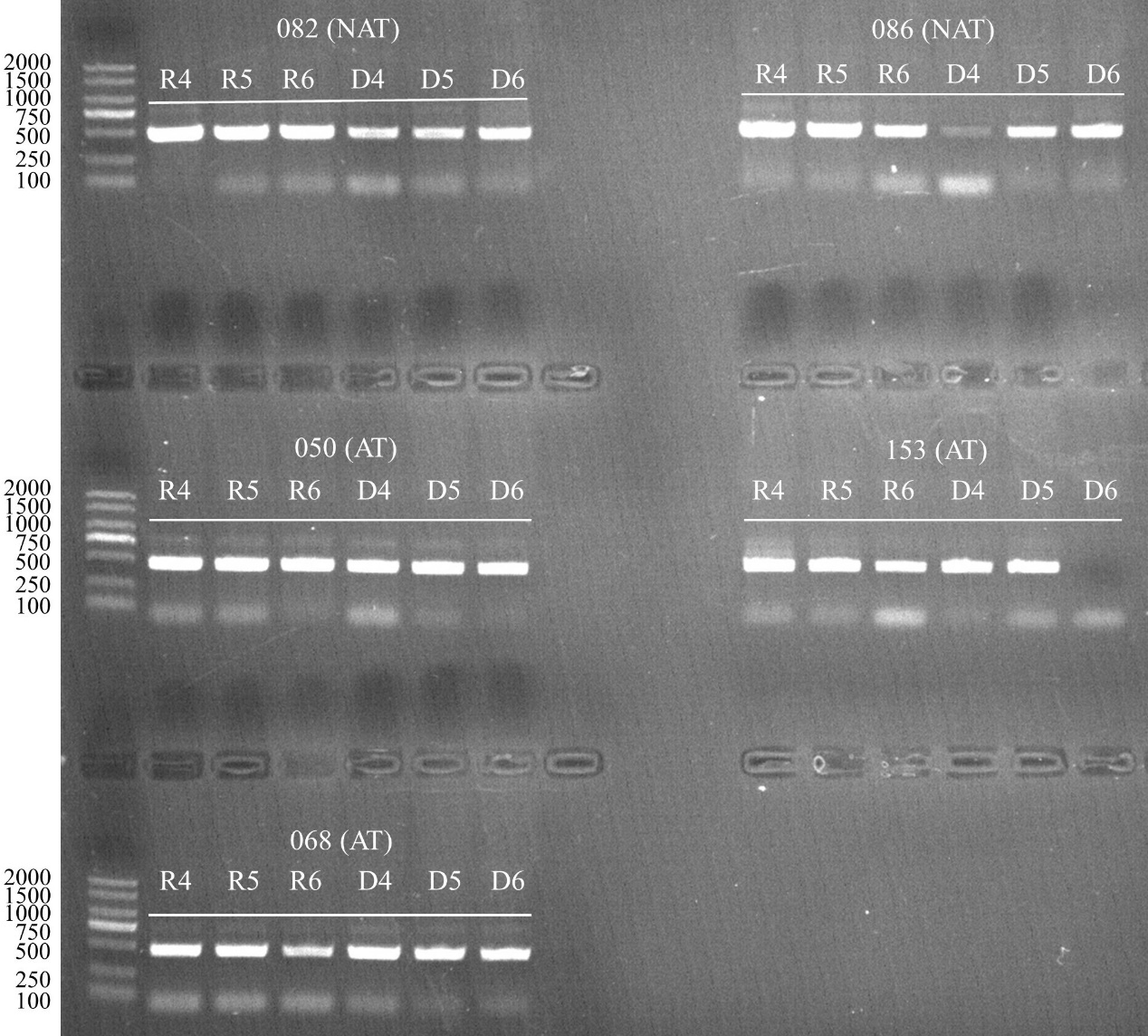
*

Figure S7-19. Expression analysis of *ERF.*

*
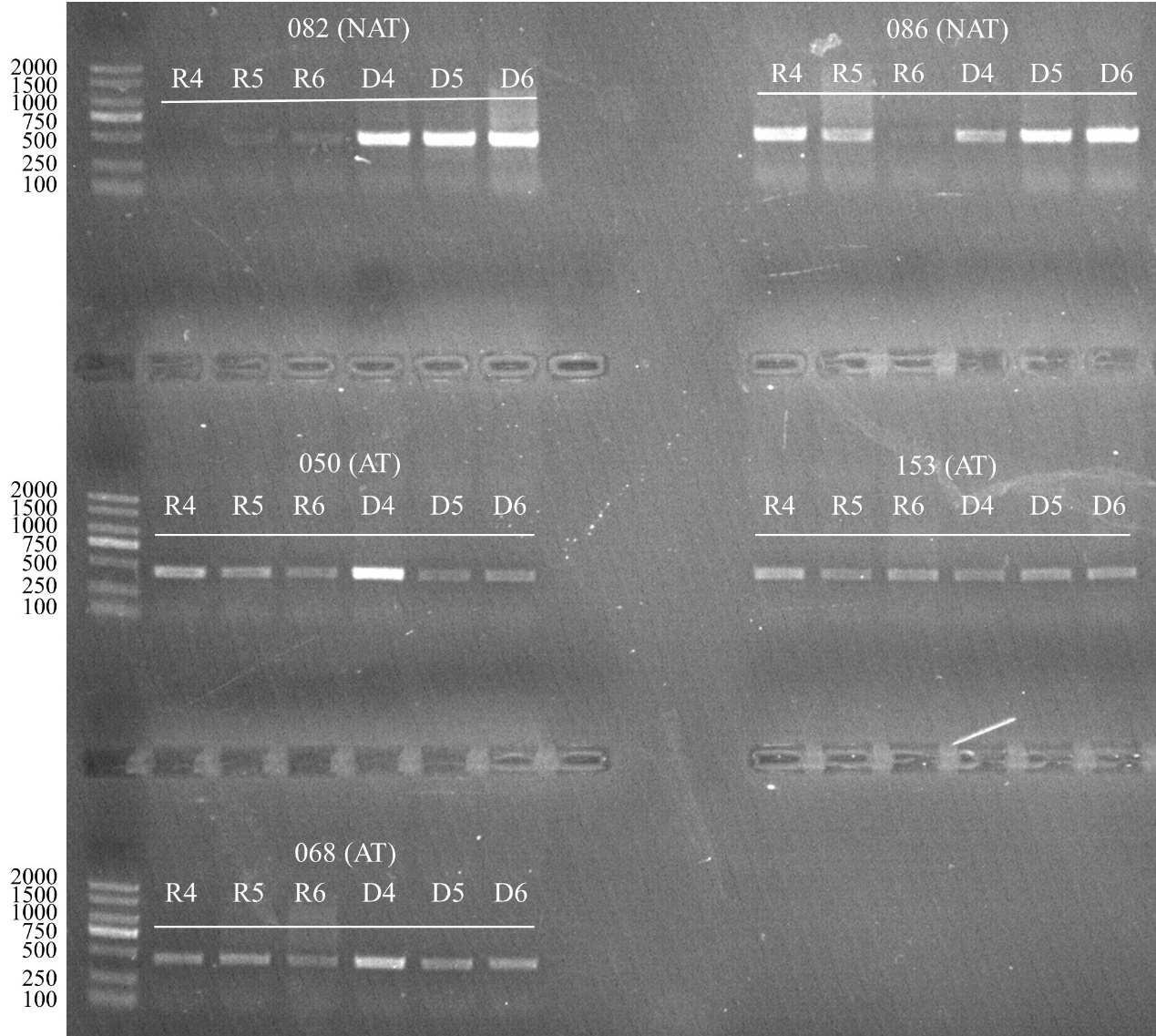
*

Figure S7-20. Expression analysis of *ERFPT16.*

*
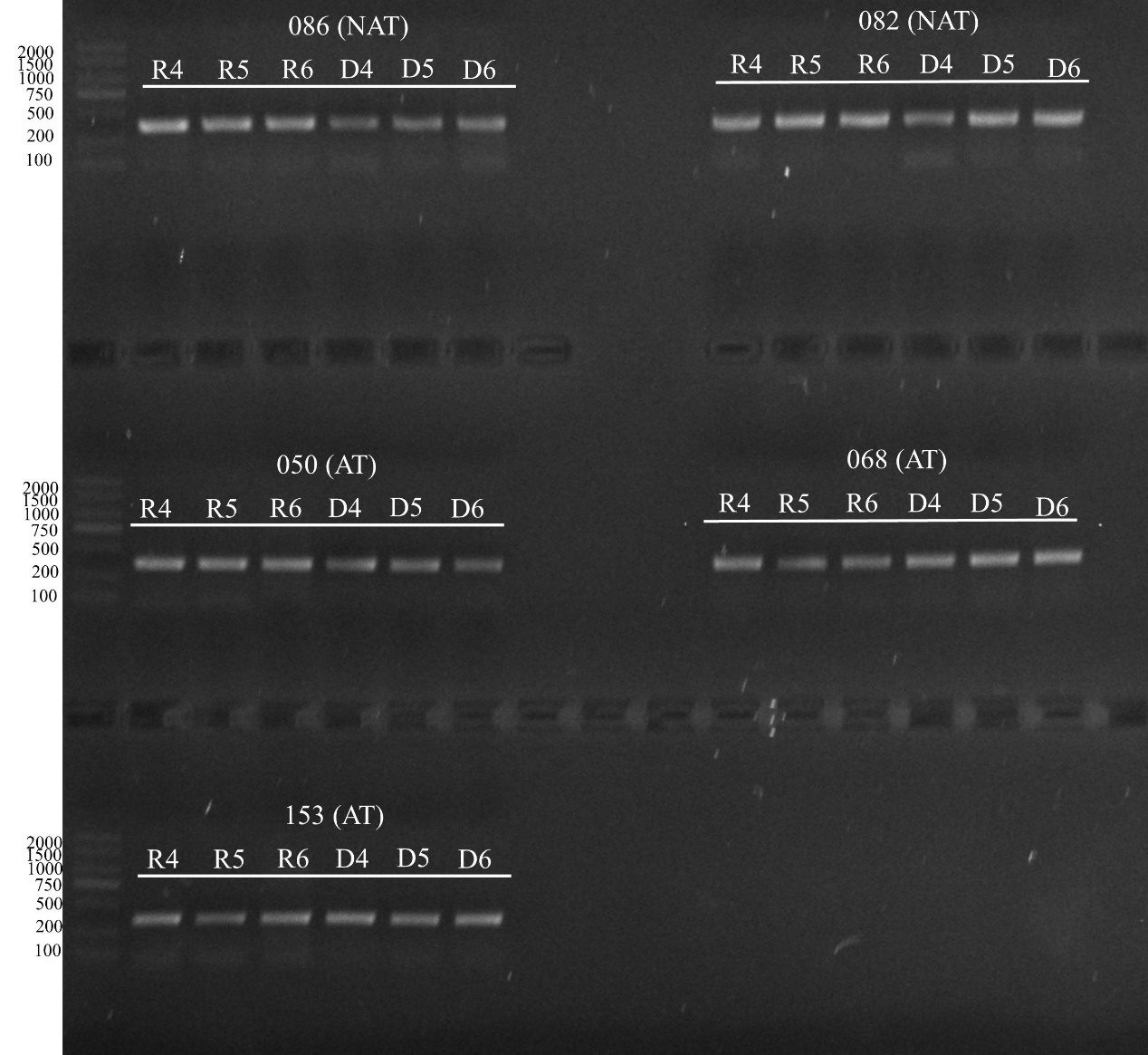
*

Figure S7-21. Expression analysis of *BALDIBIS.*

*
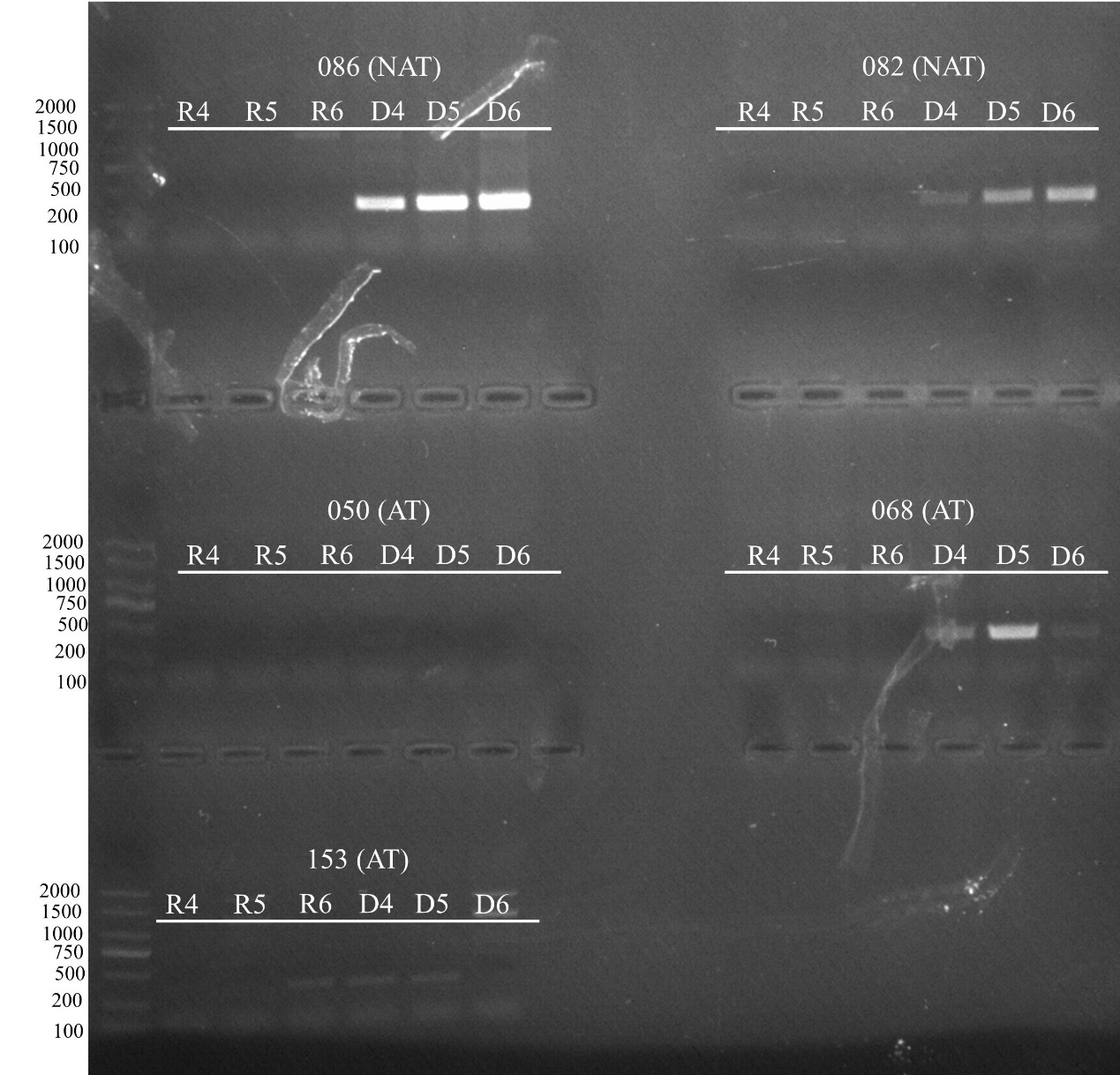
*

Figure S7-22. Expression analysis of *Elongation.*

*
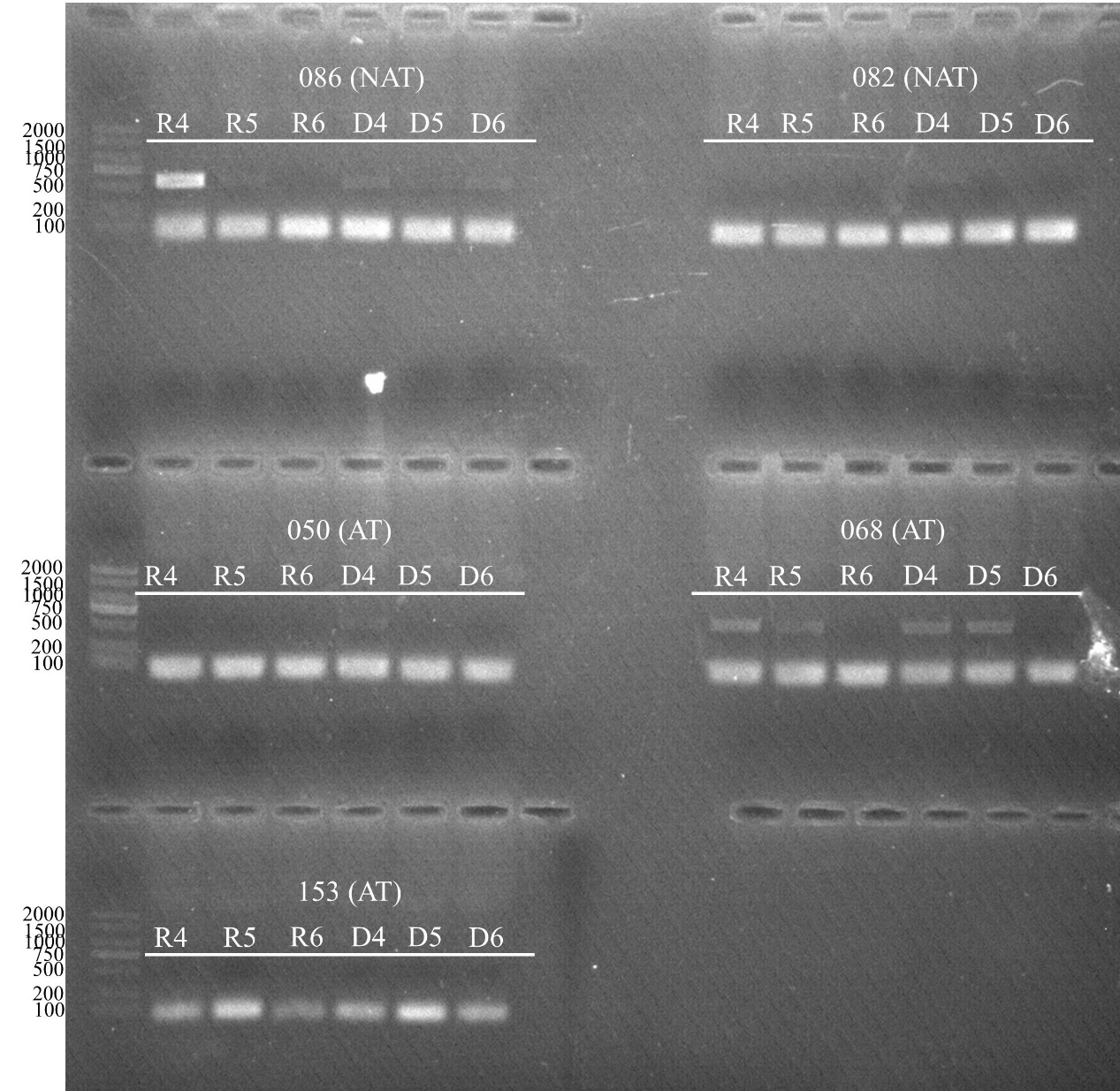
*

Figure S7-23. Expression analysis of *LHY.*

*
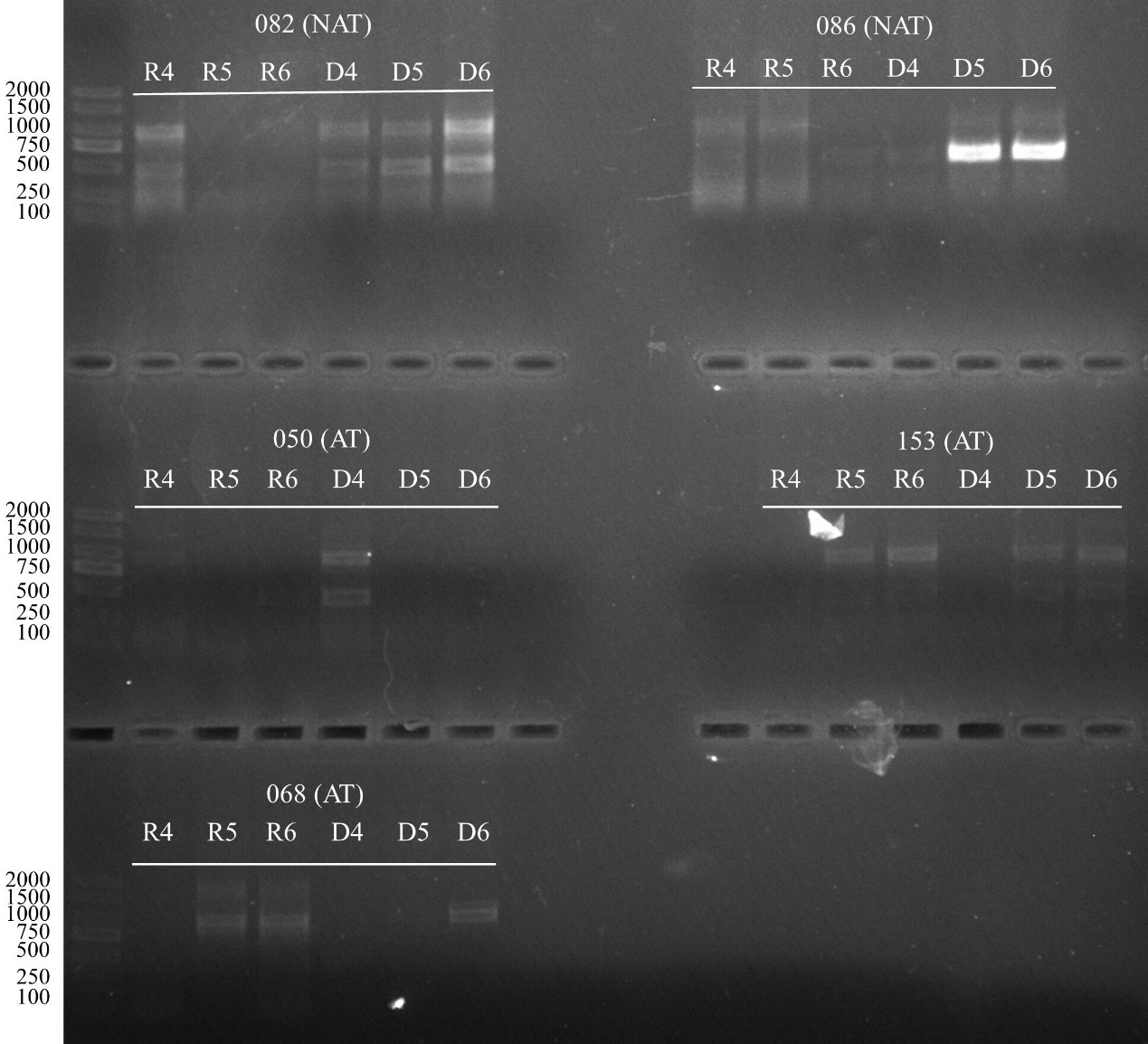
*

Figure S7-24. Expression analysis of *ERF110.*

*
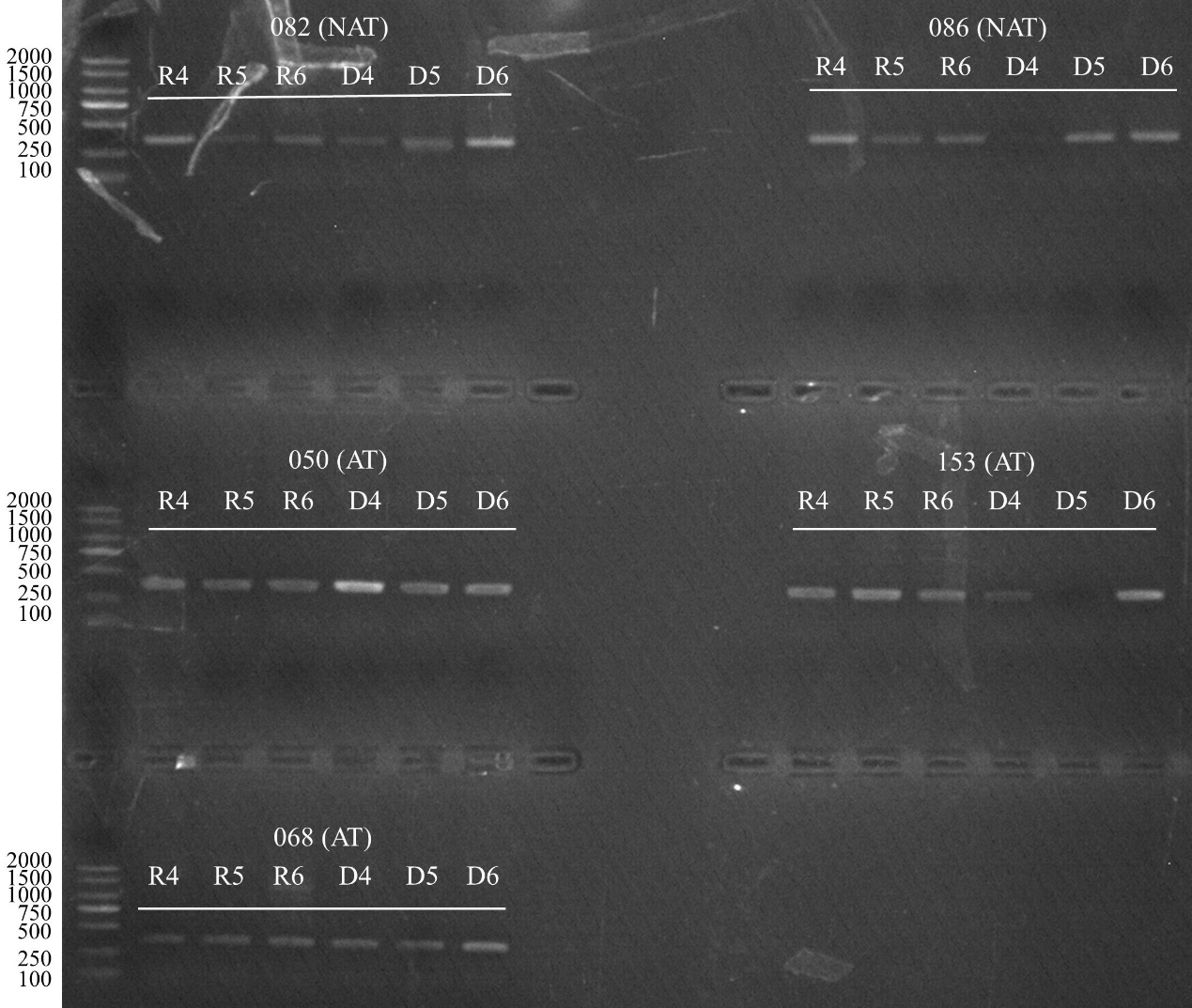
*

Figure S7-25. Expression analysis of *MYB8.*

*
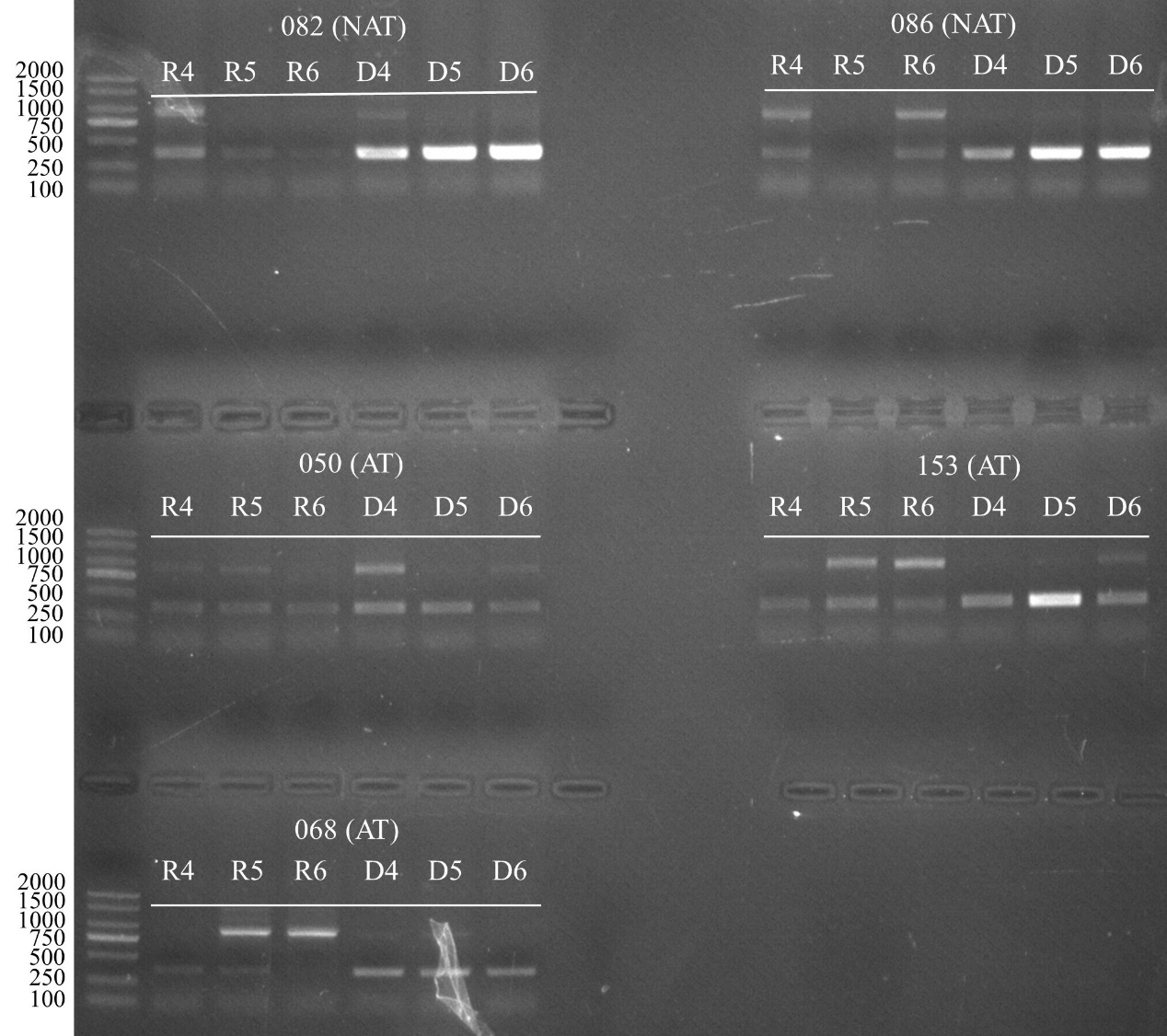
*

Figure S7-26. Expression analysis of *MYB52.*

*
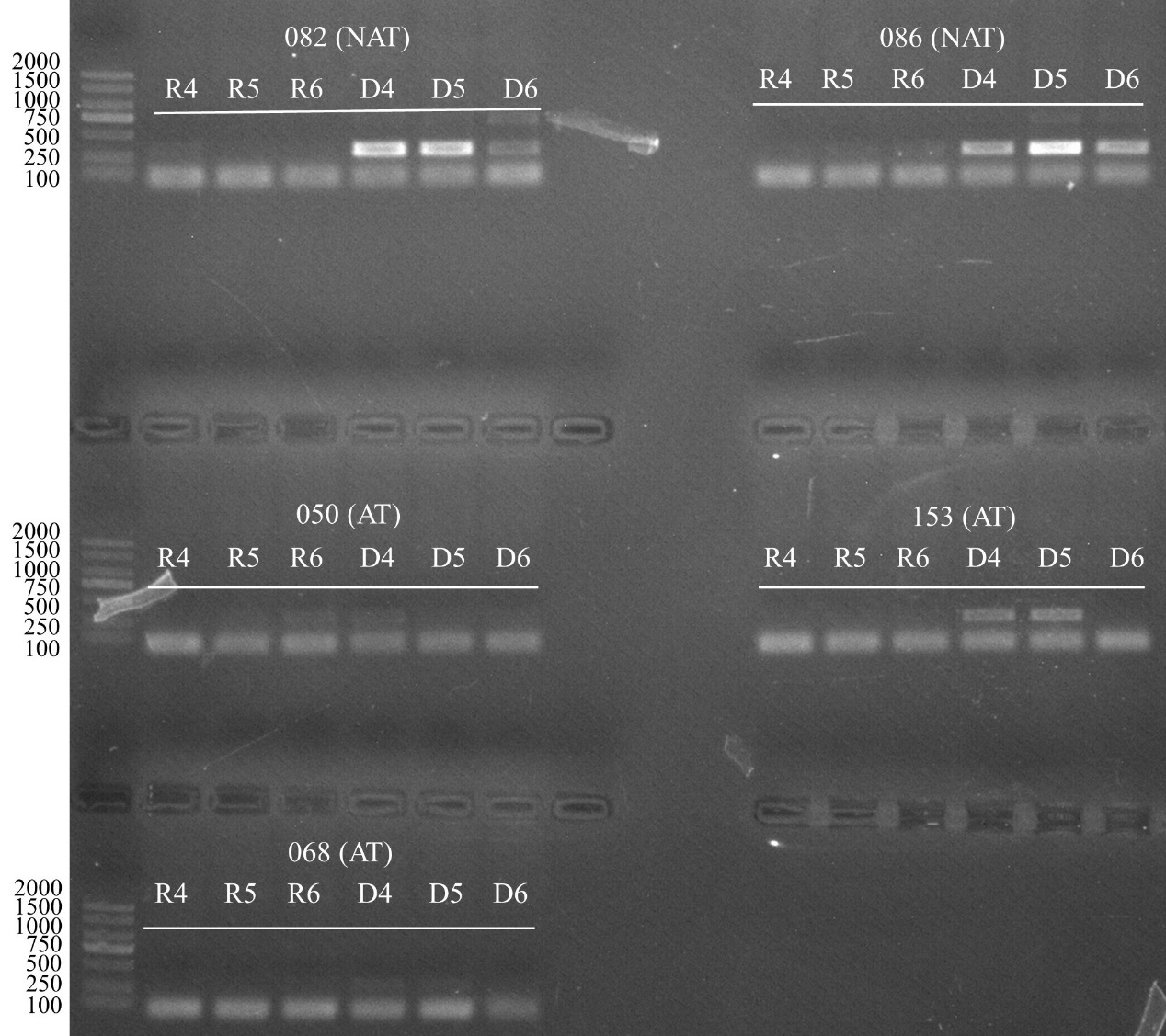
*

Figure S7-27. Expression analysis of *WUS.*

*
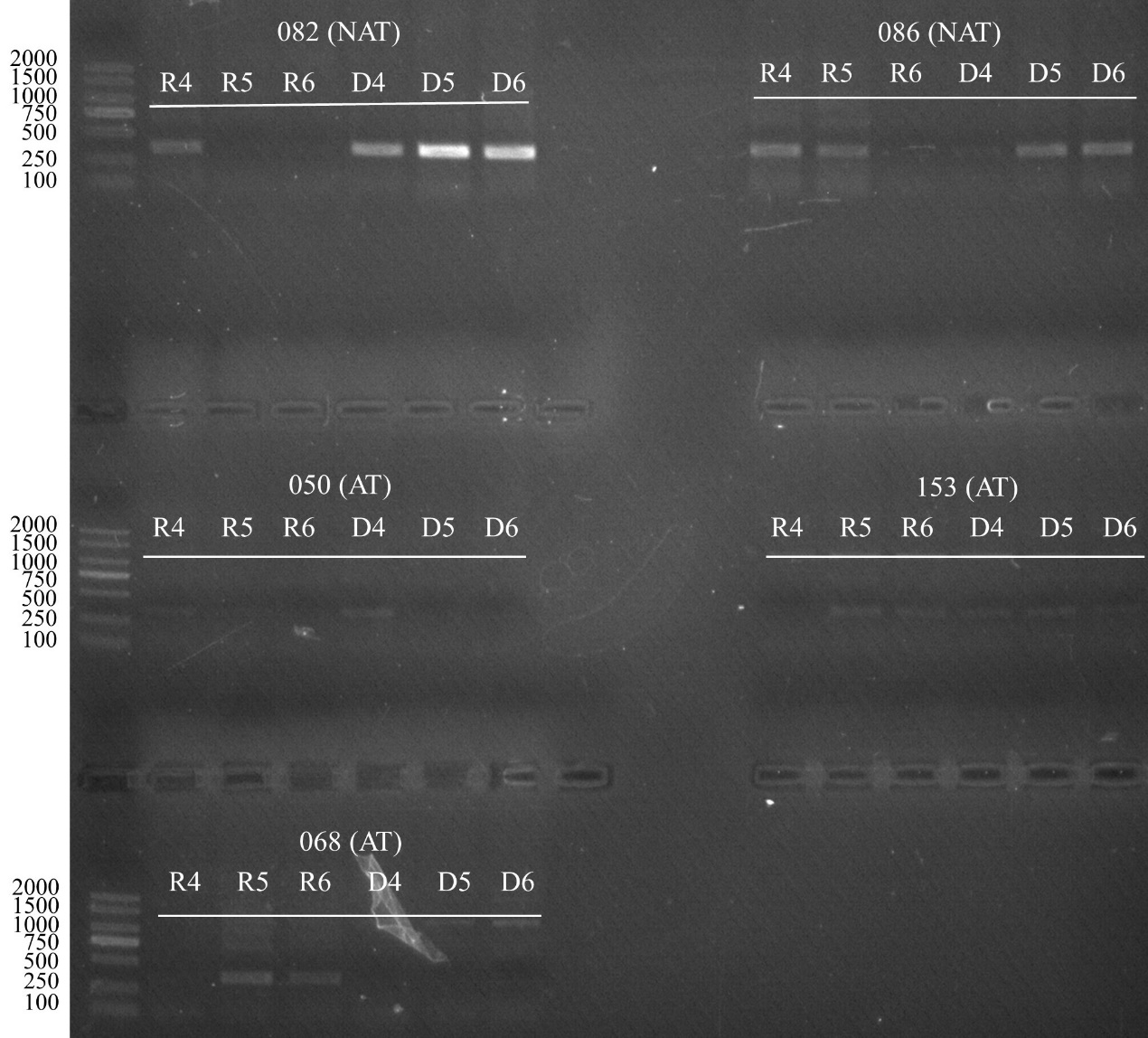
*

Figure S7-28. Expression analysis of *bHLH52.*

*
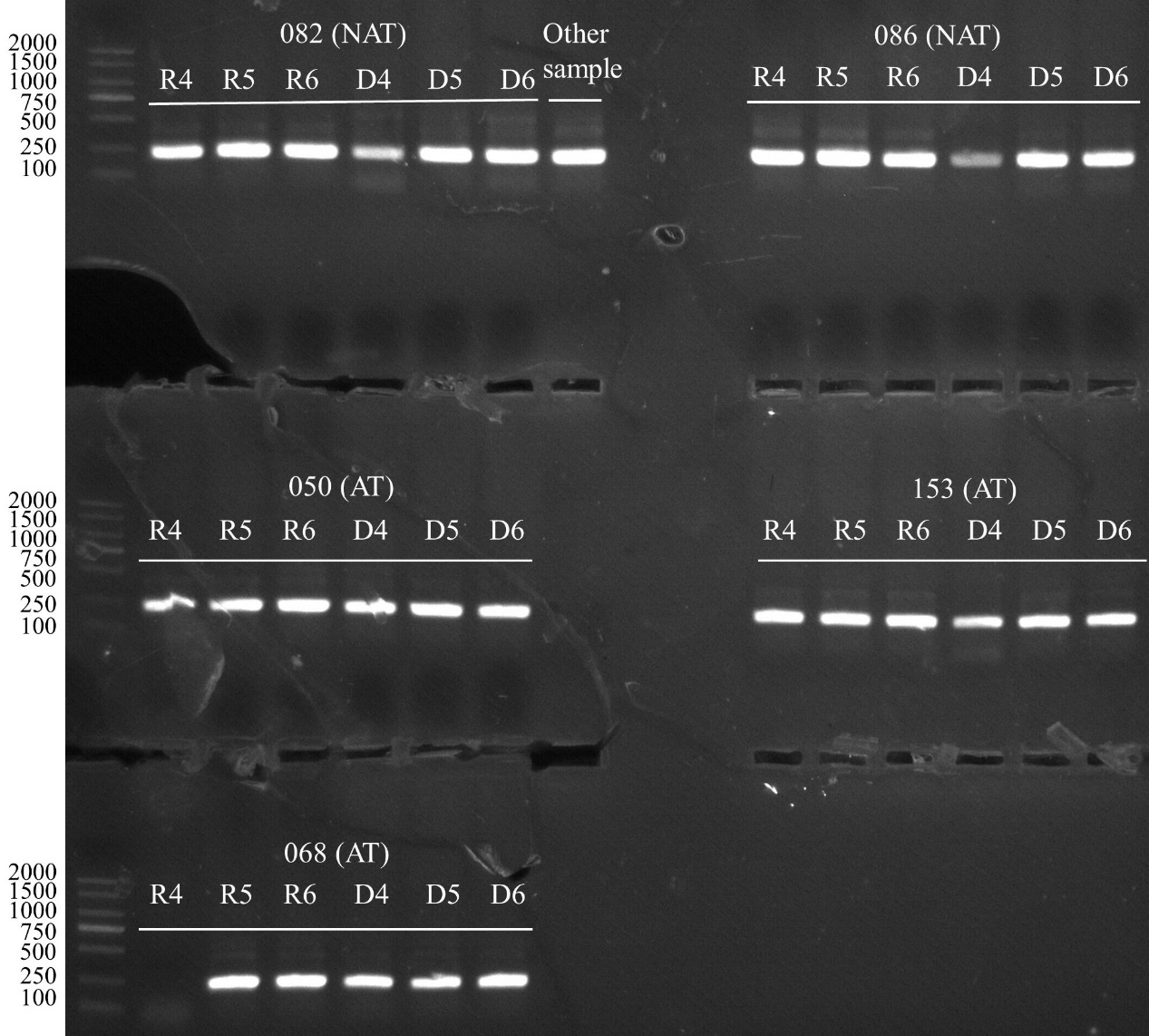
*

Figure S7-29. Expression analysis of *PRE5.*

*
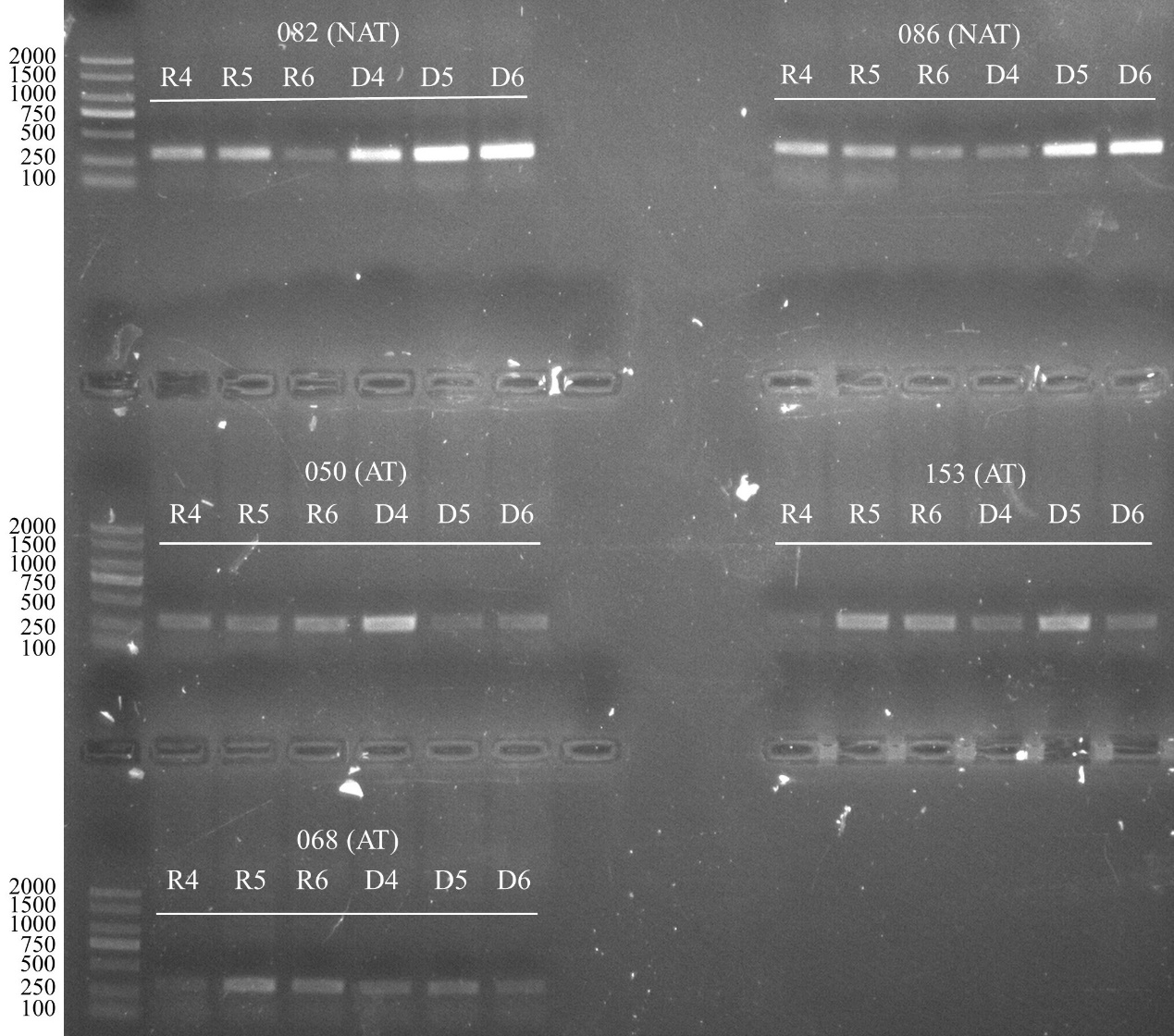
*

Figure S7-30. Expression analysis of *WRKY.*

*
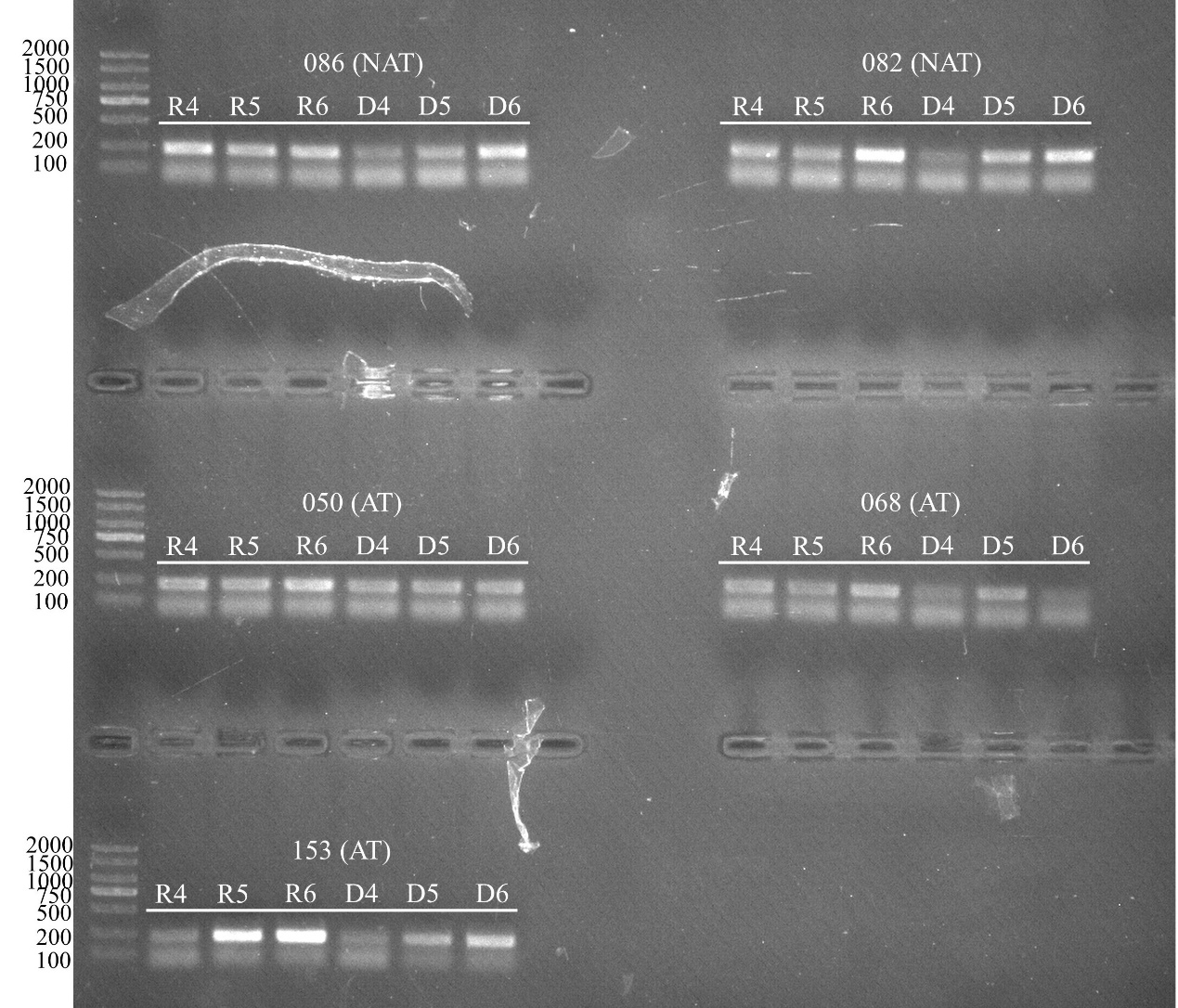
*

Figure S7-31. Expression analysis of *RADIALIS.*

*
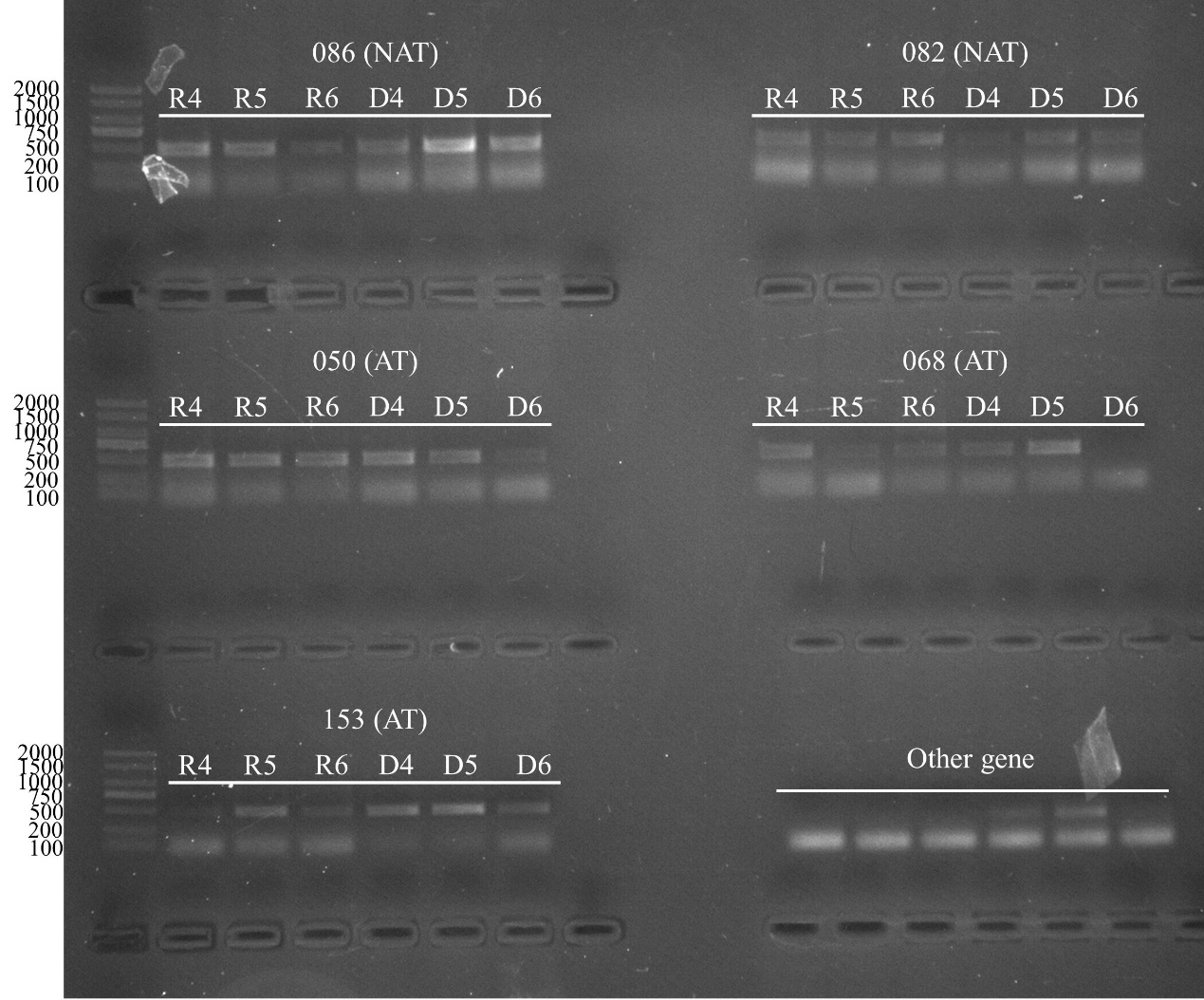
*

Figure S7-32. Expression analysis of *CCR4.*

*
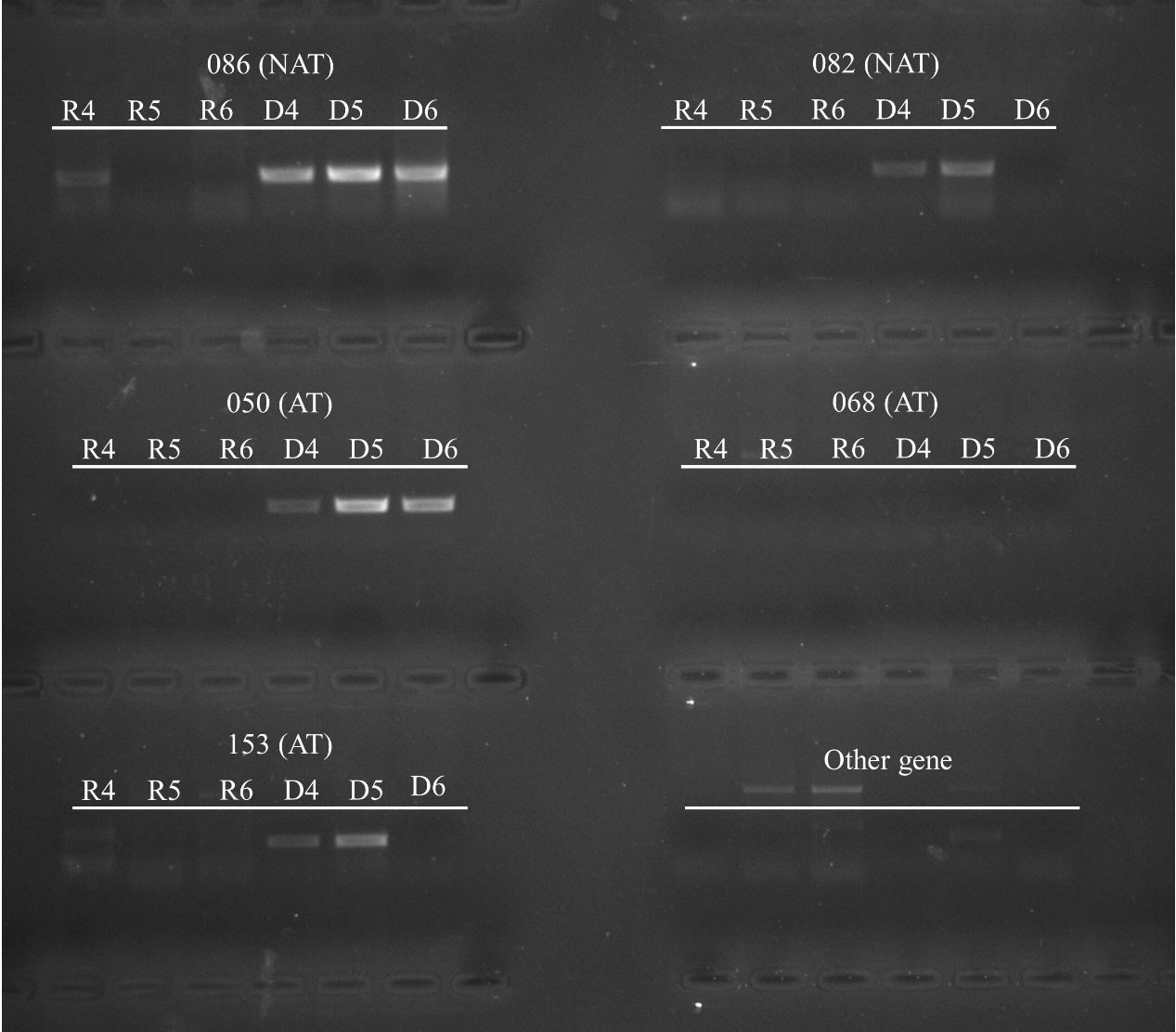
*

Figure S7-33. Expression analysis of *WRKY31.*

*
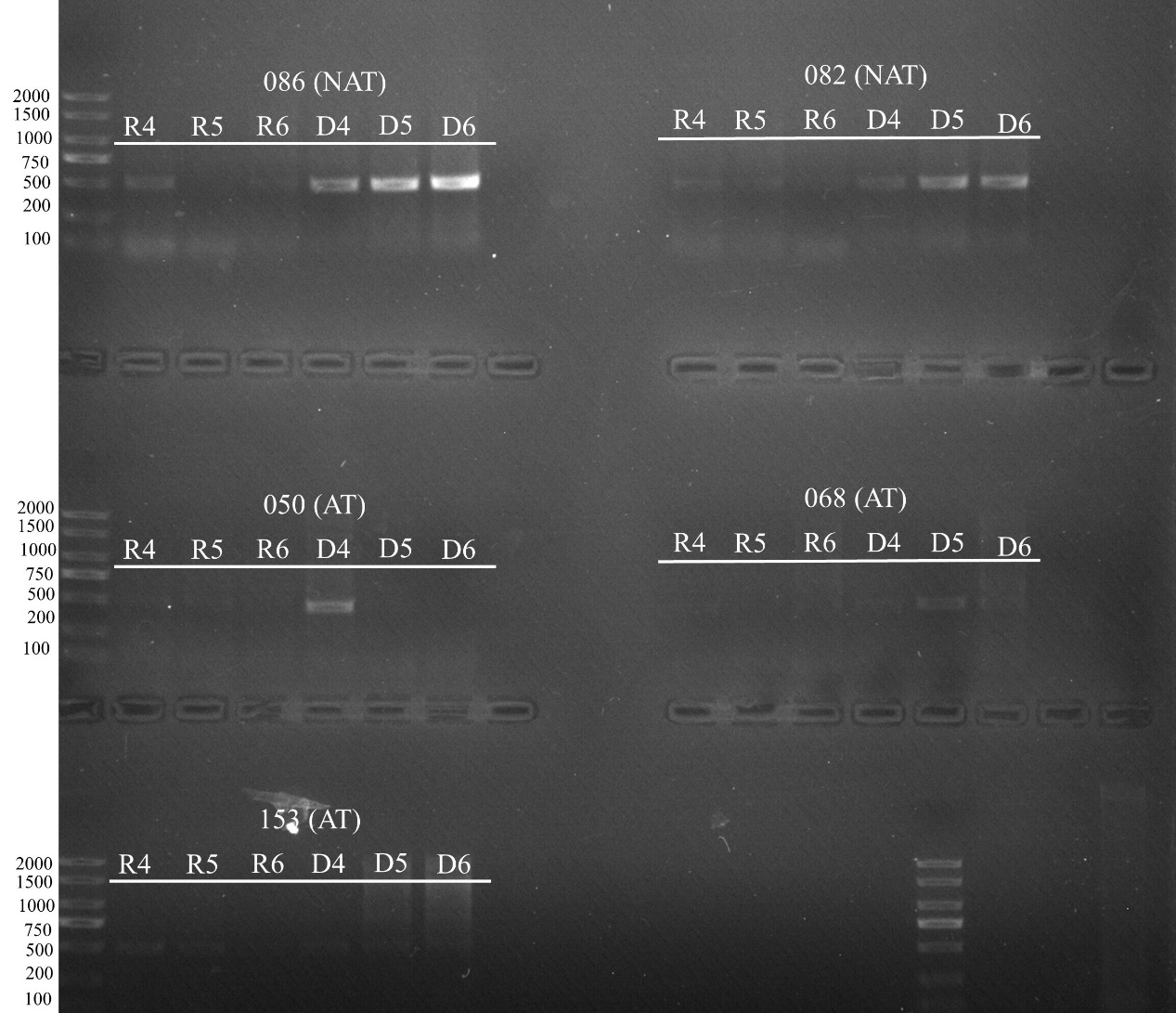
*

Figure S7-34. Expression analysis of *bHLH18.*

*
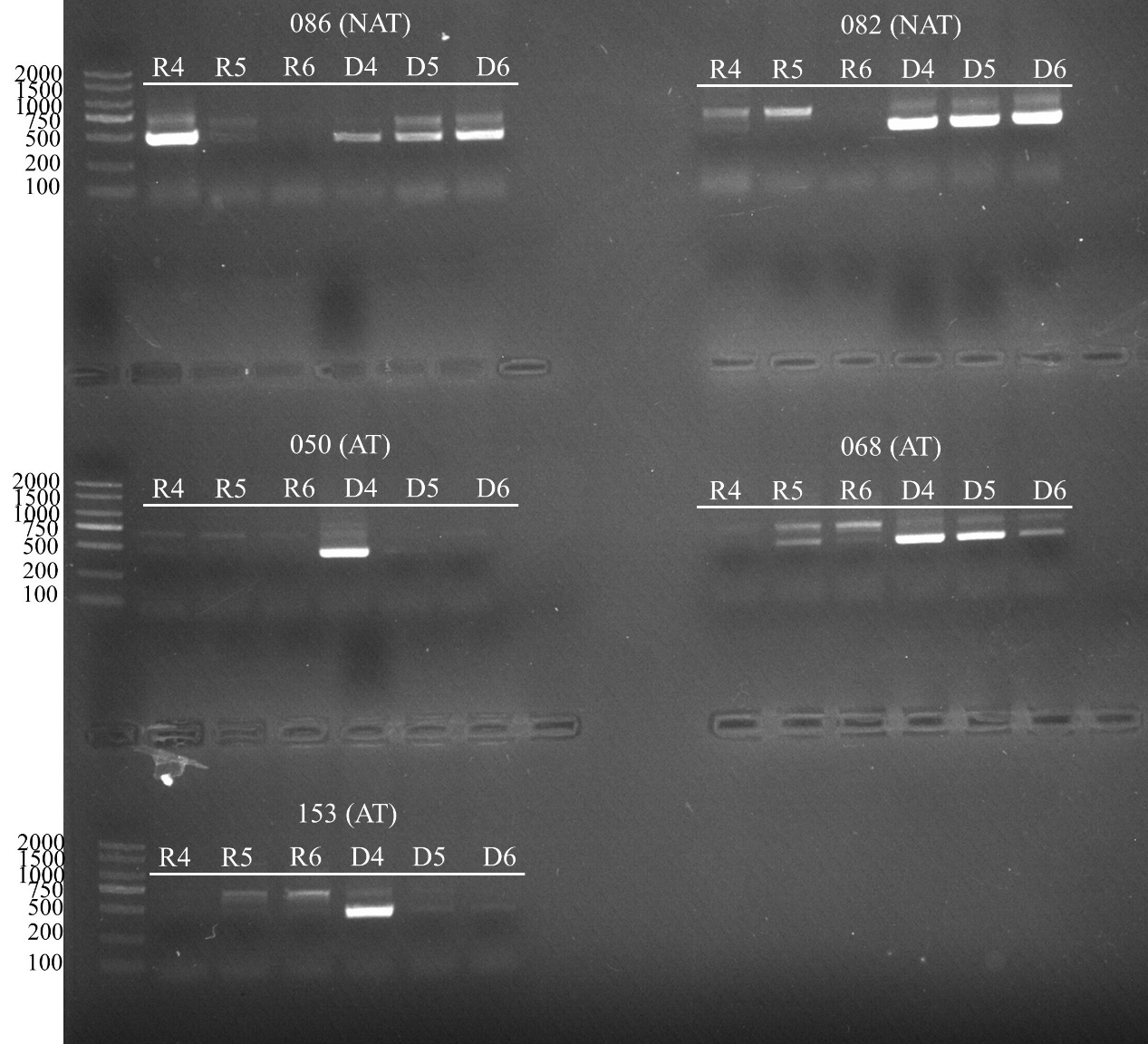
*

Figure S7-35. Expression analysis of *MYB97.*

*
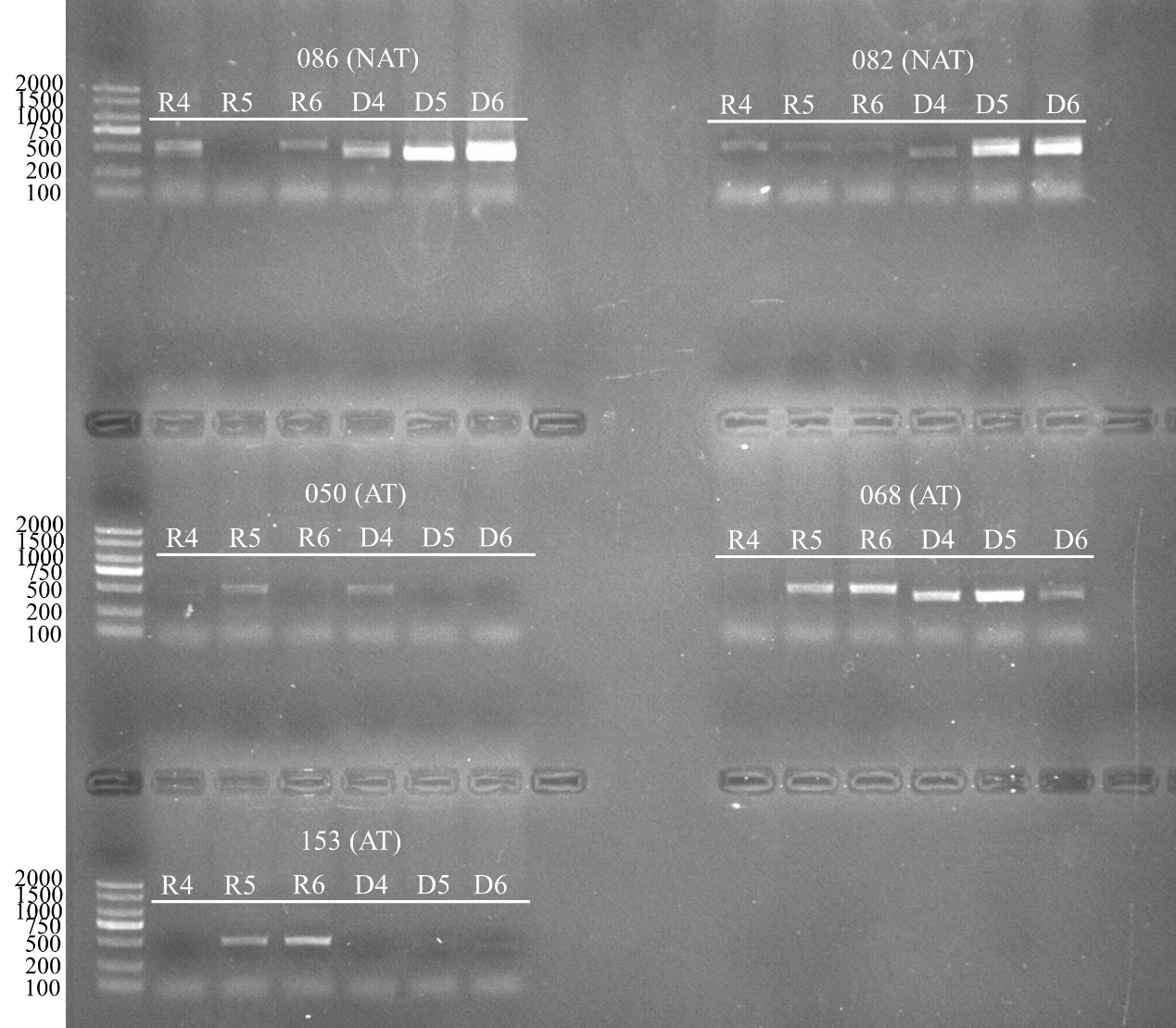
*

Figure S7-36. Expression analysis of *DUO.*

*
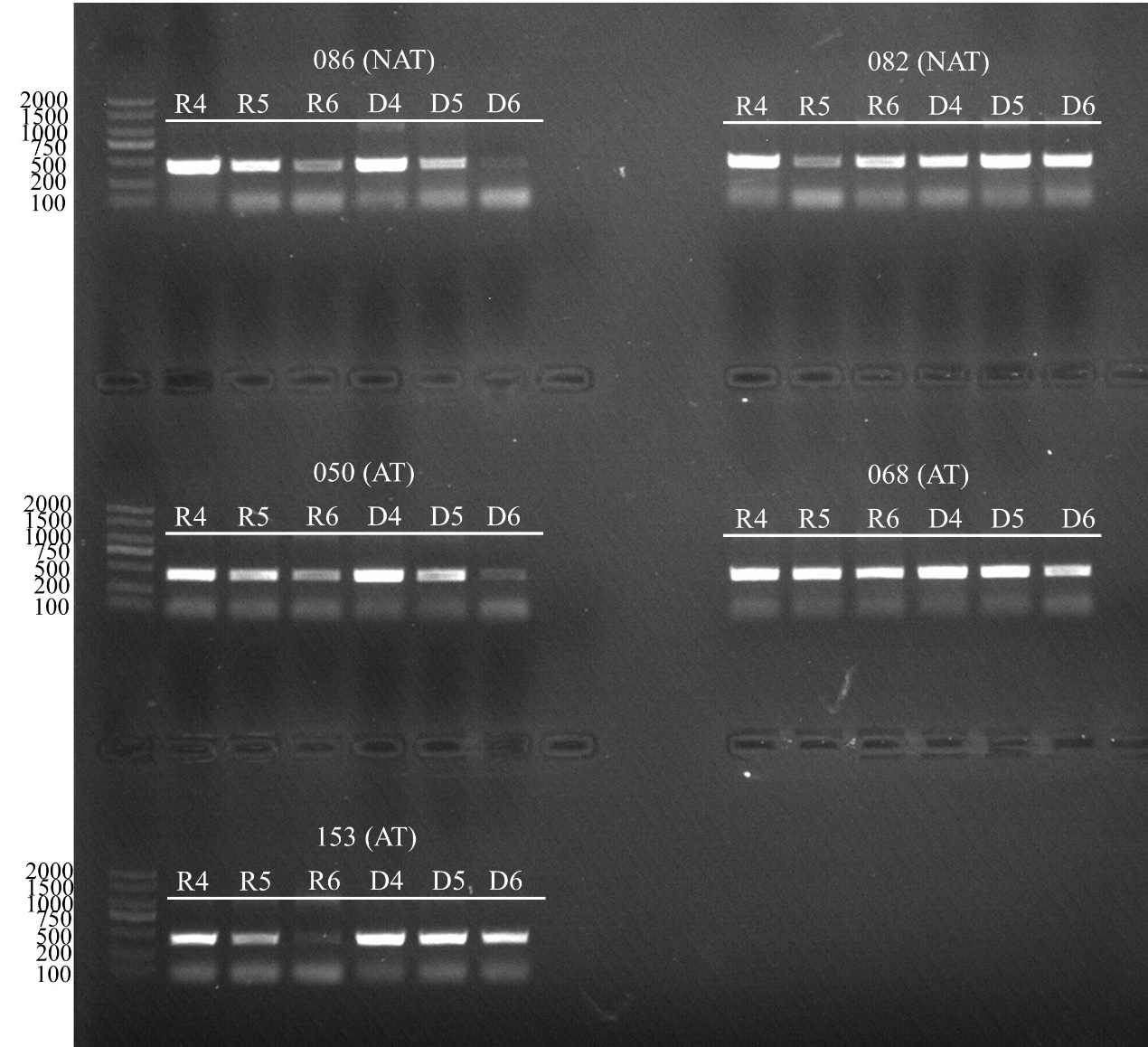
*

Figure S7-37. Expression analysis of *SQUA.*

*
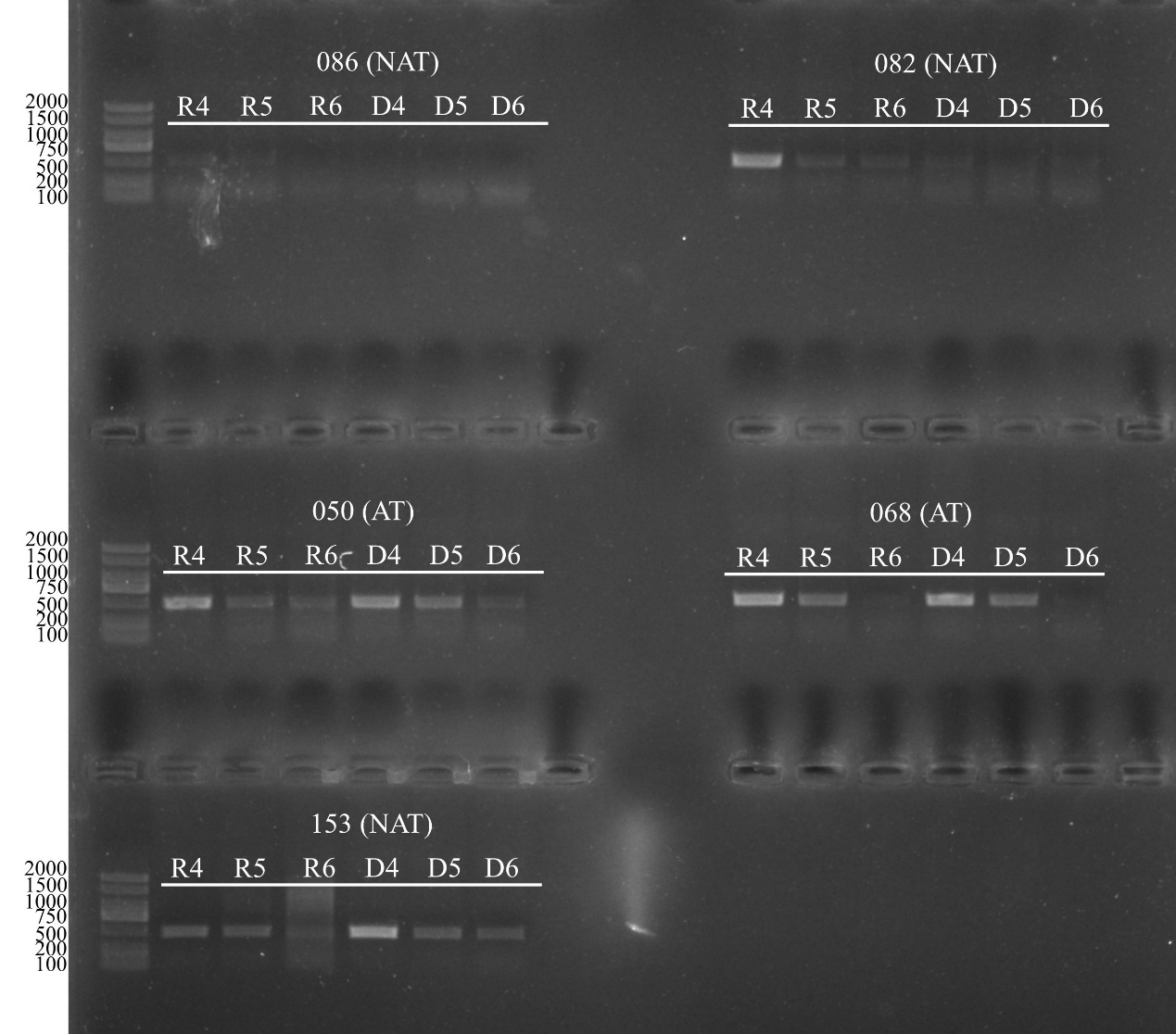
*

Figure S7-38. Expression analysis of *MYC2.*

*
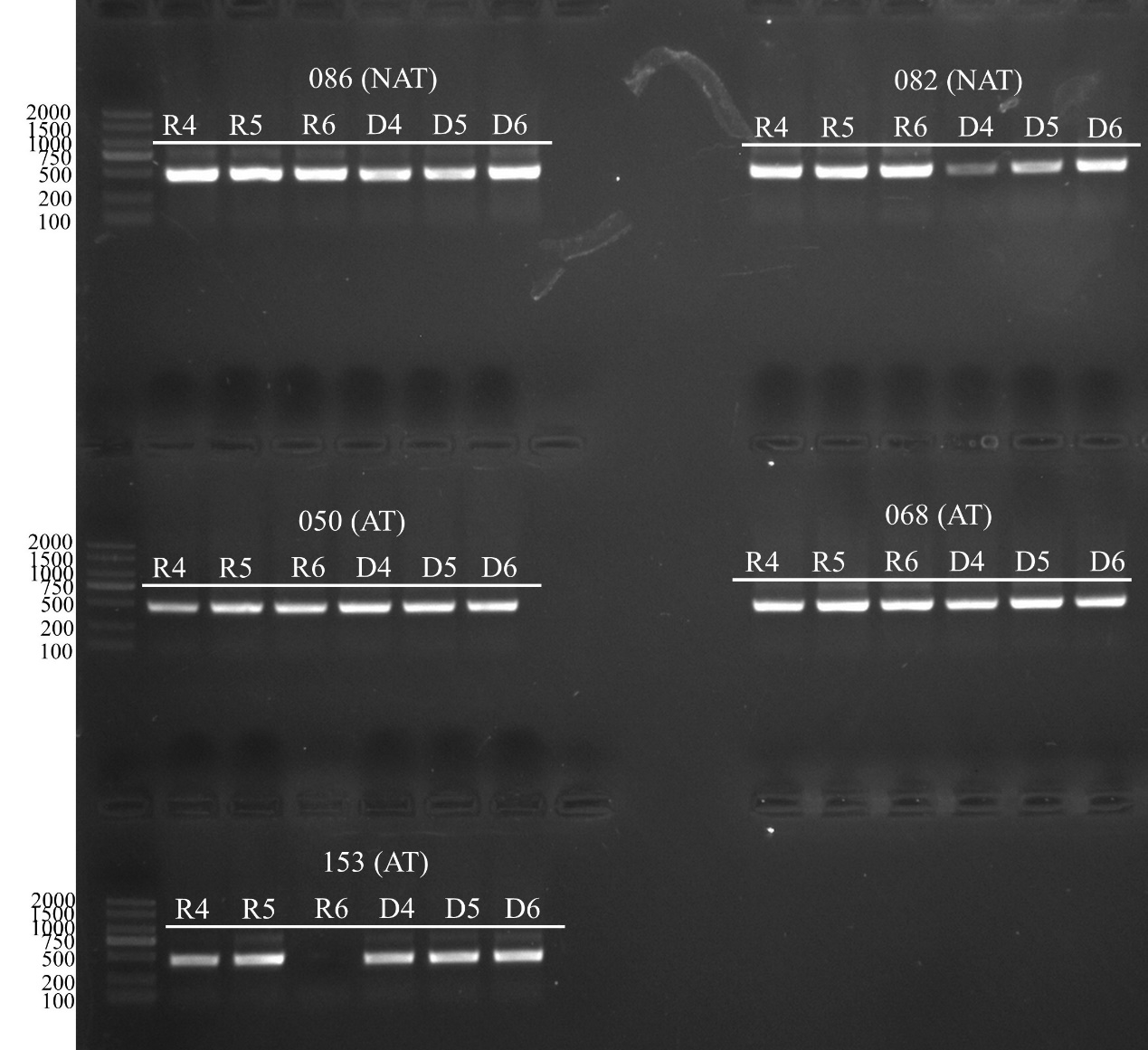
*

Figure S7-39. Expression analysis of *ERF.*

*
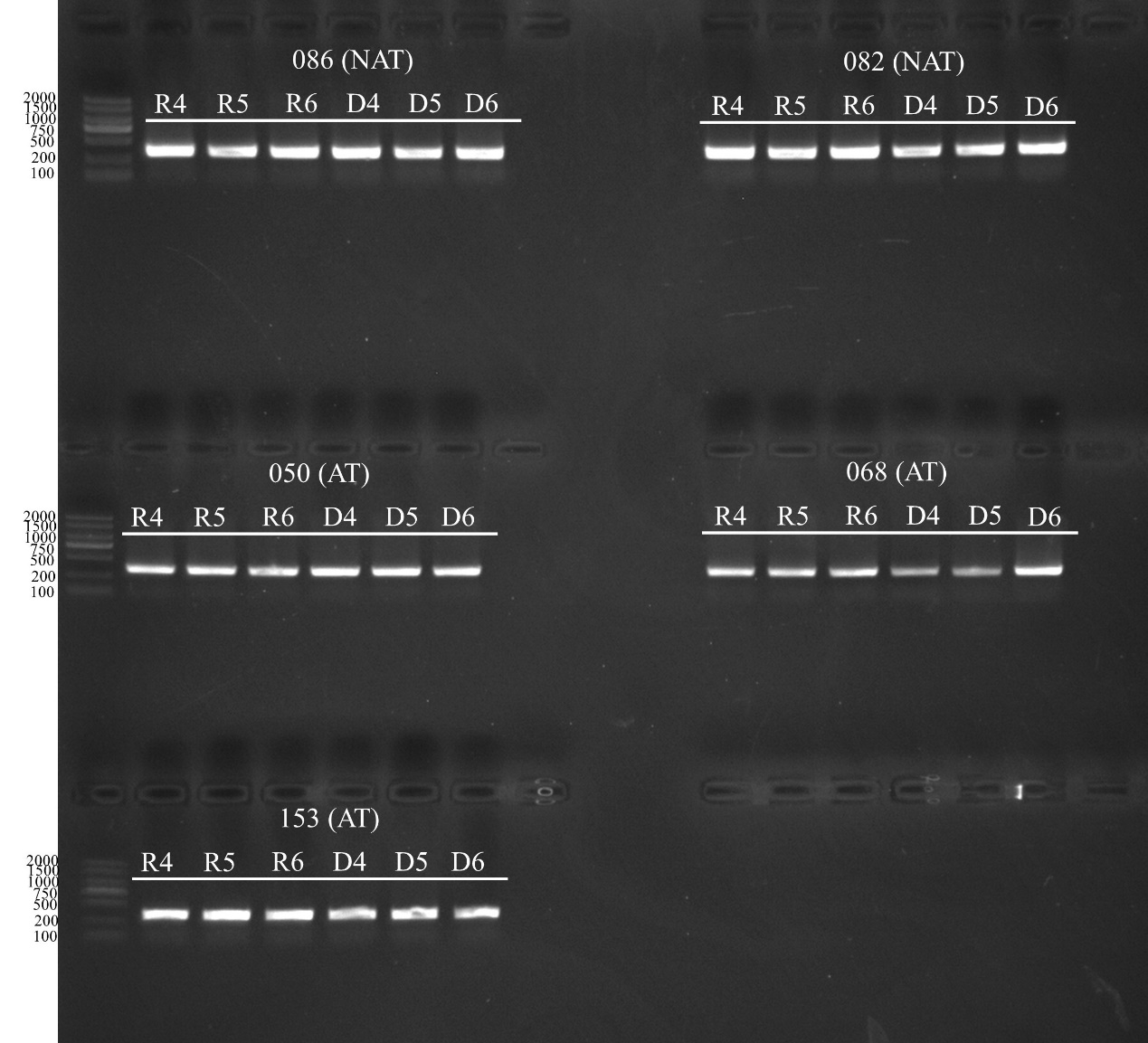
*

Figure S7-40. Expression analysis of *TALE.*

*
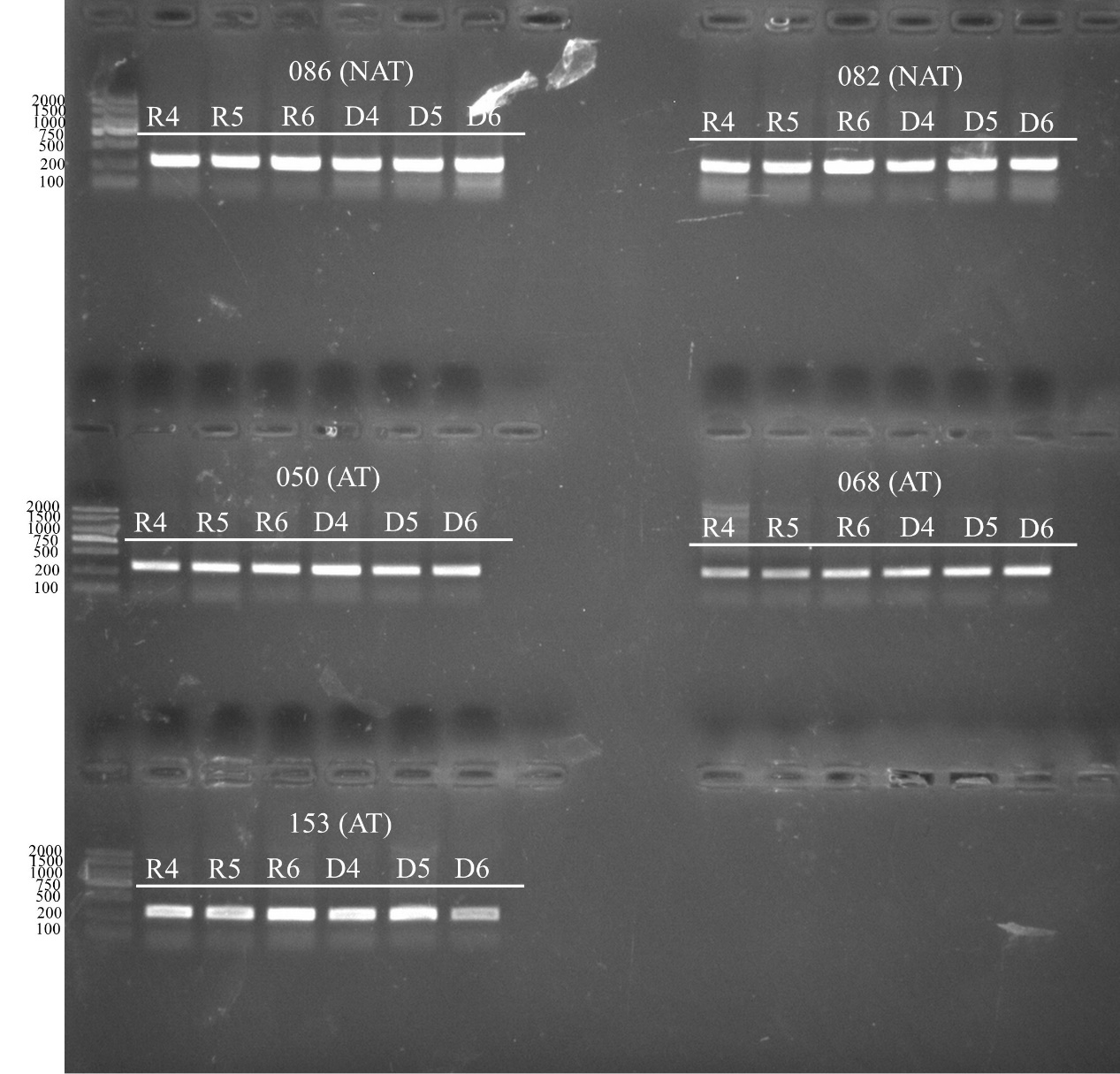
*

Figure S7-41. Expression analysis of *AP1.*

*
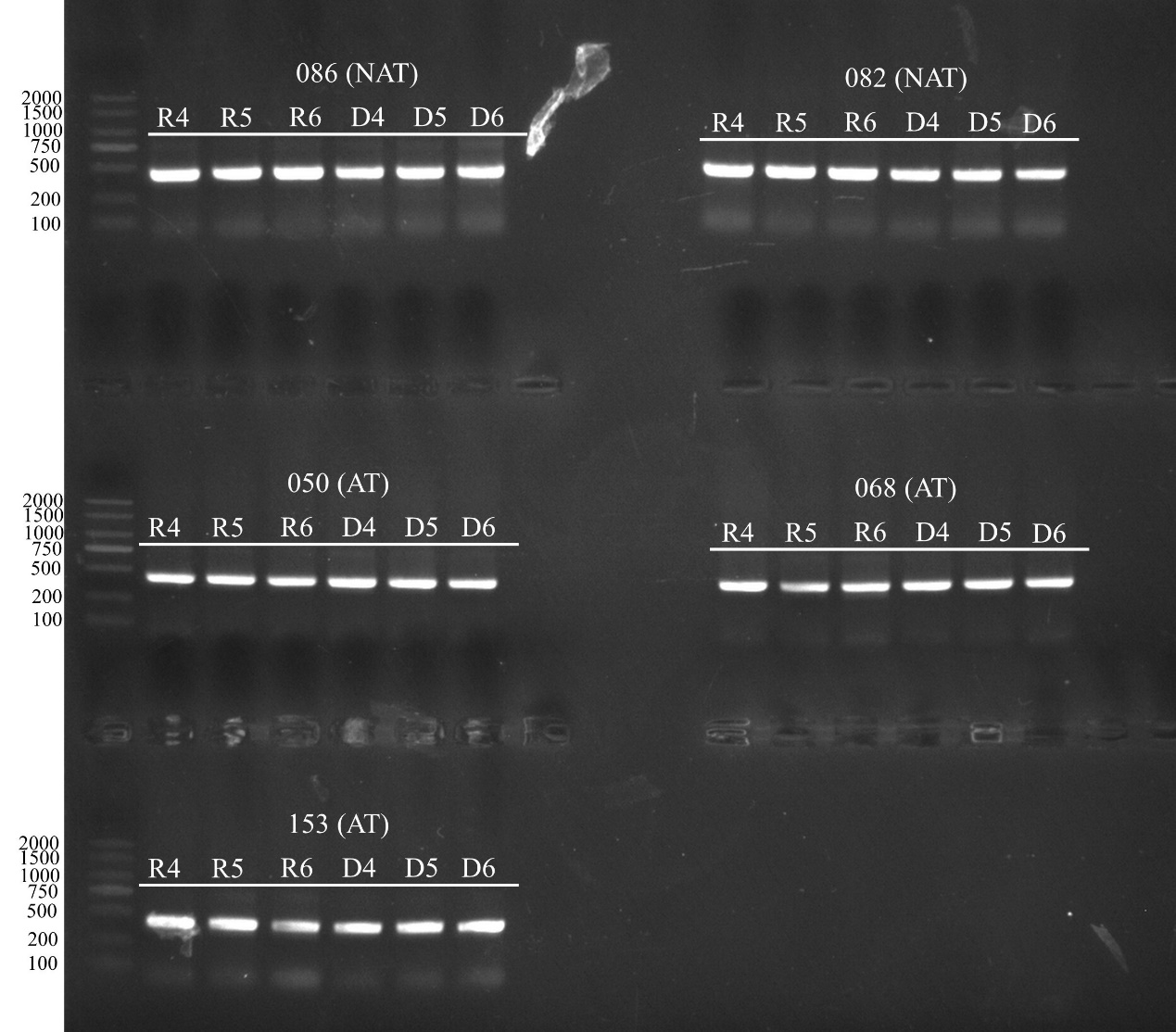
*

Figure S7-42. Expression analysis of *bHLH144.*

*
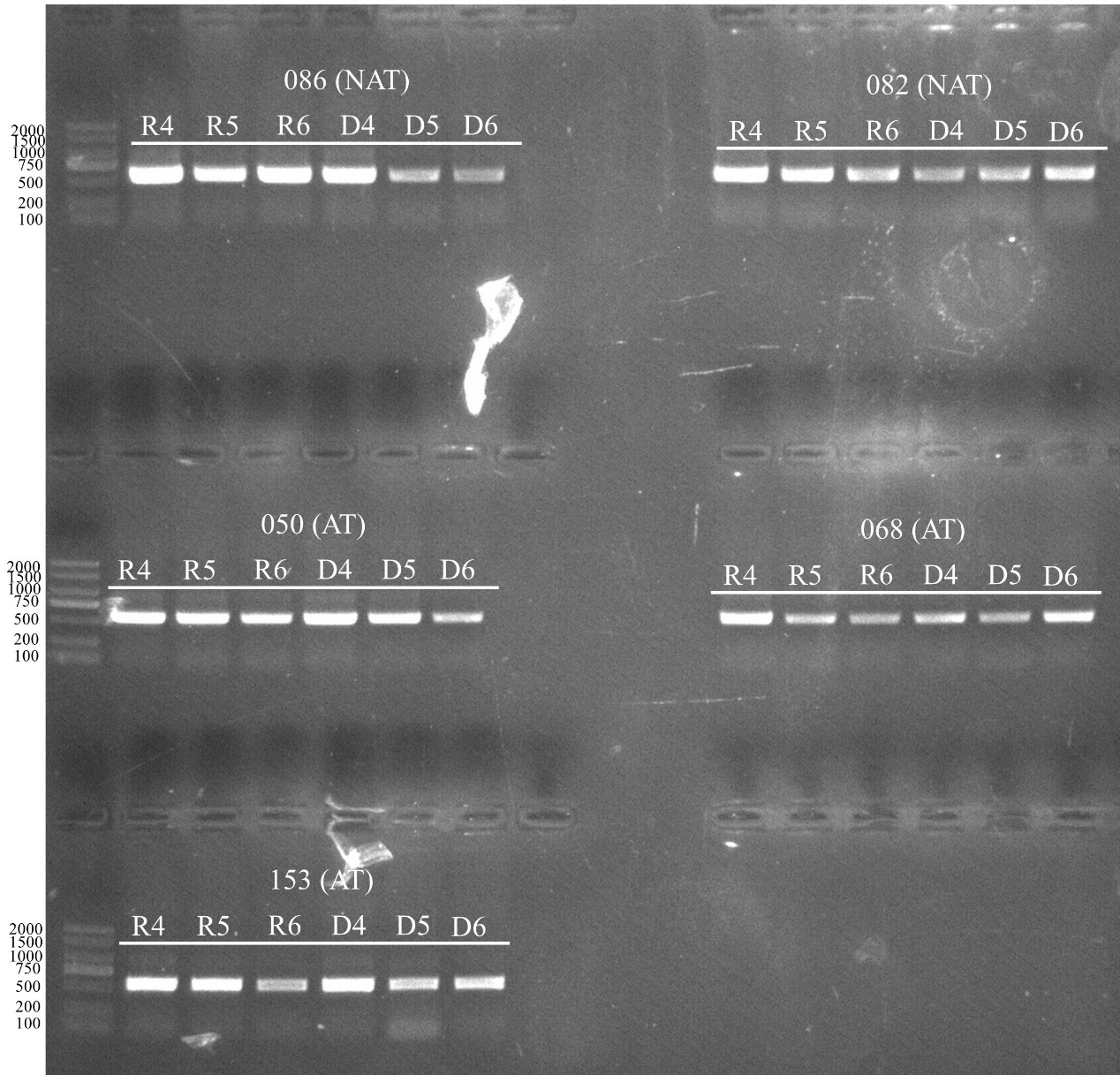
*

Figure S7-43. Expression analysis of *MYC2-*like*.*

*
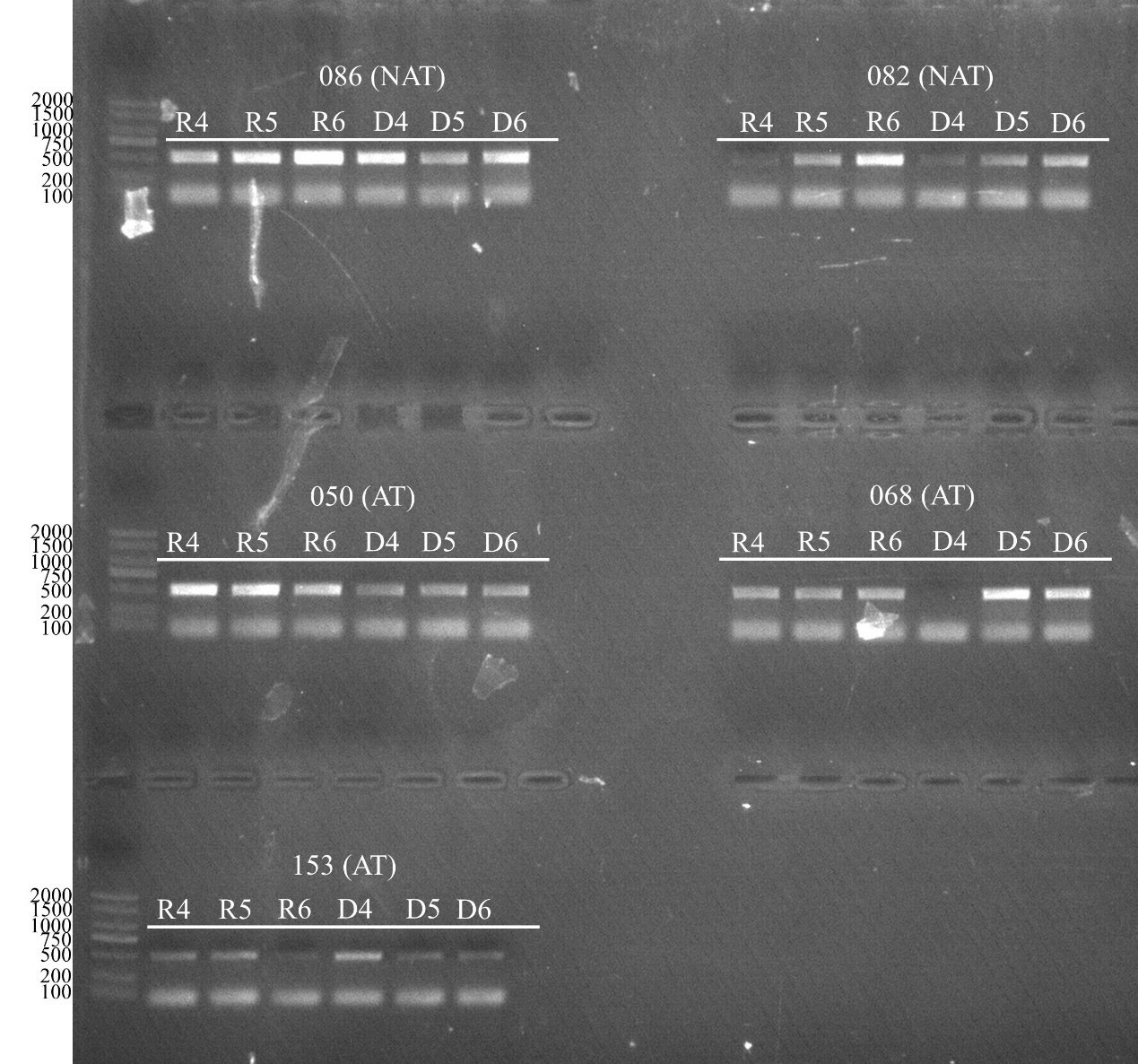
*

Figure S7-44. Expression analysis of *TCP2.*

*
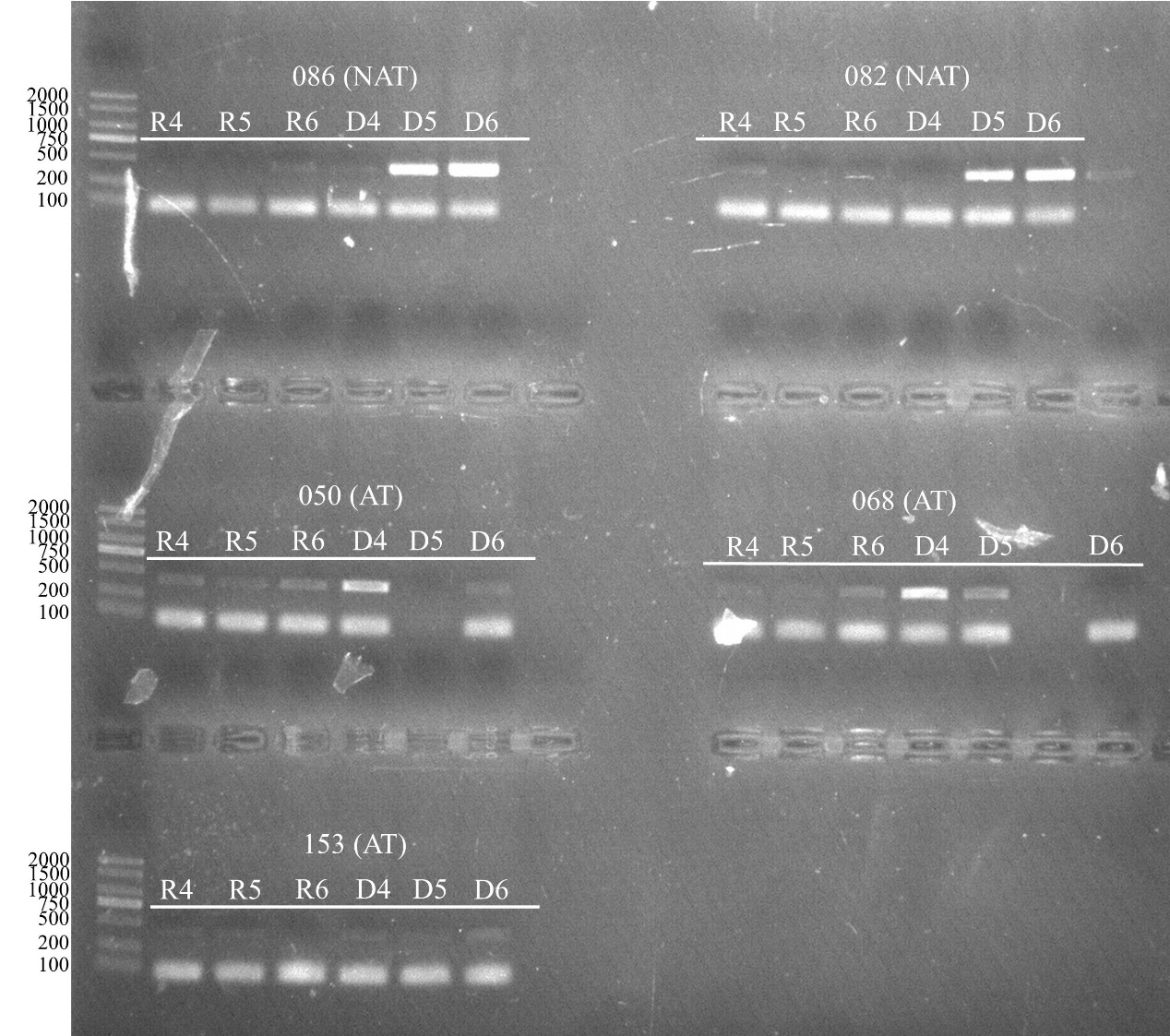
*

Figure S7-45. Expression analysis of *MYB108.*

*
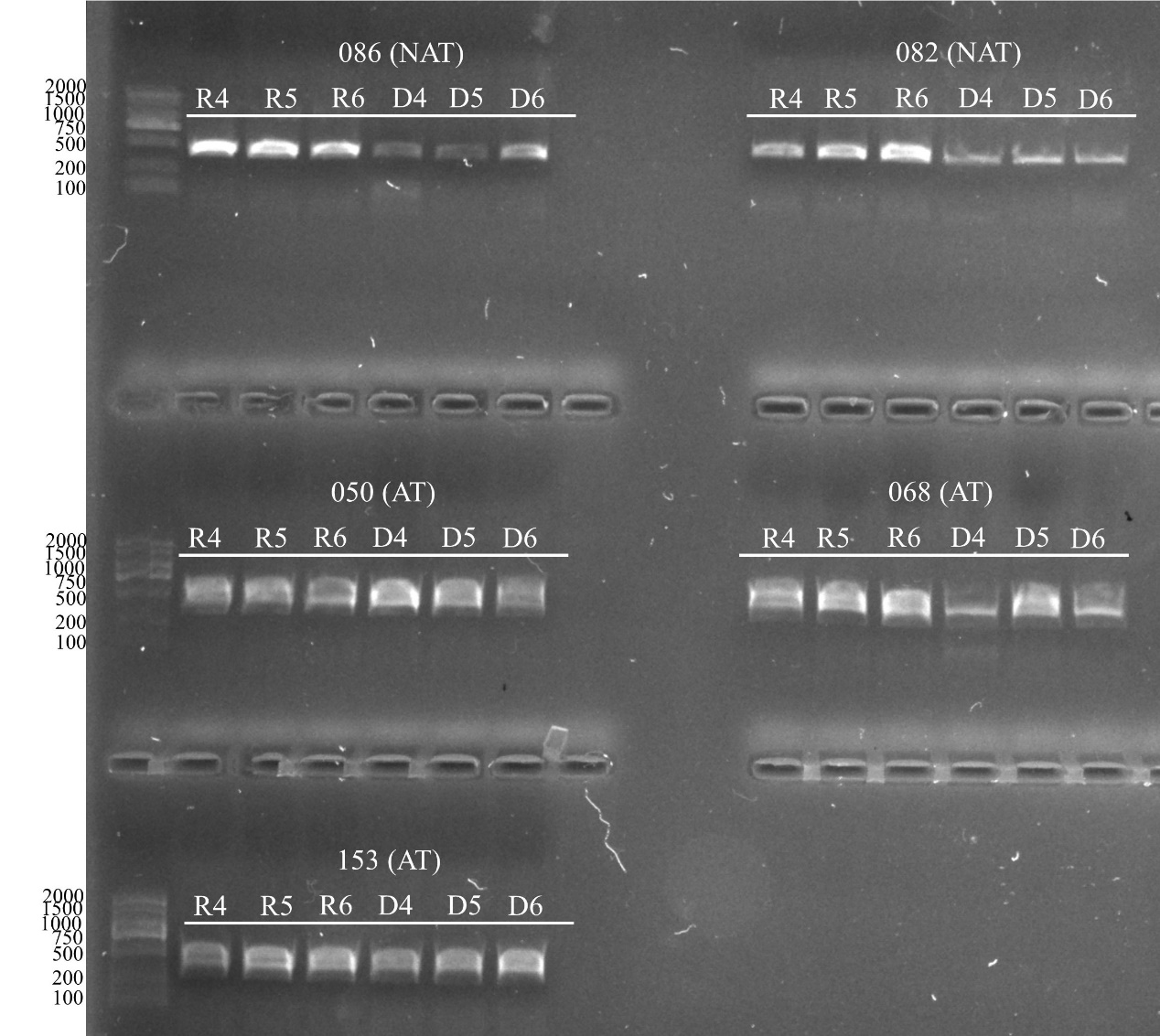
*

Figure S7-46. Expression analysis of *CDM104.*

*
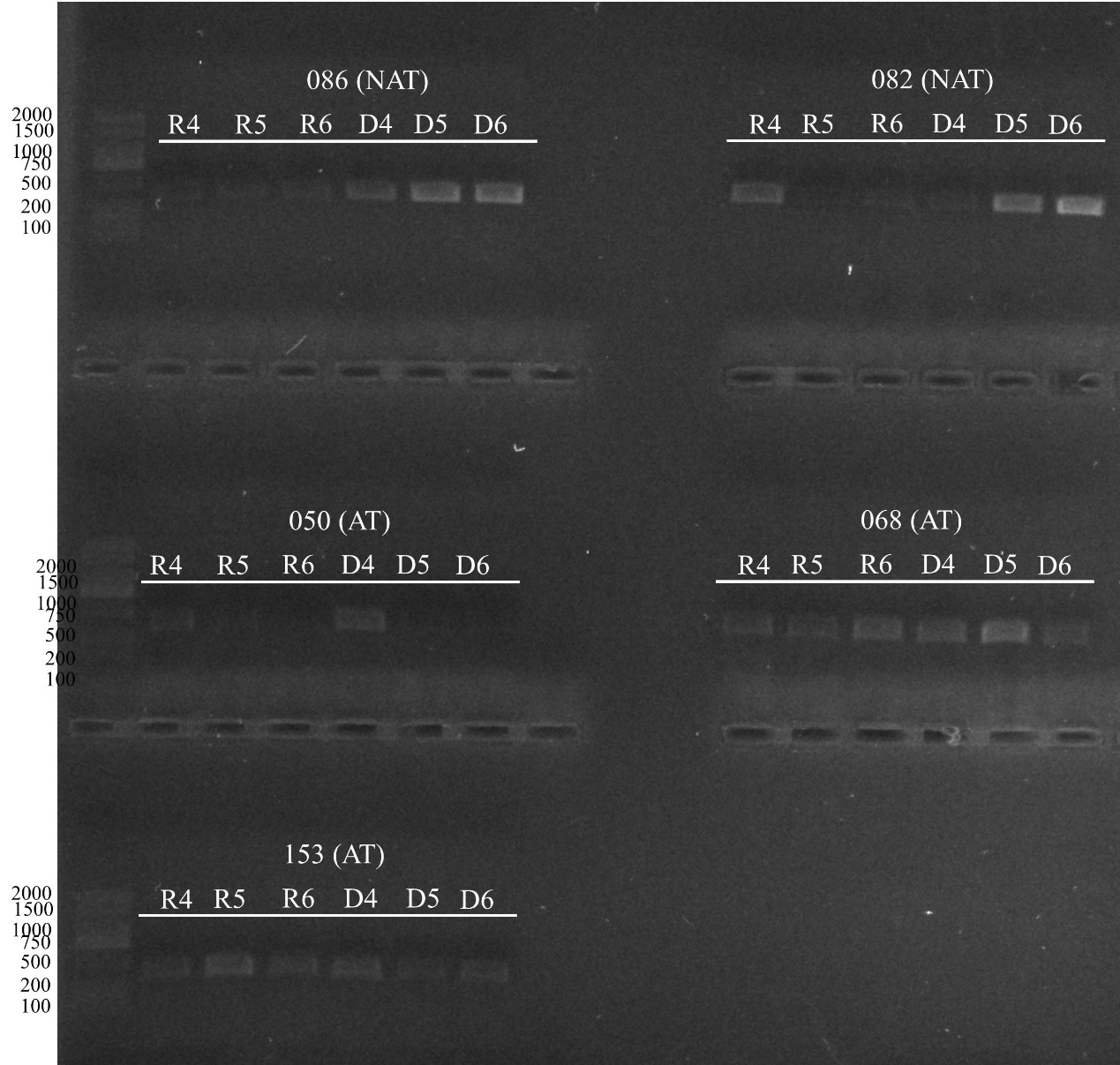
*

Figure S7-47. Expression analysis of *NGA1.*

*
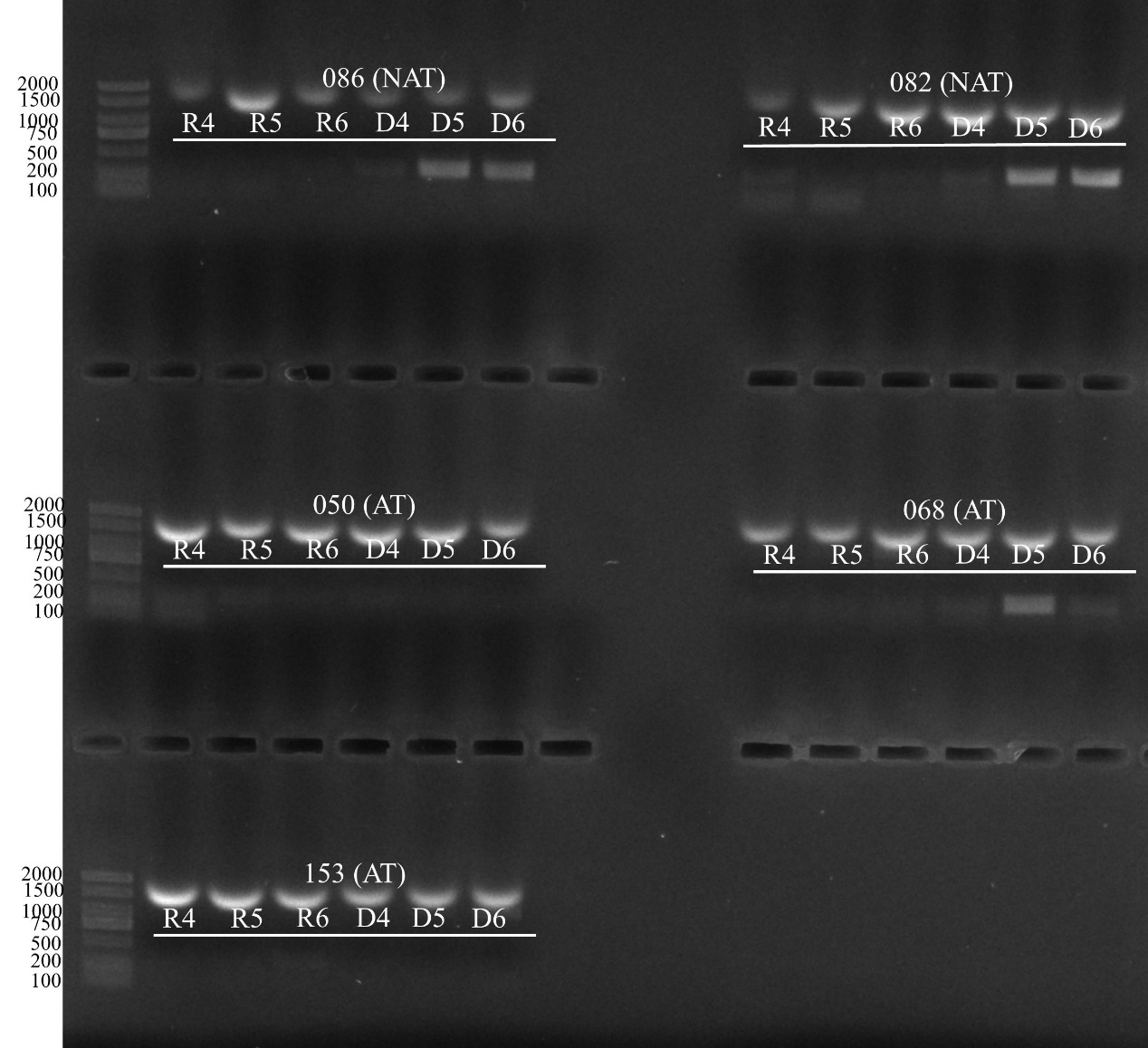
*

Figure S7-48. Expression analysis of *SVP.*

*
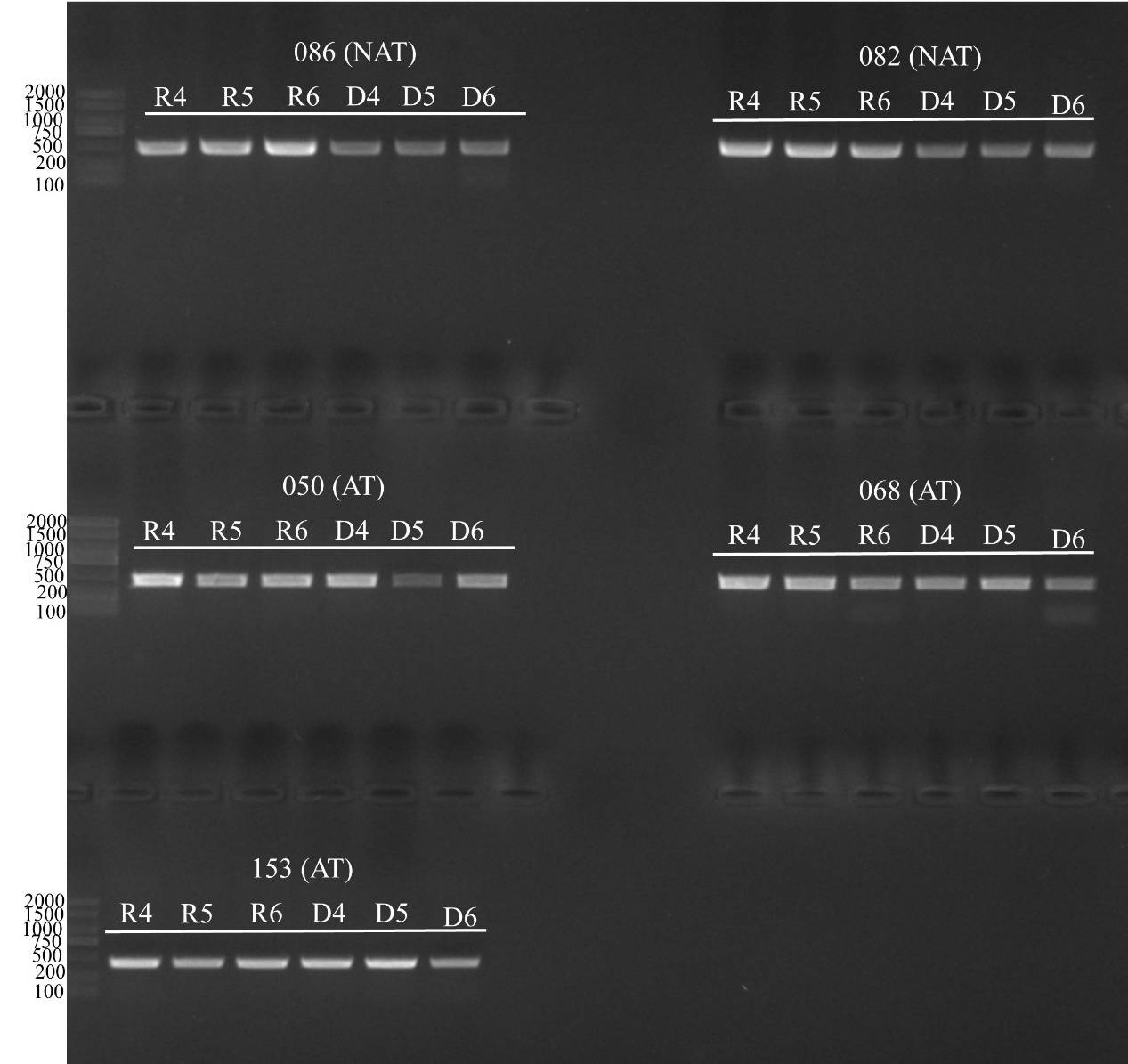
*

Figure S7-49. Expression analysis of *ARF4.*

*
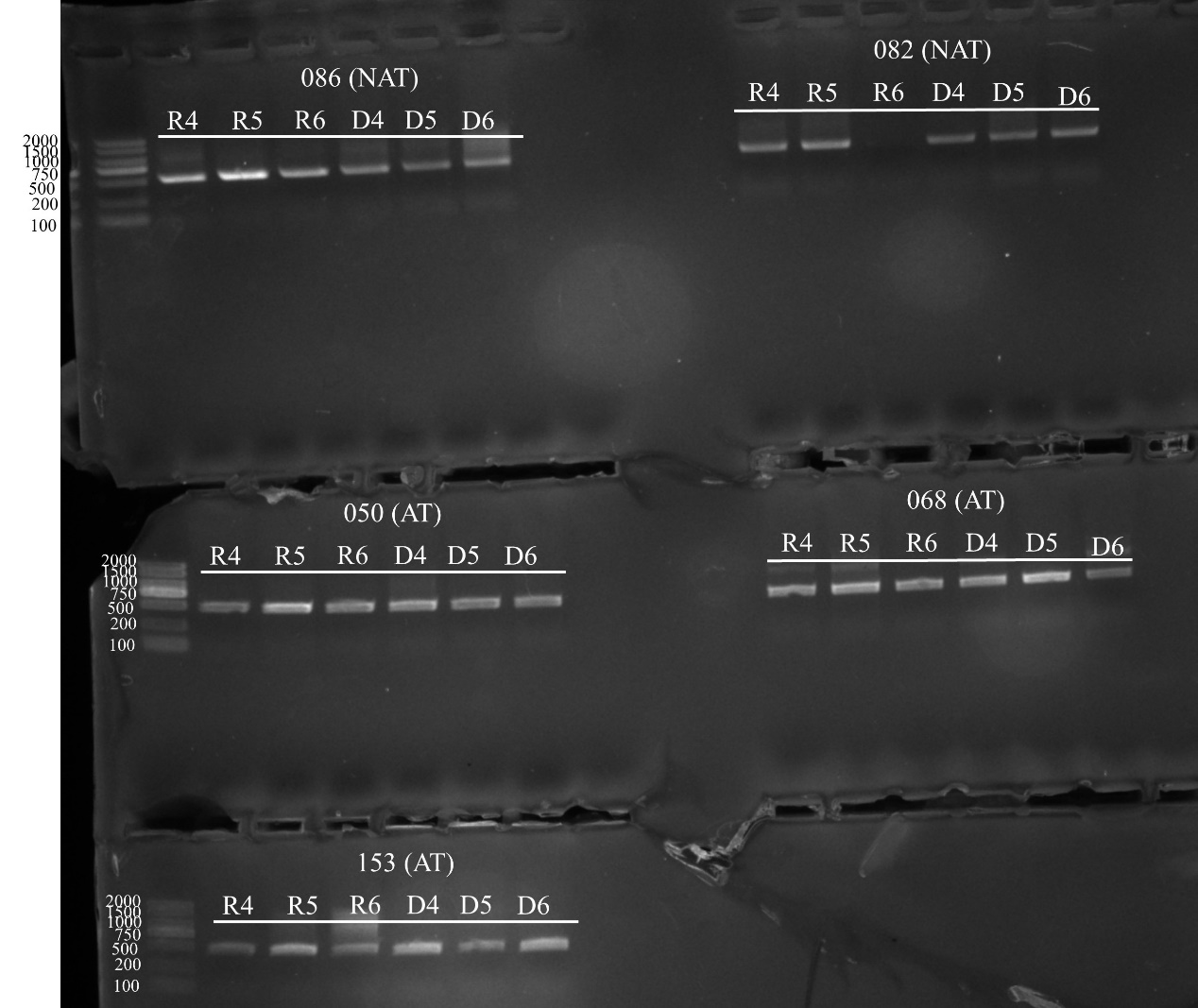
*

Figure S7-50. Expression analysis of *ARF2.*

*
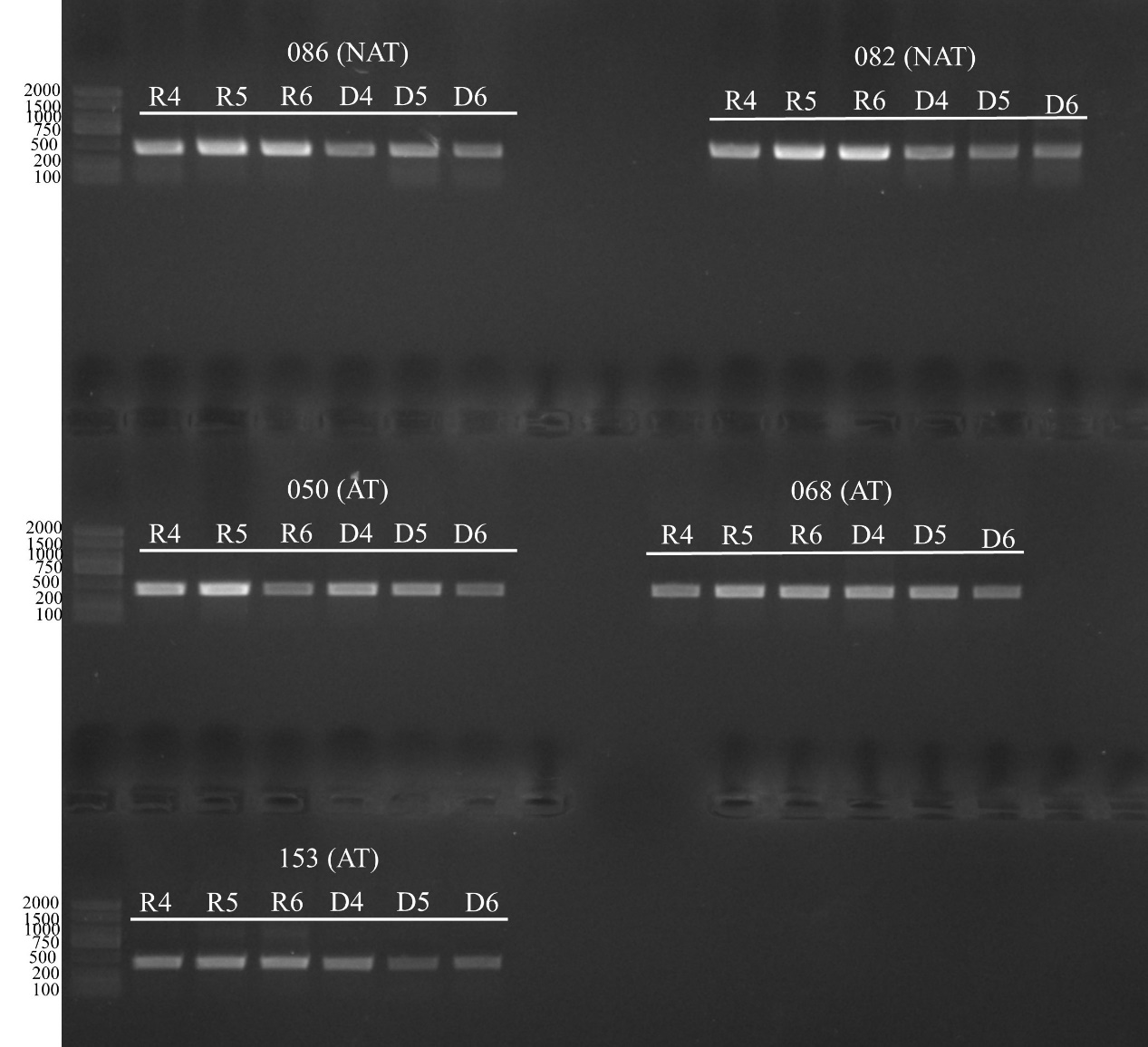
*

Figure S7-51. Expression analysis of *AUX28.*

*
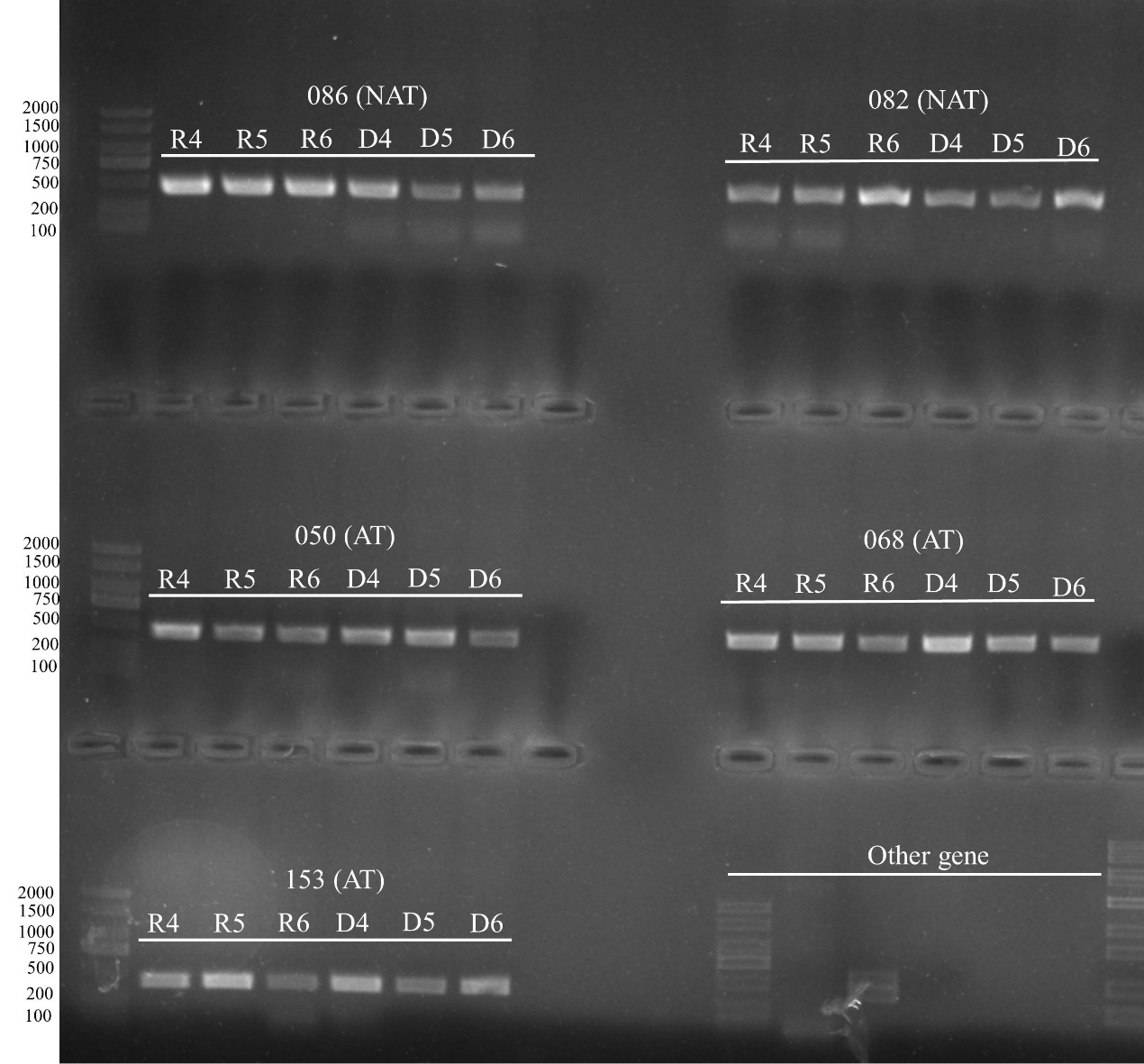
*

Figure S7-52. Expression analysis of *TCP8.*

*
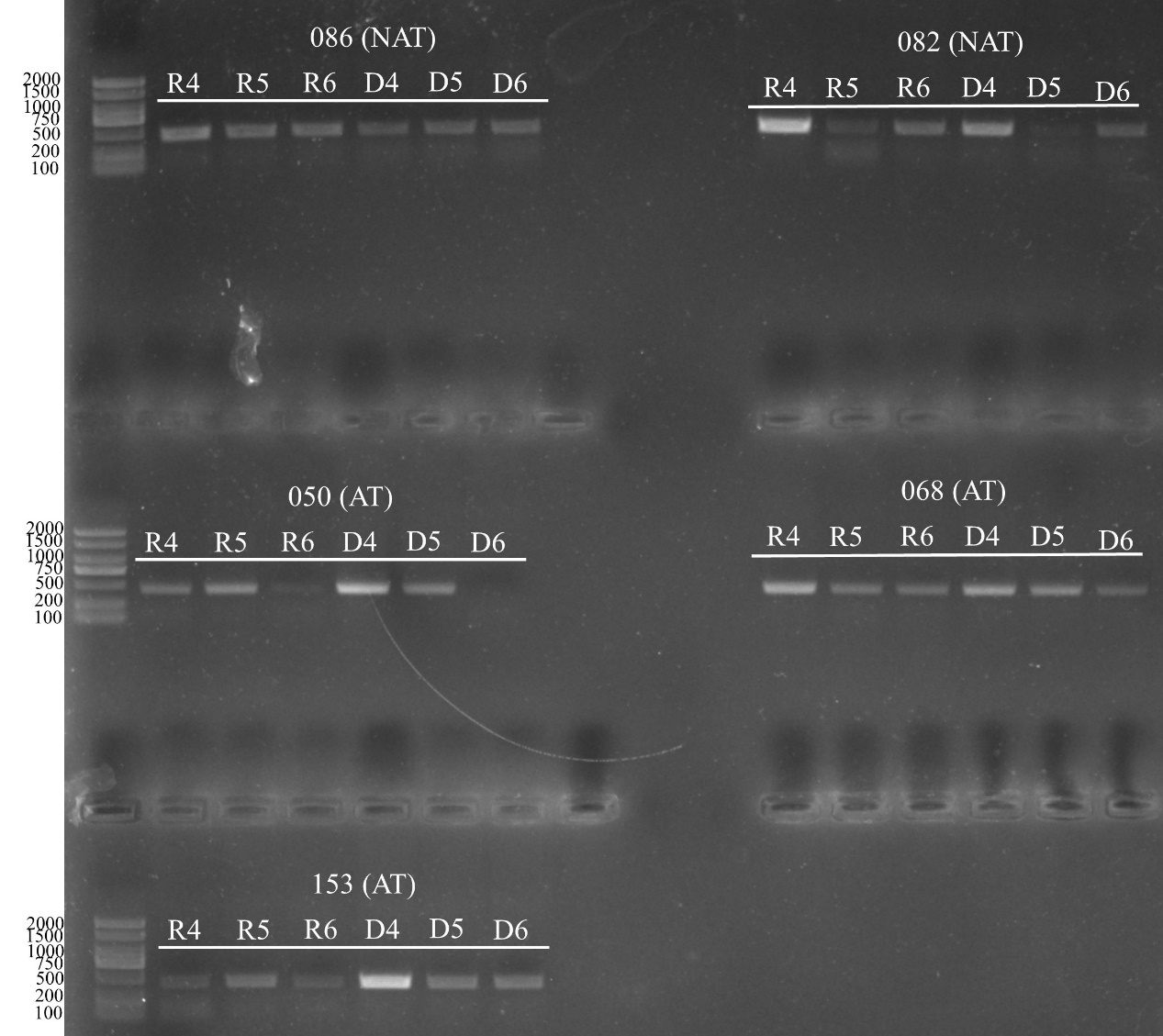
*

Figure S7-53. Expression analysis of *Zinc finger protein 6.*

*
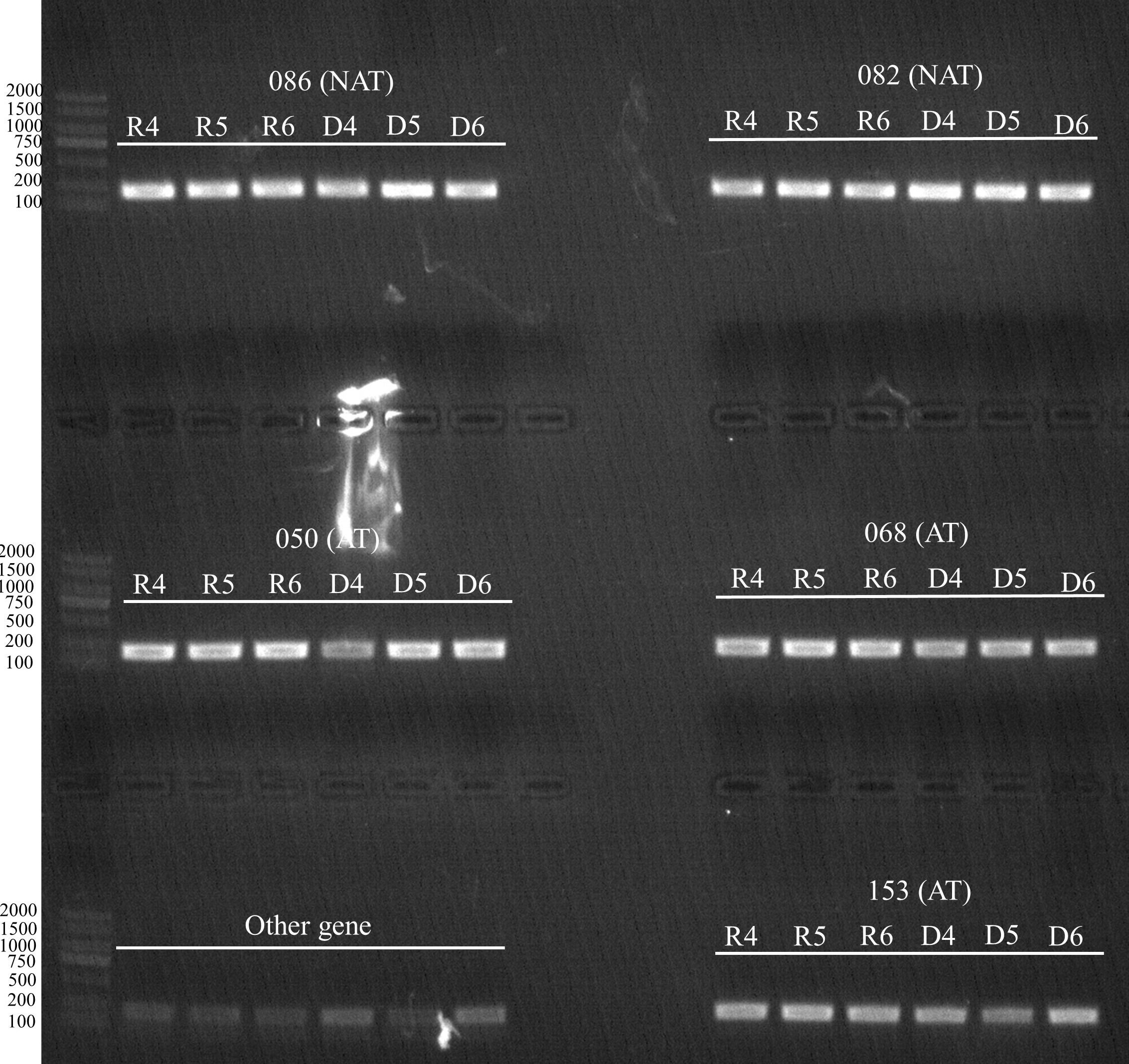
*

Figure S7-54. Expression analysis of *26S.*

*.*
