# Supplementary material for: Transcriptomic analysis reveals the formation mechanism of anemone-type flower in chrysanthemum
Source: BMC Genomics. 2022 Dec 22;23:846. doi: 10.1186/s12864-022-09078-3 (PMC9773529; doi:10.1186/s12864-022-09078-3)
Supplement: Supplementary file 11 — Additional file 11: Figure S7. RT-PCR analysis of DEGs in two non-anemone-type (082 and 086) and three anemone-type (050, 068, and 153) chrysanthemums using RT-PCR. [file 12864_2022_9078_MOESM11_ESM.doc]

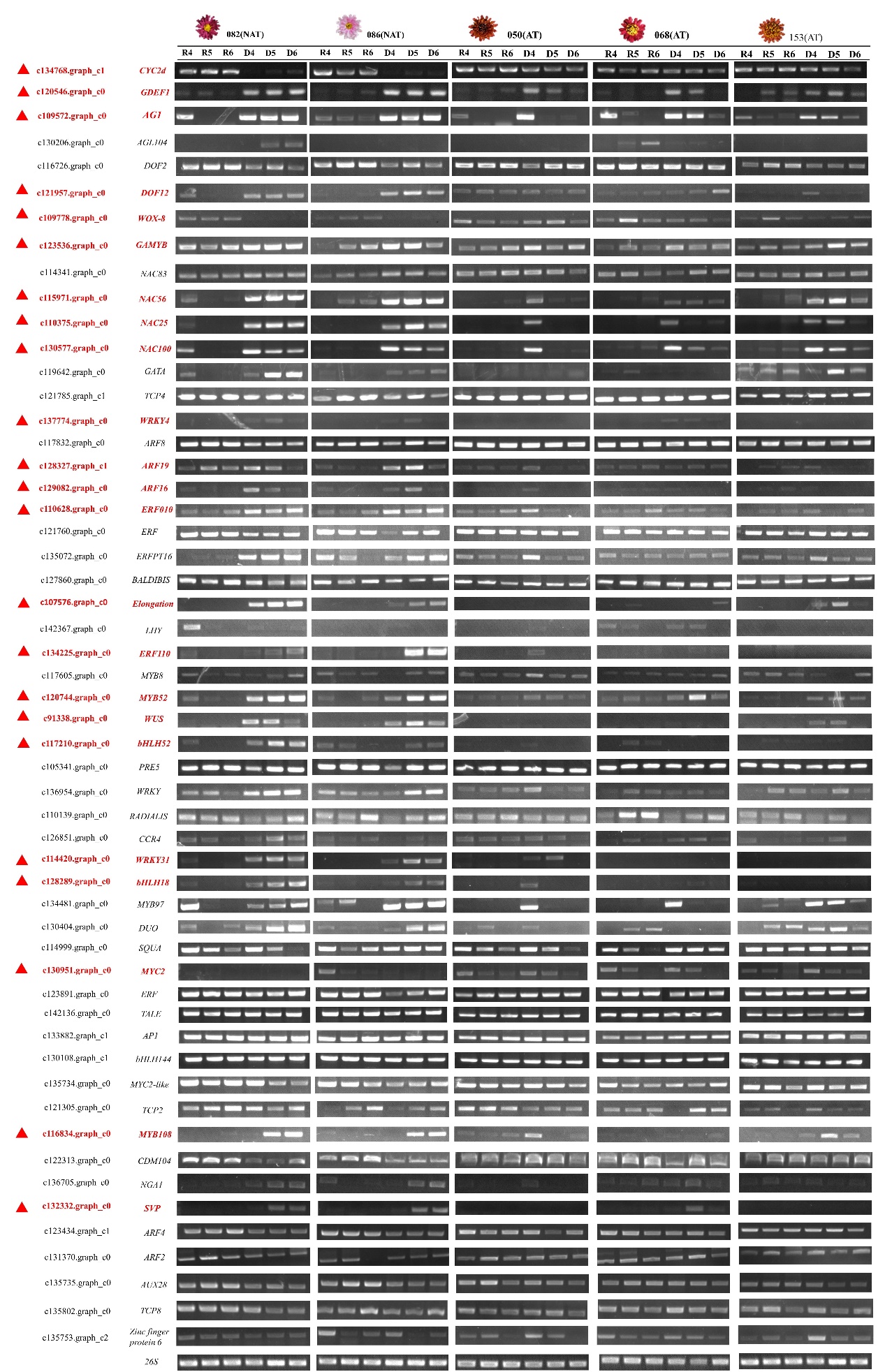


**Additional file 11: Figure S7.** RT-PCR analysis of DEGs in two non-anemone-type (082 and 086) and three anemone-type (050, 068, and 153) chrysanthemums using RT-PCR. The expression level of *26S* is used to normalize the mRNA levels for each sample. R4-R6 indicated the three opening stages of ray floret petal, D4-D6 indicated the three opening stages of disc floret. NAT: non-anemone-type AT: anemone-type.
