# Supplementary material for: Transcriptomic analysis reveals the formation mechanism of anemone-type flower in chrysanthemum
Source: BMC Genomics. 2022 Dec 22;23:846. doi: 10.1186/s12864-022-09078-3 (PMC9773529; doi:10.1186/s12864-022-09078-3)
Supplement: Supplementary file 12 — Additional file 12: Figure S8. qRT-PCR analysis of 15 DEGs in three non-anemone-type (082, 086, and PF) and four anemone-type (050, 068, GS, FK) chrysanthemums. [file 12864_2022_9078_MOESM12_ESM.doc]

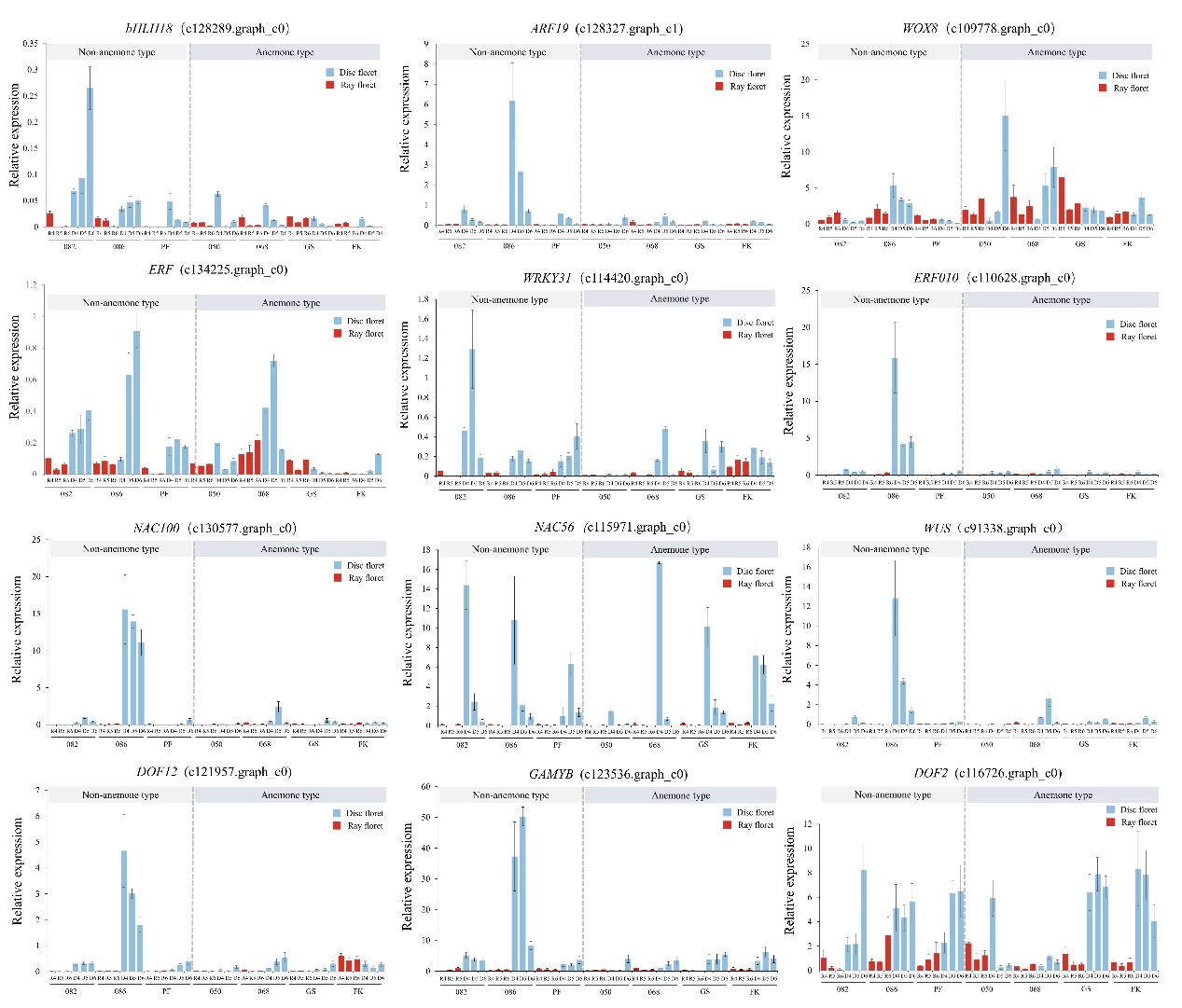


**Additional file 12: Figure S8.** qRT-PCR analysis of 15 DEGs in three non-anemone-type (082, 086, and PF) and four anemone-type (050, 068, GS, FK) chrysanthemums. R: Ray floret D: Disc floret R4-R6: ray floret at different opening stages. D4-D6: disc floret at different opening stages.
