# Supplementary material for: Transcriptomic analysis reveals the formation mechanism of anemone-type flower in chrysanthemum
Source: BMC Genomics. 2022 Dec 22;23:846. doi: 10.1186/s12864-022-09078-3 (PMC9773529; doi:10.1186/s12864-022-09078-3)
Supplement: Supplementary file 13 — Additional file 13: Figure S9. FPKM of 10 final DEGs in non-anemone-type (082) and anemone-type (068) chrysanthemums. [file 12864_2022_9078_MOESM13_ESM.doc]

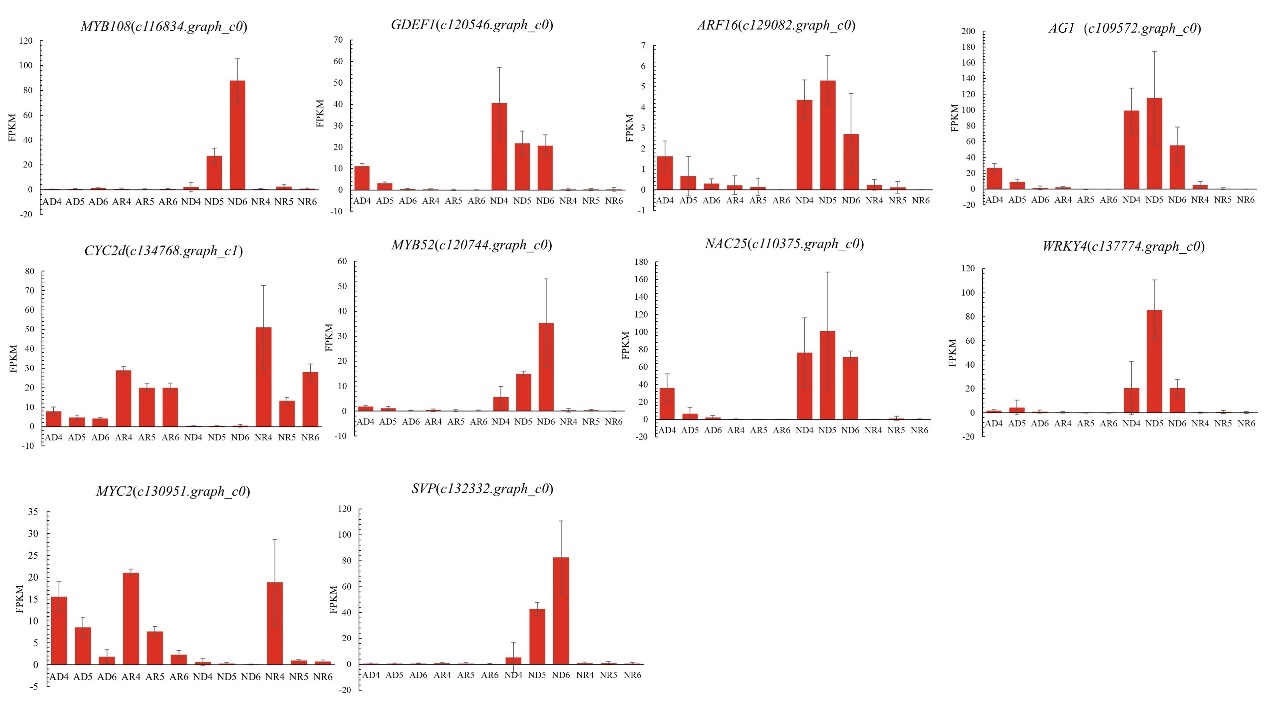


**Additional file 13: Figure S9.** FPKM of 10 final DEGs in non-anemone-type (082) and anemone-type (068) chrysanthemums. R: Ray floret D: Disc floret R4-R6: ray floret at different opening stages. D4-D6: disc floret at different opening stages.
