# Supplementary material for: Transcriptomic analysis reveals the formation mechanism of anemone-type flower in chrysanthemum
Source: BMC Genomics. 2022 Dec 22;23:846. doi: 10.1186/s12864-022-09078-3 (PMC9773529; doi:10.1186/s12864-022-09078-3)
Supplement: Supplementary file 14 — Additional file 14: Figure S10. The stamen of AT and NAT disc floret. [file 12864_2022_9078_MOESM14_ESM.doc]

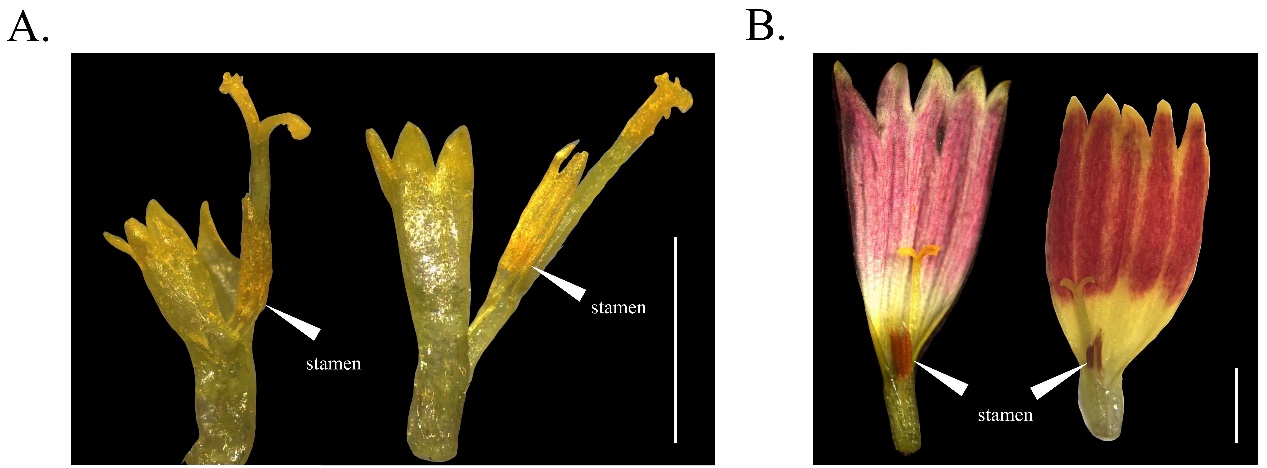


**Additional file 14: Figure S10.** The stamen of AT and NAT disc floret. A. the stamen of NAT disc florets B. the stamen of AT disc florets. scale bar=2.5 mm.
